# Construction of a High-Density Microsatellite Genetic Linkage Map and Mapping of Sexual and Growth-Related Traits in Half-Smooth Tongue Sole (*Cynoglossus semilaevis*)

**DOI:** 10.1371/journal.pone.0052097

**Published:** 2012-12-20

**Authors:** Wentao Song, Yangzhen Li, Yongwei Zhao, Yang Liu, Yuze Niu, Renyi Pang, Guidong Miao, Xiaolin Liao, Changwei Shao, Fengtao Gao, Songlin Chen

**Affiliations:** 1 Yellow Sea Fisheries Research Institute, Chinese Academy of Fishery Sciences, Qingdao, China; 2 Weihai Vocational College, Department of Biological and Chemical Engineering, Weihai, China; 3 College of Fisheries and Life Science, Shanghai Ocean University, Shanghai, China; Auburn University, United States of America

## Abstract

High-density genetic linkage maps of half-smooth tongue sole were developed with 1007 microsatellite markers, two SCAR markers and an F1 family containing 94. The female map was composed of 828 markers in 21 linkage groups, covering a total of 1447.3 cM, with an average interval 1.83 cM between markers. The male map consisted of 794 markers in 21 linkage groups, spanning 1497.5 cM, with an average interval of 1.96 cM. The female and male maps had 812 and 785 unique positions, respectively. The genome length of half-smooth tongue sole was estimated to be 1527.7 cM for the females and 1582.1 cM for the males. Based on estimations of the map lengths, the female and male maps covered 94.74 and 94.65% of the genome, respectively. The consensus map was composed of 1007 microsatellite markers and two SCAR markers in 21 linkage groups, covering a total of 1624 cM with an average interval of 1.67 cM. Furthermore, 159 sex-linked SSR markers were identified. Five sex-linked microsatellite markers were confirmed in their association with sex in a large number of individuals selected from different families. These sex-linked markers were mapped on the female map LG1f with zero recombination. Two QTLs that were identified for body weight, designated as We-1 and We-2, accounted for 26.39% and 10.60% of the phenotypic variation. Two QTLs for body width, designated Wi-1 and Wi-2, were mapped in LG4f and accounted for 14.33% and 12.83% of the phenotypic variation, respectively. Seven sex-related loci were mapped in LG1f, LG14f and LG1m by CIM, accounting for 12.5–25.2% of the trait variation. The results should prove to be very useful for improving growth traits using molecular MAS.

## Introduction

Genetic linkage maps have become important tools in many areas of genetic research. To perform a linkage study, it is necessary to genotype and map large numbers of the available genetic markers on mapping families. Microsatellites comprise an excellent choice for genomic mapping due to their abundance in most of the vertebrate genomes, including the genomic distribution, high polymorphism and ease of typing via PCR. Meanwhile, the simple sequence repeat (SSR) alleles are typically co-dominant, and their polymorphisms can be scored in either a simple polyacrylamide gel separation format or with high-throughput capillary arrays. Genetic linkage maps based on microsatellite markers have been generated for economically important marine species, such as salmon [1], tilapia [2], European sea bass [3], rainbow trout [4], sea bream [5], Barramundi [6], catfish [7], grass carp [8], Japanese flounder [9] and Asian sea bass [10].

The traditional methods of genetic improvement of quantitative traits have relied mainly on phenotype and pedigree information [11], which are easily influenced by environmental factors. It is generally accepted that marker-assisted selection (MAS) accelerates genetic improvement in a relatively short period, especially when the target characteristics are disease-related and there is a sufficient amount of observed genetic variation in a given trait. A genetic map constructed from a population segregated for a trait of interest is required for QTL identification. Information on genetic markers associated with QTL can be used in MAS breeding programs to identify and select individuals carrying desired traits. QTL mapping in commercial fishes is still in its infancy [12]. QTL for growth, disease resistance and stress response have been mapped in only a few species, such as Asian sea bass [10], rainbow trout [13], tilapia [14], salmon [15], Japanese flounder [16], guppy [17] and European seabass [18].

Half-smooth tongue sole (*Cynoglossus semilaevis*) is a commercially valuable flatfish that is widely distributed in Chinese coastal waters. Due to its appealing taste, commercial value, easy domestication and natural resource depletion, half-smooth tongue sole has been selected as a promising species for aquaculture [19]. It has been one of the most popular marine species used in aquaculture in China, together with turbot (*Scophthalmus maximus*), olive flounder (*Paralichthys olivaceus*), Spotted halibut *(Verasper variegatus*), and barfin flounder (*Verasper moseri*). Based on chromosome karyotype analysis, the karyotype of half-smooth tongue sole was determined to be 2 n = 42 t, NF = 42 [20]. After further study based on G-banding patterns analysis, it was confirmed that, in addition to having 20 euchromosome pairs, there was a pair of sex chromosomes (chromosome Z and W), and sex in this species is determined by a WZ/ZZ chromosomal system [20]. In addition, half-smooth tongue sole females grow two to three times larger and faster than males. Therefore, these characteristics suggest that half-smooth tongue sole has great potential for the production of all-female stock, as well as for studying the mechanisms of both genome evolution and sex determination [19].

Half-smooth tongue sole breeding is still in its infancy. Breeding efforts are complicated by the fact that most traits of economic significance exhibit quantitative inheritance [21]. Half-smooth tongue sole breeding community lacks a detailed genetic linkage map to facilitate the breeding process. Recently, number of genetic studies in this species have been reported, including microsatellite markers [22]–[28] and female-specific DNA markers [29], the construction of BAC libraries [30], molecular marker-assisted sex control [31], the characterization of certain sex-related genes [32] and artificial gynogenesis [33]. Furthermore, a low-density genetic linkage map was first constructed for half-smooth tongue sole by Liao et al. [19]. However, as a result this map has provided very little information on the genomic organization of this important marine species. Like other aquaculture species, the production of half-smooth tongue sole is often affected by outbreaks of deadly infectious diseases caused by bacteria, viruses or protozoan pathogens. To accelerate the genetic improvement needed to achieve large-scale aquaculture success, genetic studies in half-smooth tongue sole such as MAS based on QTL, are needed. Therefore, linkage maps need to be built mainly with co-dominant markers, which are representative of the same loci across studies.

In the present study, we constructed a high-density microsatellite genetic linkage map in half-smooth tongue sole. At the same time, sex-linked microsatellite markers were identified on the linkage maps. In addition, four growth rate QTLs and seven sex-related loci were mapped on the genetic linkage map and some of them should prove to be useful in MAS in future breeding programs in half-smooth tongue sole. These linkage maps represent a powerful tool both for research on genome evolution and for brood stock enhancement programs using MAS breeding in half-smooth tongue sole, and will help facilitate genome mapping efforts in other species of flatfish.

## Results

### Genetic Markers

To obtain useful microsatellite markers for linkage analysis, we examined the segregation patterns of 4452 markers in the mapping family. Among the 4452 markers, 1317 (29.6%) were polymorphic in the mapping family. The sequence data of 951 newly identified polymorphic microsatellites were deposited with the GenBank Data Library under the accession nos: JN902087 - JN903037. A list of the 1317 polymorphic microsatellite markers is presented in Table S1. The microsatellite locus derived from the sequence of the *SOX9* genes in half-smooth tongue sole was linked to LG18. Gene072 (Toll-like receptor 9, FJ418072) and ghrh-ssr (PACAP-related peptide, FJ608666) were linked to LG11 and LG21, respectively. In addition, gene177 (Ovarian aromatase, EF421177) and gene116 (Myostatin, EF683116) were only polymorphic for the female parent in the mapping family, and were mapped in the linkage groups LG6f and LG17f, respectively.

### Sex-linked Markers

**Figure 1:**
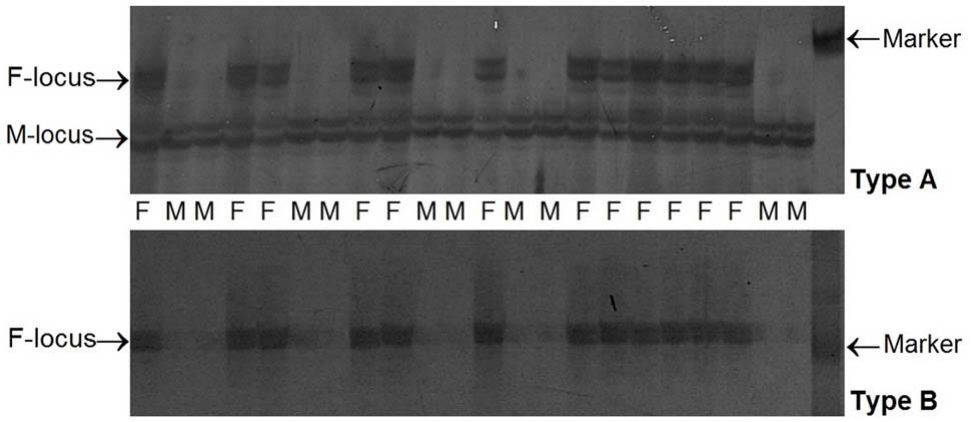
Polyacrylamide gel separation of sex-linked microsatellites PCR amplification products in females and males. Type A: Marker F-locus was only present in females, and marker M-locus present in females and males. Type B: Marker F-locus was only present in females, and marker M-locus absent in all individuals.

**Figure 2:**
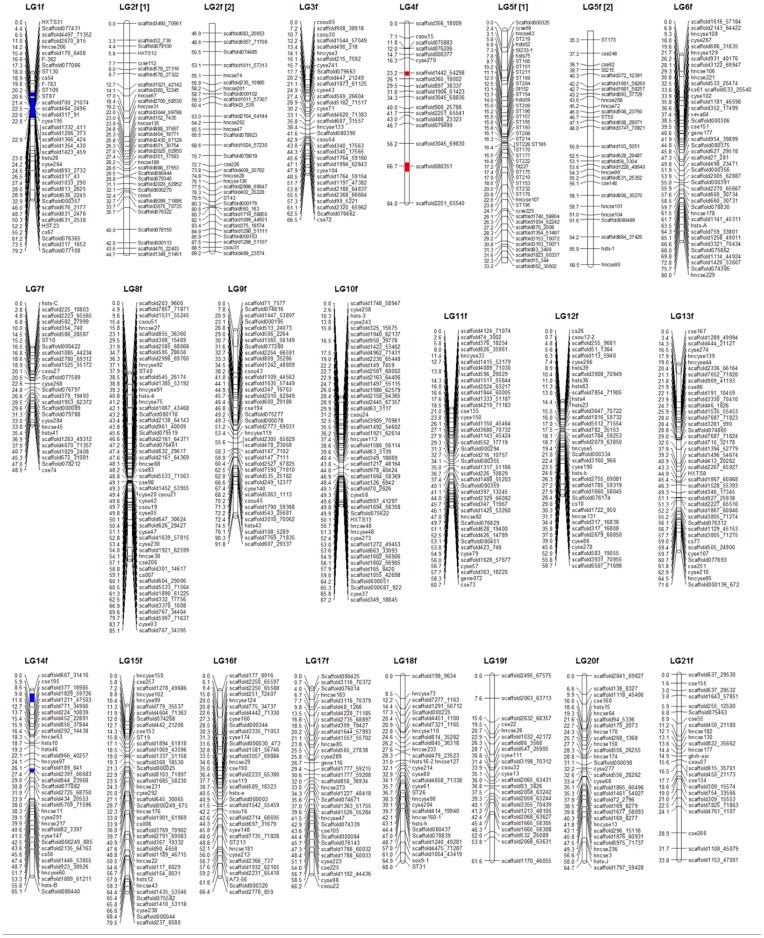
Linkage maps of the female-specific map for *Cynoglossus semilaevis*. The female-specific genetic map comprises 828 markers assigned to 21 linkage groups (LG1f–LG21f). Genetic distances in Kosambi centimorgans are listed on the left side of the linkage groups, and markers are listed on the right side of the linkage groups.

Sex-specific molecular markers are a useful genetic resource for studying sex- determining mechanisms and controlling fish sex. In a previous study, a female-specific DNA marker was located on the linkage map of half-smooth tongue sole. That was the first report on the mapping of a sex-linked marker on a genetic linkage map in teleosts [19]. In the present study, 159 sex-linked SSR markers (JN902124–JN902282) were identified and divided into two types (Types A and B, Figure 1). Five sex-linked microsatellite markers were confirmed in their association with sex in 96 individuals selected from different families and were found to be located in the LG1f region. Using PCR-based allele-specific assay adapted from F-382 and F-783, we mapped two sex-linked SCAR (sequence-characterized amplified region) markers to the female map LG1f, on which sex-linked SSR markers were located (Fig. 2). The presence of sex-linked markers suggested the possibility of female heterogamety (ZZ male; WZ female) in half-smooth tongue sole, which is confirmed by the presence of a large heteromorphic sex chromosome in the females of this species [19]. Therefore, LG1 should correspond to the sex chromosome.

### Linkage Analysis

A total of 1317 demonstrably heterozygous markers were available for mapping. Among these 1317 loci, 1138 markers were used to construct the female map, and 958 markers were used to construct the male map. Twenty-nine microsatellite markers did not exhibit any significant linkage to any other markers. Segregation distortion from that expected under Mendelian inheritance was found in 406 (30.7%) of 1317 microsatellite markers. As a result, 357 of these 406 markers were located the linkage map after linkage analysis.

When the total of 1317 effective microsatellite markers and two SCAR markers were analyzed, 1009 markers were on the linkage map containing 21 linkage groups (LGs) at a LOD threshold value of 4.0.

### Sex-specific Maps

**Figure 3:**
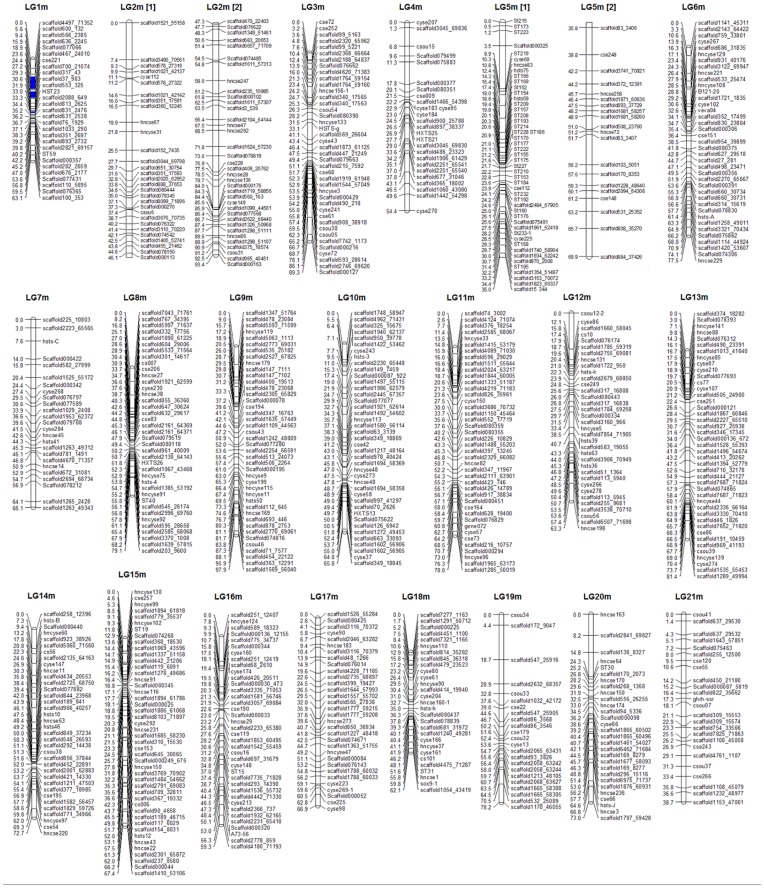
Linkage maps of the male-specific map for *Cynoglossus semilaevis*. The male-specific genetic map comprises 794 markers assigned to 21 linkage groups (LG1m–LG21m). Genetic distances in Kosambi centimorgans are listed on the left side of the linkage groups, and markers are listed on the right side of the linkage groups.

Significant linkages were identified for 1317 genetic markers, including a total of 1315 microsatellite loci and two SCAR markers. However, 305 microsatellite markers were unmapped in this analysis. Consequently, the mapping ratio of these markers is 76.8%. The female and male maps contained 828 and 794 markers, respectively (Fig. 2 and Fig. 3). Both maps were found to have 21 linkage groups, which is in agreement with the karyotype of 2 n = 42. The total length of the female map is 1447.3 cM, with an average interval of 1.83 cM. The linkage group size ranged from 33.8 cM to 91.8 cM. The number of loci per genetic linkage group varied from 18 to 74. The male linkage map spanned a total genetic distance of 1497.5 cM. The length of each linkage group varied from 38.7 to 97.9 cM and contained 23–68 loci per group, with an average interval of 1.96 cM. The sex-specific genetic linkage maps are presented in Figure 2 and Figure 3. The female and male maps display 812 and 785 unique positions, respectively. The estimated genome lengths, based on the two methods, were 1524.5 cM (Ge1) and 1531.2 cM (Ge2) for the female, and 1579.8 cM (Ge1) and 1584.4 cM (Ge2) for the male. The average of these two values was taken as the expected genome length, namely 1527.7 cM for the female and 1582.1 cM for the male. A summary of the genetic linkage maps of half-smooth tongue sole is shown in Table 1 and Table 2. Based on recent estimations of map length, the genomic coverage of the female and male maps were 94.74% and 94.65%, respectively.

**Table 1:** The characterization of linkage groups of half-smooth tongue sole.

Female maps			Male maps			Consensus maps		
LG	No. of markers	Length (cM)	LG	No. of markers	Length (cM)	LG	No. of markers	Length (cM)
1f	38	79.2	1 m	29	63.1	1	52	79.1
2f	68	89.2	2 m	66	93.4	2	83	101
3f	33	66.5	3 m	41	89.3	3	46	85.8
4f	18	84	4 m	26	54.4	4	29	47.9
5f	74	68.5	5 m	68	69.9	5	87	79.5
6f	43	80	6 m	39	77.5	6	47	78.9
7f	28	48.5	7 m	25	66.1	7	34	64.7
8f	55	85.1	8 m	39	79.1	8	57	94.8
9f	40	91.8	9 m	42	97.9	9	59	101.5
10f	49	67.2	10 m	41	65.8	10	51	68.9
11f	42	60.7	11 m	42	78	11	49	90.3
12f	39	58.7	12 m	35	63.3	12	45	79.2
13f	42	71.6	13 m	43	81.4	13	51	83.1
14f	36	65.1	14 m	36	72.7	14	46	73.8
15f	41	70.5	15 m	46	67.4	15	53	74.2
16f	36	66.4	16 m	38	59.3	16	46	69.4
17f	35	66.2	17 m	32	66.9	17	39	72.1
18f	31	68	18 m	29	62.1	18	37	68.9
19f	24	61.6	19 m	23	78.2	19	30	101.6
20f	31	64.7	20 m	29	73	20	36	72.1
21f	25	33.8	21 m	25	38.7	21	32	37.2
Total	828	1447.3	Total	794	1497.5	Total	1009	1624

**Table 2:** Summary of genetic linkage maps of half-smooth tongue sole.

	Female maps	Male maps	Consensus maps
Number of markers scored	1138	958	1317
Number of markers mapped	828	794	1009
Number of unique positions	812	785	991
Number of genetic linkage groups	21	21	21
Average number of markers per group	39	38	48
Minimum number of markers per group	18	23	29
Average marker spacing (cM)	1.83	1.96	1.67
Maximum length of group (cM)	91.8	97.9	101.6
Minimum length of group (cM)	33.8	38.7	37.2
Observed genome length (cM)	1447.3	1497.5	1624
Estimate genome length (cM)			
Ge1	1524.2	1579.8	1694.2
Ge2	1531.2	1584.4	1698.2
Ge	1527.7	1582.1	1696.2
Genome coverage %	94.74	94.65	95.74

### Consensus Map

**Figure 4:**
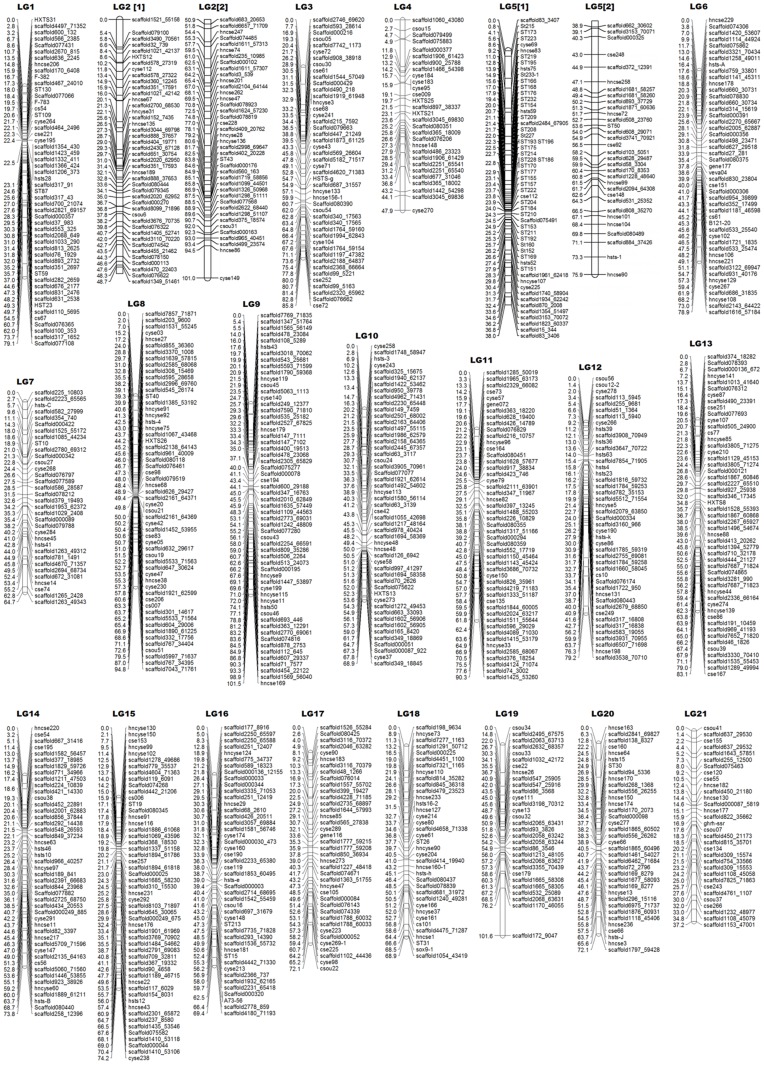
Linkage maps of the consensus map for *Cynoglossus semilaevis*. The consensus genetic map comprises 1009 markers assigned to 21 linkage groups (LG1–LG21). Genetic distances in Kosambi centimorgans are listed on the left side of the linkage groups, and markers are listed on the right side of the linkage groups.

Either bridge markers or homologous loci were used to identify the co-linear region in the female and male maps. The consensus map was composed of 1007 microsatellite markers and two SCAR markers in 21 linkage groups, covering a total of 1624 cM with an average interval of 1.67 cM (Fig. 4). The genome length of half-smooth tongue sole was estimated to be1698.2 cM, and the coverage of 95.74% was observed. This average estimated genome size is longer than the speculated 1451.3 cM length found by Liao et al. [19].The linkage group length varied from 37.2 cM to 101.6 cM, and the number of markers on the linkage group varied from 29 to 83.

### Recombination Rate

The availability of SSR markers in the male and female maps allowed an evaluation of the respective meiotic recombination rates. The recombination rates obtained from 21 linkage groups were on average 0.0183 in females and 0.0196 in males. Therefore, the relative recombination ratio (female-to-male; F/M) in these pairs was 1∶1.07, slightly higher in males than females.

The average recombination rate across all of the linkage groups is approximately 0.0163 in half-smooth tongue sole, which is higher than that in zebrafish [34], tilapia [2], catfish [7] and grass carp [8], and lower than rainbow trout [35], Asian sea bass [36] and Japanese flounder [9].

### QTL Associated with Growth Traits

**Figure 5:**
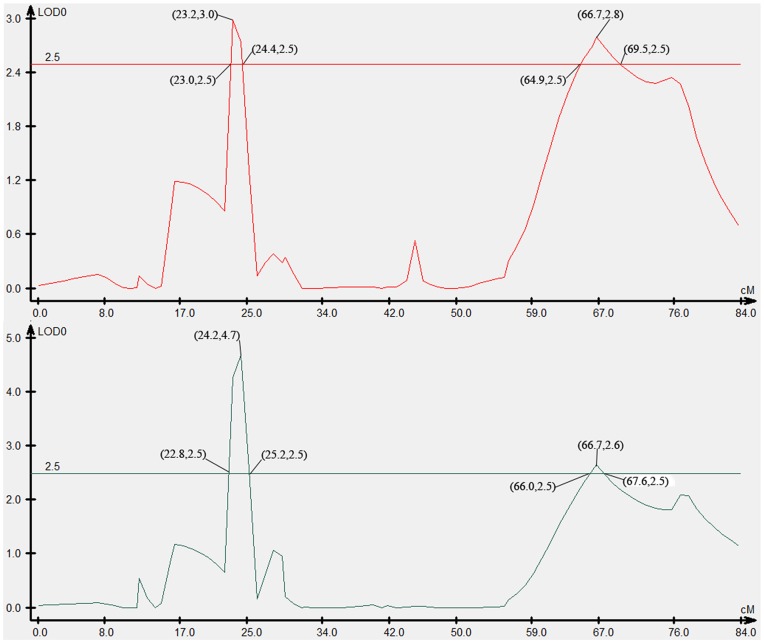
LOD curve graph of four growth-related QTLs. Abscissa indicates the relative position on the linkage groups, vertical coordinates indicates the value of LOD; the figure in “()” represents (position, value of LOD).

Four QTLs associated with growth traits were mapped in LG4f by CIM, accounting for 10.60–26.39% of the phenotypic variance (Fig. 2 and Fig. 5). The individual QTL which were detected were as follows: Two QTLs that were identified for body weight, designated as We-1 and We-2, explained 26.39% and 10.60% of the phenotypic variation. Two QTLs for body width, designated Wi-1 and Wi-2, were mapped in LG4f and explained 14.33% and 12.83% of the phenotypic variation, respectively. Two makers, Scaffold1442_54298 and Scaffold080351, were highly significantly (P<0.01) correlated with growth traits (Table 3).

**Table 3:** Biometrical parameters of individual QTL affecting growth traits and sex of half-smooth tongue sole.

Trait	QTL name	LG	Marker position	Associated markers	LOD scores	QTL Interval	R^2^ (%)
Body weight	We-1	LG4F	24.2	Scaffold1442_54298	4.7	22.8–25.2	26.39
	We-2	LG4F	66.7	Scaffold080351	2.6	66.0–67.6	10.60
Body width	Wi-1	LG4F	23.2	Scaffold1442_54298	3.0	34.4–38.0	14.33
	Wi-2	LG4F	66.7	Scaffold080351	2.8	72.6–75.1	12.83
Sex	S-1	1f	14.2	hncse206	3.7	11.6–14.3	11.5
	S-2	1f	15.4	scaffold170_6408	6.7	15.4–26.5	25.2
	S-3	14f	–	–	34.6	2.1–3.9	–
	S-4	14f	–	–	6.3	27.9–28.8	–
	S-5	1 m	15.9	scaffold636_2245	11.4	11.6–17.7	19.9
	S-6	1 m	23.6	scaffold467_24010	5.9	20.5–23.8	16.6
	S-7	1 m	–	–	3.7	32.6–33.1	–

R^2^ (%): proportion of the explained phenotypic variance.

LG: linkage group.

### Mapping of Sex-related Loci

**Figure 6:**
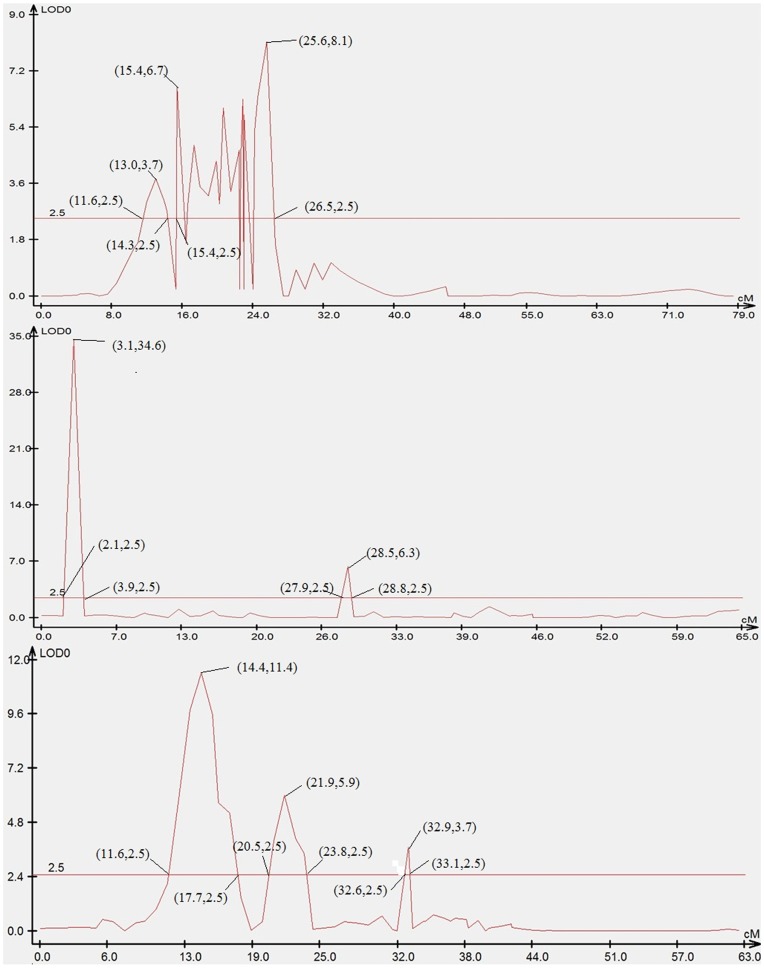
LOD curve graph of seven sex-related loci. Abscissa indicates the relative position on the linkage groups, vertical coordinates indicates the value of LOD; the figure in “()” represents (position, value of LOD).

Seven sex-related loci were mapped in LG1f, LG14f and LG1m by CIM, accounting for 12.5–25.2% of the trait variation (Fig. 2, Fig. 3 and Fig. 6). Four microsatellite makers, hncse206, scaffold170_6408, scaffold636_2245 and scaffold467_24010, were highly significantly (P<0.01) correlated with sex. (Table 3).

## Discussion

Linkage analysis and map construction using molecular markers is more complicated in full-sib families of out-breeding species than in progenies derived from homozygous parents. For example, markers may vary in the number of segregating alleles, one or both parents may be heterozygous, markers may be dominant or co-dominant, and usually the linkage phases of marker pairs are unknown. Given these differences, marker pairs provide different amounts of information for the estimation of recombination frequencies and the linkage phases of the markers in the two parents, and usually these have to be estimated simultaneously [37]. Therefore, the maps are constructed independently for maternal and paternal meiosis.

Genetic maps provide important genomic information and allow the exploration of QTL, which can be used to maximize the selection of target traits. The availability of a large number of genetic markers is essential for constructing a useful high-density linkage map and for QTL mapping of genetic traits of interest. The increase in the availability of the genome sequencing data has allowed the construction of genetic linkage maps in a variety of flatfish species, such as the Japanese flounder [9] [38]–[39], turbot [40], Atlantic halibut [41], and half-smooth tongue sole [19]. These maps are invaluable for investigating the genomic organization and identifying the genetic traits of commercial interest. Among these maps, the Japanese flounder constructed by Castaño-Sánchez displayed the densest flatfish linkage map with average intervals of 5.0 cM and 4.4 cM respectively. In this study, we constructed a high-density microsatellite genetic linkage map using 1009 markers in half-smooth tongue sole, a flatfish of great relevance to fisheries and aquaculture. The largest space are 17.3 cM in female and 14.3 cM in male. With average inter-marker distances of 1.83 cM in females and 1.96 cM in males, the new map is at present the densest flatfish linkage map. These markers will serve as an important tool for future comparative map studies and to establish the underlying correspondence with the linkage groups of other closely related species. There were no small (doublet or triplet) linkage groups, indicating that this linkage map is complete. Only 29 of the 1317 markers studied remained unlinked to any other markers. This degree of completeness supports the utility of the genetic map as a reference tool for future genetic analysis in this species.

### Sex-linked Markers

The molecular identification of sex is an important issue for studies involving behavior, ecology, conservation, development and sex determination in many species. The first sex-determining gene was identified in the teleost fish medaka [42]. In recent years, sex-linked markers have been identified in various cultured fish species, including rainbow trout [43], yellowtail [44], Nile Tilapia [45], cichlid [46] and nine-spined stickleback [47]. In the half-smooth tongue sole, a female-specific SCAR marker was proven to be highly associated with female gender and was assigned to the W chromosome [29].

The marked sexual dimorphism in growth which is observed between the male and female half-smooth tongue sole has led to suggestions that the efficiency of the culture systems could be improved by setting up a production system focused on the faster-growing sex. Combining genome sequencing analysis, we identified 159 sex-linked microsatellite marker alleles in this mapping family. Five sex-linked microsatellite markers were confirmed in their association with sex in a large number of individuals selected from different families, suggesting a tight linkage between these microsatellite markers and sex. We were able to map these sex-linked microsatellite markers onto the LG1f region in which female-specific SCAR markers F-382 and F-783 were assigned. Both the male and female maps share the homologous region of LG1 containing the same microsatellite markers, which imply that the LG1 segment is homologous in the females and males, and is an indication of a pseudoautosomal region of the sex chromosome. Further comparison mapping of the W and Z chromosomes should be carried out with these linkage groups. The identification of a sex-linked marker in a general population of half-smooth tongue sole is vital for the further development of mono-sex culture in this species. Sex-linked microsatellite markers are needed for elucidating sex determination mechanism. This is especially important in half-smooth tongue sole because of a large difference in the growth rate between males and females. In addition, sex-linked microsatellite markers have potential other important applications in basic research, such as the identification of sex-related genes and the influence of environmental factors on sex differentiation. The sex-linked microsatellite markers developed in the present study can be used for the molecular identification of genetic sex in tongue sole, and also provide an important tool for screening and isolation of the sex-determining locus and sex manipulation in half-smooth tongue sole.

### Sex-Specific Patterns of Recombination

In this study, the average interval between markers was slightly less for the female map (1.83 cM) than the male map (1.96 cM), suggesting that the recombination rate was slightly higher in males than in females. The recombination ratio between the male and female parents of half-smooth tongue sole was 1.07∶1. Although it was slightly higher in males than females, the ratio was still close to 1∶1. Differences in map length can result from a variation in the number of recombination events in the two parents as well as variations in the number and location of the mapped loci. It is common to find a difference in the recombination ratio between the two sexes in most aquatic species. For instance, the male/female recombination ratios are 1∶8.26 in Atlantic salmon [48], 1∶3.25 in rainbow trout [49], 1∶1.43 in Japanese flounder [9] and 1∶2 in halibut [41].

Despite this being a common phenomenon, the mechanism responsible for the different recombination rates between the genders is still not well understood. Some studies have shown that recombination rate differences are associated with QTL [50]. Selection using linked markers is more efficient when recombination does not occur between the markers and the QTL loci.

### Segregation Distortion

In the mapping family of this study, segregation distortion was observed for 406 markers and the distortion rate was approximately 30.7%, which is lower than the ratio of 33% reported by Liao *et al.* [19]. This suggests that a high ratio of segregation distortion may be a common phenomenon in half-smooth tongue sole. A higher distortion rate has been reported in previous studies, such as 40.5% for Pacific white shrimp [51]. For other marine species, the rate is 26.9% in Pacific Oyster [52], 16% in channel catfish [53] and 16.3% in common carp [54]. The reasons for the distortion of the segregation ratios may be due to the factors such as chromosome loss [55], genetic isolation [56], sampling errors, scoring errors, the progeny population size and amplification of a single-sized fragment derived from several different genomic regions [57]. Additionally, lethal effects caused by a recessive homozygote in the juvenile period may affect distorted segregation [58]–[59].

### Mapping of Sexual and Growth-related Traits

Half-smooth tongue sole (*Cynoglossus semilaevis*) is one of the most economically important marine species in Chinese coastal waters. Information on genetic markers associated with quantitative trait loci (QTL) can be used in breeding programs to identify and select individuals carrying desired traits. In this work, sex-specific linkage maps were used for mapping of sexual and growth-related traits, which provided a full-scale detection of QTL and estimation of the gene effects.

In total, four QTLs associated with growth traits were detected. The additive effects were negative values. To improve the utility of the QTL in MAS and also move toward the positional cloning of candidate genes, fine mapping of the QTL to a more restricted chromosomal region is necessary. Although QTL mapping has been conducted in a few foodfish species, such as rainbow trout [13], salmon [15], European seabass [16], tilapia [17], the guppy [18] and turbot [60], the region is usually longer than 10 cM. In this study, the QTL intervals were 2.4 and 1.6 cM for body weight, and 3.6 and 2.5 cM for body width. Moreover, the four QTL for growth traits clustered on one linkage map (LG4f), which will likely prove to be very useful for improving growth traits by molecular MAS.

In addition, seven sex-related loci were mapped. Half-smooth tongue sole females grow larger and faster than males. Therefore, half-smooth tongue sole has great potential for the production of all-female stock, as well as for studying the mechanisms of both genome evolution and sex determination. Mapping of sex-related locus provide an important tool for screening and isolation of the sex-determining gene and sex manipulation in half-smooth tongue sole.

## Materials and Methods

### Ethics Statement

All the experimental animal programs involved in this study were approved by the Yellow Sea Fisheries Research Institute’s animal care and use committee, and followed the experimental basic principles. A slight fin tissue from the parents and F1 offspring was sheared under MS222 anesthesia, and all efforts were made to minimize suffering.

### Mapping Family

In September 2010, a full-sib family of half-smooth tongue sole was constructed and used for the development of a genetic linkage map. The male parent was selected from a group of fish derived from a wild population. The female parent was selected from a cultured population. Experimental crossing was conducted at the MingBo Aquaculture Company (Yantai, China). Induction of the maturation of broodstock and artificial fertilization of sperm and eggs were carried out as described previously [33]. 120 days post hatching, 300 fry were transferred into big aquarium with recirculation. All conditions in the aquarium were maintained constantly, i.e. water temperature was kept at 20–23°C and fish were fed two times per day at around 6∶00 a.m. and 5∶00 p.m. at satiation. The fish were cultured in such conditions for 6 months before analysis of their genotypes and phenotypes. In June 2011, the F1 offspring had shown apparent disparity in the growth-related characters. Ninety-two individuals from the mapping family were collected randomly. Two growth related traits were evaluated: body weight and body width. *We* represents the body weight in grams and *Wi* represents the length in centimetres. The genomic DNA from the two parents and their progeny was extracted following phenol/chloroform procedures with RNase treatment [61].

### Microsatellite Markers

A total of 3965 half-smooth tongue sole microsatellite markers were tested for segregation across a set of eight progeny individuals. These microsatellite markers were recruited from three sources. (1) The first set of 3000 microsatellite markers was developed from genome sequencing. (2) The second set of 965 microsatellite markers was developed through the construction of microsatellite enriched libraries and EST libraries [19]. (3) The remaining 111 markers were developed from public databases and previous publications [25]–[28].

In addition, 486 microsatellite markers that were developed from other species were used, including 83 barfin flounder SSRs, 78 spotted halibut SSRs, 182 Atlantic halibut SSRs, 96 Japanese flounder SSRs, and 47 Senegal sole and common sole SSRs.

### SCAR Markers

Two female-specific SCAR markers were generated from half-smooth tongue sole in the mapping family (Marker name: F-382, Forward primer: ATTCACTGACCCCTGAGAGC, Reverse primer: AACAACTCACACACGAC AAATG. F-783, Forward primer: GCTGGTGAAGGCTACAATAGG, Reverse primer: TCAGAACACATCACTGCTGC).

### Genes

*SOX9* is one of the genes having a critical role in vertebrate sex determination. Mutations of *SOX9* leading to haploinsufficiency can cause campomelic dysplasia and sex reversal. The microsatellite marker sox9-1 (GQ402461) is derived from the sequence of the *SOX*9 gene in the mapping family. In addition, the microsatellite markers gene072 (Toll-like receptor 9, FJ418072), ghrh-ssr (PACAP-related peptide, FJ608666), gene177 (Ovarian aromatase, EF421177) and gene116 (Myostatin, EF683116) were also genotyped and used for the map construction.

### Genotyping

The primers flanking the microsatellite regions were designed using Primer3 and Primer5 software. All primers were designed for a 57.5°C annealing temperature, a total amplification product size of 100–300 bp and 40–60% GC content. All of the microsatellite markers were used to genotype two parents and six progeny for screening the segregation markers in the mapping population. The microsatellite markers that produced polymorphic fragments were used in the subsequent genotyping of the parents and 92 progeny to construct the linkage maps. Amplifications were performed in an ABI Veriti 96 well thermal cycler, BIO-RAD MyCycler thermal cycler and Fisher Scientific LabServ LS-P96G thermal cycler. The PCR amplifications were carried out under the following conditions: 95°C for 5 min, followed by 32 cycles at 95°C for 30 s, a specific annealing temperature of a specific primer pair for 30 s and 72°C for 30 s, and the final extension was 72°C for 10 min. Amplification reactions were carried out in a 15-µl volume consisting of 10× Taq buffer, 0.5 U Taq polymerase (TIANGEN), 0.6 mM dNTP (+MgCl_2_ 1.5 mM), 0.6 µM of each primer and 10–30 ng template DNA. The final volume was adjusted with sterile distilled water. The PCR products were separated on 8% polyacrylamide gels (PAGE) and visualized by silver staining [62].

### Linkage Analysis

Genetic marker data were scored according to the definition of JoinMap 4.0 [63]. All of the statistical analyses described below were made using the same software using a cross-pollinating (CP) type population, which handles F1 outbreeding population data containing various genotype configurations. Pairwise analyses were performed and markers were sorted in linkage groups at a minimum LOD score of 4.0. The “locus genotype frequency” function calculated the chi-square values for each marker to test for the expected Mendelian segregation ratio. The linkage distances were estimated for each LG assuming the Kosambi mapping function. All weak linkage markers were excluded to ensure a correct marker order. Although distorted segregation markers normally are excluded from linkage analysis, the use of the independent LOD score, one of the grouping parameters provided by JoinMap4.0, allows these markers to be included. This test for independence is not affected by segregation distortion and leads to a less spurious linkage [64].

### Genome Size and Coverage

The estimated genome length (Ge) of the consensus female and male genome was estimated using two different methods. First, Genome Estimation size 1 (Ge1) was calculated by adding 2 s to the length of each genetic linkage group to account for the chromosome ends, where s was the average spacing of the genetic linkage map. The first method estimates s on a genome scale [65]. Genome Estimation Size 2 (Ge2) was calculated by multiplying the length of each genetic linkage group by (m+1)/(m−1), where m was the number of loci in each genetic linkage group. The second method estimates the average spacing for each chromosome independently [66]. The estimated genome size (Ge) for each sex was taken as the average of the two estimates. Observed genome length was taken as the total length (Goa) considering all linkage groups, triplets and doublets [67]. The map coverage, Coa, was calculated as Goa/Ge [68].

### QTL Analyses

QTL analysis was performed with WinQTLCart2.5 software using the composite interval mapping (CIM) method. Unlinked might act as an additional environmental effect that reduces the significance of the estimated marker-trait association. Therefore, CIM includes neighboring markers and uses the remaining background markers as cofactors in order to remove the effects of multiple QTL. While the CIM analysis was conducted separately for each map, the background markers used in these analyses were derived from both maps. Five background markers were employed in CIM analysis. The derived genome-wide threshold value for the three traits was LOD = 2.5. When we analyzed sex-related loci, we considered the sex trait as qualitative trait. The female was “1”, and male was “0”.

## Supporting Information

Table S1 **Characterization of microsatellite markers genotyped in half-smooth tongue sole mapping family.**
(DOC)
