# Correction: Construction of a High-Density Microsatellite Genetic Linkage Map and Mapping of Sexual and Growth-Related Traits in Half-Smooth Tongue Sole (*Cynoglossus semilaevis*)

**DOI:** 10.1371/annotation/b59796a4-fe80-4783-95cf-4a814075d241

**Published:** 2013-05-13

**Authors:** Wentao Song, Yangzhen Li, Yongwei Zhao, Yang Liu, Yuze Niu, Renyi Pang, Guidong Miao, Xiaolin Liao, Changwei Shao, Fengtao Gao, Songlin Chen

Our publication in PLOS ONE focuses on the development of a high-density genetic linkage and QTL mapping for half-smooth tongue sole. This work is closely related to a publication by our group in Current Zoology titled "Construction of a microsatellite-based genetic linkage map for half-smooth tongue sole Cynoglossus semilaevi", which reported a mid-density microsatellite genetic linkage map for the same species:

Song WT, Miao GD, Zhao YW, Niu YZ, Pang RY, et al. (2013) Construction of a microsatellite-based genetic linkage map of half-smooth tongue sole (Cynoglossus semilaevis). Current Zoology 59(1): 99-108.

The article in Current Zoology should have been cited and discussed in the PLOS ONE article and we apologize for this oversight. However, the two papers contain different results. In our paper in PLOS One, we constructed a high-density microsatellite genetic linkage map in half-smooth tongue sole with 1,009 markers with a high resolution (average marker interval of 1.67 cM). In addition, 159 sex-linked microsatellite markers were identified; five sex-linked microsatellite markers were confirmed for their association with sex in a large number of individuals selected from different families; and four growt h-related QTLs were mapped. In contrast, in our publication in Current Zoology, we constructed mid-density genetic linkage maps for female and male half-smooth tongue sole with only 480 and 417 markers, respectively; only two sex-linked AFLP markers were mapped; and no QTLs were mapped.

Because the two projects both deal with linkage mapping analysis, there is similarity in the text of the Introduction and Discussion between the two articles, the authors apologize for this. 

