# Supplementary material for: Construction of a High-Density Microsatellite Genetic Linkage Map and Mapping of Sexual and Growth-Related Traits in Half-Smooth Tongue Sole (Cynoglossus semilaevis)
Source: PLoS One. 2012 Dec 20;7(12):e52097. doi: 10.1371/journal.pone.0052097 (PMC3527371; doi:10.1371/journal.pone.0052097)
Supplement: Table S1 — Characterization of microsatellite markers genotyped in half-smooth tongue sole mapping family. (DOC) [file pone.0052097.s001.doc]

Table S1 Characterization of microsatellite markers genotyped in this mapping family

| Locus | GenBank Accession no. | Primer sequences (5’-3’) | *Tm* (℃) |
| --- | --- | --- | --- |
| scaffold2013_63088 | JN902087 | F: AGGTACGAGGGTCACAGTACGAG  R: GTGGCGTGGTGTAAGGATGCTAT | 57.5 |
| scaffold6362_71677 | JN902088 | F: TCATACGTGGCCACTGATCTG  R: AGAGAAGAGCTAGAGACCCTCG | 57.5 |
| scaffold4762_71394 | JN902089 | F: GCTTGTGAAGAAGTGCAGAT  R: CCTTTGTCTGGTGAAGTTAGC | 57.5 |
| scaffold676_31790 | JN902090 | F: GATTTCAACCATGTCACGTC  R: CAGCATTCAATGACAGCTC | 57.5 |
| scaffold1278_49680 | JN902091 | F: TGGTAGCAGGTCAACACCCAG  R: GTAAGGGCAGTGGTGACCGA | 57.5 |
| scaffold343_16599 | JN902092 | F: CAACAGATGAAACGACCACT  R: TCTGCTGAGAGAGGCATTTA | 57.5 |
| scaffold370_18039 | JN902093 | F: CTCAGTACCAACTGCTGCTC  R: TGGACCCCAAATGGTCAGTA | 57.5 |
| scaffold3034_69811 | JN902094 | F: CAACACGCTGCATAACACTG  R: GCTGATCCTTTTGCCTTTACCTTT | 57.5 |
| scaffold1596_56445 | JN902095 | F: GTCTCAGGCGGATGATGAGT  R: GCCCTACTTCCTGCTCACA | 57.5 |
| scaffold2735_68898 | JN902096 | F: TAGGCTGTTGCCTGACTCCT  R: GGCCAACACACCTCAGTCAT | 57.5 |
| scaffold2860_69245 | JN902097 | F: GTTGACAGGCATGGTCAGAA  R: CTGGTGGTCGAGGGAATATG | 57.5 |
| scaffold65_4377 | JN902098 | F: CTTGAGCAAACACAAGCAACTAG  R: ATCTATCGGAGTGGCCTATGA | 57.5 |
| scaffold1490_54527 | JN902099 | F: TTCACACCGAAGCAGTTTTG  R: CCCTTCTGGTTCTGCTTTTG | 57.5 |
| scaffold6_131 | JN902100 | F:ACAGAGGGAAATAAAACCTGACT  R:ATGCATTTGGAGTAGGGTACA | 57.5 |
| scaffold1442_54378 | JN902101 | F:GCTCGTTGCCAAAGGGACAT  R:AAGTGGACTGGGACGACTGAA | 57.5 |
| scaffold550_27277 | JN902102 | F:TGCAGCAGCTGGAACTGTTTA  R:GCCTGCCAGAAAATCAATAGG | 57.5 |
| scaffold1829_59722 | JN902103 | F:GGGAAGTGACTTTGCAGATGAG  R:AGTGCCAACACCAACGAAATAC | 57.5 |
| scaffold5567_71583 | JN902104 | F:CATCATGTTAGACACCCTTGCA  R:TGTGCCTCTGTTCATCAAACC | 57.5 |
| scaffold1424_53261 | JN902105 | F:GCTGGGCACGAACATAACTG  R:ACGCACAACACGATTAGGATGA | 57.5 |
| scaffold371_18635 | JN902106 | F:CGTCCTGACCTTCAGTCCAT  R:ACTGTGGTCGTGTCACTCC | 57.5 |
| scaffold1425_53252 | JN902107 | F:GCACTCACAGGGAACTCTCAG  R:AAGTGAAATTCTGAAGCGACTGCT | 57.5 |
| scaffold295_15370 | JN902108 | F:AAAGTGGCTTTCAGTGAGTGGA  R:GGTTGGGAAAGTCCTCTTCGA | 57.5 |
| scaffold855_36363 | JN902109 | F:ACACACCTGACGGAACCAG  R:GAGGCATGTGGAAACAGTAACGA | 57.5 |
| scaffold3741_70924 | JN902110 | F:TGTACTCATCCTCCCATGCAC  R:GGAGACCAGGACTGAAGGAC | 57.5 |
| scaffold504_23623 | JN902111 | F:ACAGCACTGATGGTGACACAG  R:TGTCTTGGTCCAAACCTCATCC | 57.5 |
| scaffold56_2355 | JN902112 | F:GGAGCATTATCCCCTGGAGTT  R:GACATCTCATCACAGCCTGGT | 57.5 |
| scaffold908_38904 | JN902113 | F:GAGAGACTCGCAGGAAGTCAG  R:TGCCCTTTCAGCTGTTTGCTTA | 57.5 |
| scaffold2063_63702 | JN902114 | F:GCTACAAAGGACACCCTGC  R:CGCAGACGTCCTCCTTTTCAT | 57.5 |
| scaffold362_17927 | JN902115 | F:CGGAAAGGTGGAAGTCGGATT  R:CGACCCTCCAGGTTAAAGATCAG | 57.5 |
| scaffold362_17931 | JN902116 | F:CTCTCGAGCTCCTTTGCAATTCA  R:TTCACCTCAGTGAGCAGACAC | 57.5 |
| scaffold1203_47625 | JN902117 | F:AGATCAATCCCTATCCGTGCTG  R:GCACAGAAACCCCTGCTTTTAC | 57.5 |
| scaffold3620_70747 | JN902118 | F:GAGTGGGAAGACTGGTCCAAAA  R:CTCCACTTTCACTGGCCTTCT | 57.5 |
| scaffold1044_43397 | JN902119 | F:AGTCTGCACTGTTCTCTACAGCT  R:TATACACAGGCCCACACAACATG | 57.5 |
| scaffold2632_68353 | JN902120 | F:TGACGTGACCTCCAATTCCATTTC  R:TCGTCTCTTTACGTGACCCTGT | 57.5 |
| scaffold1964_62378 | JN902121 | F:TCTGGTCTCTCCTCACAGCAA  R:TCACAACAGATCCACACGTGAC | 57.5 |
| scaffold1658_57964 | JN902122 | F:GTTCTCGCATATAATCGTGCTTCGT  R:CCTCCTGATATCACCGCCTATC | 57.5 |
| scaffold990_41735 | JN902123 | F:GCTTTATCCACGGAACAGTCCA  R:ACCGACACTGATAAGGGCC | 57.5 |
| scaffold256_49 | JN902124 | F:CCCTCATAACTAGACACATGCCAAG  R:TCATTTCAGGTTTTGGAGCCATCG | 57.5 |
| scaffold2935_941 | JN902125 | F:CCACAAGAAAGCCTCCTGACA  R:CATGTCGACTCTTTACTGACCCC | 57.5 |
| scaffold2984_949 | JN902126 | F:CAAAGGGAACTCCCAAGGGAT  R:GTTTGAAGCTGCCACAGAACAAG | 57.5 |
| scaffold2999_952 | JN902127 | F:ACTAATCTTACCTTGGTGTGGTTTGCT  R:GTCCTTTCAAGACTCAGGCAACA | 57.5 |
| scaffold3051_955 | JN902128 | F:CAATGATGCGTGTCCTGTTGC  R:TCTGACACTGATGTGGCAGTCA | 57.5 |
| scaffold3067_958 | JN902129 | F:CTGTGTTCAGCCTCACCATCAT  R:GACACGCAGAAAAGAGCACTCA | 57.5 |
| scaffold3391_1010 | JN902130 | F:GTCACAGTGCATCAGATGGCT  R:CGTAATGTCAGTGTGGTGCAGAA | 57.5 |
| scaffold3571_1038 | JN902131 | F:CAGAGCTGCTTTAGCTGCTTGA  R:GCTACAGCAAACATTACTGACGCA | 57.5 |
| scaffold3928_1054 | JN902132 | F:GCGAAGAACAGAGTTTATCTGG  R:GTACACGAGCCAGTATTCAGTA | 57.5 |
| scaffold101_17 | JN902133 | F:AGTCCTGCATTTGGGTCCTG  R:AAGTCTGGGTGTCCCTGC | 57.5 |
| scaffold1024_286 | JN902134 | F:ACTCTGTGGAAAAAGGCTTGTAATCG  R:TCTTTCAAACACACAGTCCTCGTCA | 57.5 |
| scaffold1030_289 | JN902135 | F:GAAAACCTCCACAGGGTGTGT  R:CCTACAAGAACAAATGTCCCCACAA | 57.5 |
| scaffold1038_293 | JN902136 | F:GGGGACCCCTGATCTATACAGT  R:TGCTGAAAAAGTGGTCGAGAAGC | 57.5 |
| scaffold1103_308 | JN902137 | F:GCATTCCTGTGAAGAATCCAAGCAT  R:TCTCCCCTTGCAAGTAAAAGATCCA | 57.5 |
| scaffold1111_312 | JN902138 | F:TGTGGAGGCAACGTGTCATC  R:TTGTCCTCGAACAAATCAAAGTCCG | 57.5 |
| scaffold1113_315 | JN902139 | F:GTGGCCCTCCTTGTTATCACATT  R:AGCTCTGTGGATGACAGCAAAAC | 57.5 |
| scaffold1128_343 | JN902140 | F:GAGGCCGACAGGATCGTAC  R:TACGACGTACTCCGGTGGTTTT | 57.5 |
| scaffold1169_362 | JN902141 | F:GGGGGAGACTTTGTGACTATTGG  R:GGTGGAACACAGAAGCACAGAA | 57.5 |
| scaffold1206_373 | JN902142 | F:TTGCACCCTAACCCTAACACAAC  R:ACAGGATGAAATGACTGAAATAGTGCCA | 57.5 |
| scaffold1220_378 | JN902143 | F:TGAGTGCAAATCCCAACAAAGATGAC  R:TCCTGGGCTACATTGACCCTAA | 57.5 |
| scaffold1235_383 | JN902144 | F:CAGAACGCCATGTTTGAGTAATGTGT  R:TTGTTTGCAGAGGAGAACCTTCC | 57.5 |
| scaffold1246_388 | JN902145 | F:CTGACGACTCGTTTGAAGGCAA  R:GAGGCTTAAATGTAGCAGCCTCTC | 57.5 |
| scaffold1305_404 | JN902146 | F:TTTGGAGACTTGAGAGGGGTGA  R:TTGCGGTAGGCTATATGCAAACC | 57.5 |
| scaffold1332_411 | JN902147 | F:CAGTGTGTGTTTATTTAAAGGTTAGTTGGGG  R:GCACTAGTTGGACTAGTGTTGTACTCA | 57.5 |
| scaffold1339_414 | JN902148 | F:CCTGAAACATGTGCTGGGAAGAAA  R:CTTCACCAACCTCCTCCTGAG | 57.5 |
| scaffold1354_430 | JN902149 | F:AGGCAATGTCTCTGTCATTGGTG  R:CGTGACAGTTCTTTGACACCTGAG | 57.5 |
| scaffold1358_427 | JN902150 | F:GGACGTCCCTACATTTCCATTCG  R:AAGATGCAAAGCGGGACTACAC | 57.5 |
| scaffold1366_424 | JN902151 | F:ACCAGTCCGTAGGTCATTTGGT  R:TGGGAGAAGAGCTGGAGGAA | 57.5 |
| scaffold1392_450 | JN902152 | F:ACTTCCACCTAGCCAGTTCCA  R:AACTCCCGAGGGATGAATAAAGTTCTT | 57.5 |
| scaffold1423_459 | JN902153 | F:GCATCAAGTGAGTAGCGCTCAA  R:AGTATCTCACCCTCTGTGAAACTGTATG | 57.5 |
| scaffold1447_469 | JN902154 | F:CTGCACGGTACATCACCTGTC  R:GGATGGATGGCGGGATAGAATG | 57.5 |
| scaffold1467_473 | JN902155 | F:CCACCACGAAAAGGGAAAATCTGT  R:CGTTGCTTTACTGGACGGACA | 57.5 |
| scaffold1500_508 | JN902156 | F:CACTTCCACCGTCATCCTCAC  R:TGAAGTTGCTGATCCAAACACCC | 57.5 |
| scaffold1503_484 | JN902157 | F:TGACCATGTGGTACAATGGA  R:AGAGCTGGAGGAAGTGTC | 57.5 |
| scaffold1545_522 | JN902158 | F:TGTTCCTGCTCAGACTGTTGC  R:TCCCTCCGTGTCCTGATTGATT | 57.5 |
| scaffold1573_527 | JN902159 | F:CTGGCATTTGTACAAGGGTGTGT  R:GCAAGGACAGGTATAACTATACTGGGT | 57.5 |
| scaffold1611_534 | JN902160 | F:GGGTCCTGGCATGAAGACAT  R:AAGTGTCGAGGGAGAGGGAA | 57.5 |
| scaffold1612_540 | JN902161 | F:GTATTCTCCCTTCACGGCCTC  R:TGTTTCTGGGGAAGAAAAGCAATCAG | 57.5 |
| scaffold1614_544 | JN902162 | F:AGTGTGTGGAAATGCAAACCTCAG  R:CAGGCCTGACTATACAAGGACATCA | 57.5 |
| scaffold1653_550 | JN902163 | F:AAGTGTCGAGGGAGAGGGAA  R:TGCACCTAACAACAGTGATTGATCG | 57.5 |
| scaffold1657_558 | JN902164 | F:TCACTGCACTAAACGGCATCAAG  R:GGTGGAAGGCAATATGCTGAACA | 57.5 |
| scaffold1658_561 | JN902165 | F:CAAGTCCAAATCAGCTGCAGAAGA  R:GTTGGCACAAGTCATGCAGTACA | 57.5 |
| scaffold1676_567 | JN902166 | F:TGATGGACAGTGTGCTCCGTAA  R:AAGTGTCGAGGGAGAGGGAA | 57.5 |
| scaffold1905_607 | JN902167 | F:GGGGTGCTGTCATAGGGTATTG  R:CACCGTTCACCTGATTTGGATGT | 57.5 |
| scaffold1916_609 | JN902168 | F:CGACTACGTCACTCGGCTG  R:AGCCCAGATATACGTGATATACGCTAGAT | 57.5 |
| scaffold1923_610 | JN902169 | F:TGGGGTATGAACTAACAGCAT  R:GAACGATGTGTTCACAGTTACATT | 57.5 |
| scaffold1938_613 | JN902170 | F:ATAGTCCATGGGGAGTCCTGTC  R:GTAAATTCCTCCAGGTCAAGGTGAC | 57.5 |
| scaffold1941_615 | JN902171 | F:CATCACCCCAATTGATGTGATGAGTAAC  R:TGCACGCACTAGGACAAGAATC | 57.5 |
| scaffold1985_624 | JN902172 | F:CAACATAGTGCAAGATGACACAA  R:AGATCACAAACAGTGAGAGCT | 57.5 |
| scaffold1991_626 | JN902173 | F:CAGCAGAGCTTTTGGGGACTTTA  R:CTTGCTGTTGTGTACTGTGAATGGT | 57.5 |
| scaffold1993_631 | JN902174 | F:CTCAGTGGGGGTGGTTTAAAGTT  R:CTATAAGTGAGTGTGTGGGGGTTG | 57.5 |
| scaffold2028_634 | JN902175 | F:ACTGCTGCTGGAGCCTTAATTTC  R:ACTGCTGTTTCTGTCCCCTGTA | 57.5 |
| scaffold2032_643 | JN902176 | F:TCTCTTCCCGCATCTTGTCCT  R:TGCACTCTAAACACAGCAGTCTCA | 57.5 |
| scaffold2035_641 | JN902177 | F:GTGGCATACCGTGCAGTGAAATA  R:GCTCTCACTGACCTACCCAC | 57.5 |
| scaffold2066_646 | JN902178 | F:AGGTTTGTTGACTCAATGGC  R:CATTTTCTATAGTGAGTGTATGACAGC | 57.5 |
| scaffold2080_648 | JN902179 | F:ATGTCTTTCAGGTGGCGACTTC  R:GCTTCTTATGTTGCGATTCAAGTCAGTT | 57.5 |
| scaffold2081_663 | JN902180 | F:TGTCAGGACGTTGTGATGCTG  R;CCTGTCCCCCCTTGTCAATT | 57.5 |
| scaffold2133_705 | JN902181 | F：GGTGTGGTAAGGCTCCAGAAG  R:CTGGCGATTCCCACTCTTAGATG | 57.5 |
| scaffold2148_678 | JN902182 | F:TTTTGCTGCTGAACACACTCACA  R:TACCATTTGTTGCACTGTGCTGt | 57.5 |
| scaffold2158_690 | JN902183 | F:CCAGCATTCATCAGGAAAAAGGC  R:ACAGTGGTTACGTTGGCGAAATT | 57.5 |
| scaffold2164_693 | JN902184 | F:TGCAGGAACTCTCCTCCAAAAAC  R:ATTTTAAGCTGCAGCCACACACT | 57.5 |
| scaffold2169_697 | JN902185 | F:TCCACCAGAGCAGCATAAAACAG  R:CATTATCAGCCAGGAAACATGAGCA | 57.5 |
| scaffold2360_734 | JN902186 | F:TCCGCCCACCACTAACTTGTAT  R:ACGCAAATCCTGTCAAGAATAACTTCC | 57.5 |
| scaffold2431_777 | JN902187 | F:AGTGGAGGAAGGGAAAATTGGTG  R:CAACCTGCCTCTCGTTTTCAGT | 57.5 |
| scaffold2440_778 | JN902188 | F:AACCCATCACCAAAGTCAGCAG  R:GGAAGGCTTAAGGTTCGAGTCC | 57.5 |
| scaffold2453_780 | JN902189 | F:CTCAGGTGTGCCCAAACTCTTT  R:CAGTGTGTCTGTTTTTCCCCCA | 57.5 |
| scaffold2484_785 | JN902190 | F:CCAGCACTTCTCACGGTAAACAT  R:ACTGCCACTGGCTACTGTACT | 57.5 |
| scaffold2527_791 | JN902191 | F:TATTGGAAGCTGCACACACCTC  R:GTTGGCACAAGTCATGCAGTACA | 57.5 |
| scaffold2532_794 | JN902192 | F:TTTGTGCCAAGGATGtcttCTGAC  R:AGACTGTTGAGCAGGAAATGGAGA | 57.5 |
| scaffold2565_799 | JN902193 | F:TGCAAGGTCAGATACAATAGTCAA  R:ATAATAACCCCAGTTGTTTGCC | 57.5 |
| scaffold2566_800 | JN902194 | F:CTCTCAACCCCCATTTATGACCATG  R:CCCCCAGAAGAGCTAGAGGA | 57.5 |
| scaffold259_54 | JN902195 | F:CTGGTTCACAACCATGACGTCTAC  R:GGACAGGCCTGGTATTTATGAGTG | 57.5 |
| scaffold2607_804 | JN902196 | F:GCCCATTGTTCACTTTGGTGATG  R:TTGATATGGGCTGAAAATCGGTGTG | 57.5 |
| scaffold2618_811 | JN902197 | F:GGGTGCCCAAACTTTTTCATACC  R:CATTTGTCCTATGGACTGTGGACTG | 57.5 |
| scaffold267_55 | JN902198 | F:CCAGGTATCCAGCTGTTTGCT  R:GATCTACACTCAGGGCTGCAC | 57.5 |
| scaffold2719_837 | JN902199 | F:CCCCTGCTGTATCCCATCATATACT  R:GCAGGATGTTGCTAACGGAACT | 57.5 |
| scaffold2722_838 | JN902200 | F:CCCCTAGAAGAGCTGGAGGA  R:GCCATGTCTACCAGGTTTTACTGG | 57.5 |
| scaffold2744_845 | JN902201 | F:GCTGCTGTCTGTTGTTGCAG  R:GTCGAGGGAGAGGGAAGTCT | 57.5 |
| scaffold2746_846 | JN902202 | F:GATGGGCTTTACAGATGGGATGG  R:AAATGGTTGCACTTCCTGGTTCAT | 57.5 |
| scaffold2905_937 | JN902203 | F:AGTTGGCACAAGTCATGCAGTAC  R:TCCAATGAGAGACCAGGAGGTT | 57.5 |
| scaffold2950_942 | JN902204 | F:CCATGTTATCCATGCTTCTGAATTCGA  R:ATGGCCGTCACACTACCTG | 57.5 |
| scaffold2957_945 | JN902205 | F:ACTCGGTCCCTAATCTGTGTGTT  R:GCAGAAGGAAATCCCAGTCAGAG | 57.5 |
| scaffold3003_954 | JN902206 | F:AATCTAACAGCAAAGGCTGTGATTC  R:GGTGTAGGCTGCAGAATTGTAG | 57.5 |
| scaffold3032_956 | JN902207 | F:CAAATCAACTAGTCTGGCTGTCCAG  R:CACAGCAGCATCCCTTATTTTGC | 57.5 |
| scaffold3064_962 | JN902208 | F:ACCAGGACCAGGATGGACT  R:CGGTTGTACGGAGAGGGATTC | 57.5 |
| scaffold3068_959 | JN902209 | F:CAGAAGAGCTAGAGGAAGTGTCGA  R:AGCATAGTGGTTTGAAATTAGCAGGG | 57.5 |
| scaffold3080_963 | JN902210 | F:CTCCGGCATGCTATAGAGATAAGCA  R:TCAGTGTCCTTGGGTGACTTGA | 57.5 |
| scaffold321_65 | JN902211 | F:GTGCTGAACAAGACAAGTGCAAAG  R:CATGTGACCCAGAAGAAGACTGATTG | 57.5 |
| scaffold3212_984 | JN902212 | F:GAGTGTCTGATATGCACAGTCAGC  R:AGGTTGTAGCCCATCAACGG | 57.5 |
| scaffold3286_996 | JN902213 | F:AGAAGGCCATAAAAGCATTACGATGG  R:CCAAGAAAGATGCCCACTCCA | 57.5 |
| scaffold3357_1000 | JN902214 | F:CCAGGGACAGTGGAGTAGAGA  R:CAGCTGATATTCACCAGCGTCTG | 57.5 |
| scaffold3360_1002 | JN902215 | F:GCATGTGTGCTTATCAACGCAG  R:ACAAGCAGTAGGTACATGTTAGCAACA | 57.5 |
| scaffold3366_1003 | JN902216 | F:CGACAGCTACTGTAAACAAGCCTC  R:GGTGTATCTAATAAAGTGGCCGGTG | 57.5 |
| scaffold3383_1009 | JN902217 | F:GGCTCTCGTCGTTCACTTCAAATAG  R:GCAATCAGTTTGGTCTGTTGTCGA | 57.5 |
| scaffold3405_1014 | JN902218 | F:GGAAGGTTTGTCAGCTGCACT  R:GTCTTTCCTCCTCCCCTCAGA | 57.5 |
| scaffold3424_1020 | JN902219 | F:GCGTTTCCCTGTGGTTGATG  R:AGTTGCCAGTGTGTGTTTCCA | 57.5 |
| scaffold3487_1025 | JN902220 | F:CTATGAAAGGCTCGCAAACACAGA  R:GCTTGGCCTCACGTTACTGAAT | 57.5 |
| scaffold3501_1026 | JN902221 | F:CCGGGCTTGAAATGAGAAAGCTA  R:ATTTTGTGCTGCCAGTGTGTCT | 57.5 |
| scaffold359_74 | JN902222 | F:TTGCCCCAGAGAAAGGAACC  R:GTTGTCTTACCACGGTGCAATGT | 57.5 |
| scaffold3644_1039 | JN902223 | F:CACCCCTGATCATTTTCAAGATT  R:CAGAAGAGCTGGAGGAAGT | 57.5 |
| scaffold3726_1044 | JN902224 | F:GCATCACTGTGGCCCAATG  R:TCCTCCTGTTGTCACTGAGCA | 57.5 |
| scaffold3815_1048 | JN902225 | F:TTACCCTGGCTGACGGTCTAA  R:ACAACATCCCAGCTGACATAACG | 57.5 |
| scaffold386_75 | JN902226 | F:TCTGGGTGTCCCTGTTCAGA  R:CTGCCATTTGGCTACGACTATTTAGTG | 57.5 |
| scaffold3895_1051 | JN902227 | F:TAGTGGGGGTCAACCAGAGTT  R:AGATGTTGGGGTTCGCAGG | 57.5 |
| scaffold3922_1053 | JN902228 | F:ATGAATGAGAGGAGAACAGGGGAA  R:CCATGACCTGGATGACTGAGAATC | 57.5 |
| scaffold393_77 | JN902229 | F:AGGGGACCTCTTCAGTGCT  R:CGATAGCAGGATCTTAATGGGCC | 57.5 |
| scaffold3982_1057 | JN902230 | F:TTGGACTGAGCTGTTCCT  R:AATGCATGTAGCTGTATGGC | 57.5 |
| scaffold402_79 | JN902231 | F:CCTCTGTATGTTCTGTACATGTGAAGGT  R:AAACCTTTCTGATGAGGACCACAA | 57.5 |
| scaffold4113_1070 | JN902232 | F:ACGGAGTAGTCTAGTAATCAGAC  R:CAATGGGTGTAATGCAACAG | 57.5 |
| scaffold4119_1071 | JN902233 | F:GAACACTTAATGGGGCAGAATTGACA  R:GGCACCACTGTCAGAGATTGTAG | 57.5 |
| scaffold4172_1073 | JN902234 | F:AGATGGCGGAATGTCTGTTAGAAGT  R:AGCACACACTGGAAGACACAG | 57.5 |
| scaffold4195_1075 | JN902235 | F:CAGAAGAGCTAGAGGAAGTGTCGA  R:TGAAGAGAGCACCAGTTGCC | 57.5 |
| scaffold422_81 | JN902236 | F:CTGTCAGTGCTTTTGCATTGTGTC  R:CCGCAATTCCAGCCTAACAAAC | 57.5 |
| scaffold4220_1077 | JN902237 | F:GACACAAACACACACCTGTTA  R:GACCGCATAGACAGCTAAAAC | 57.5 |
| scaffold4254_1081 | JN902238 | F:CTCACTGCCCAAAATGCTCTTTCT  R:ACAGGTGCGAGTGATGAAGC | 57.5 |
| scaffold4278_1084 | JN902239 | F:TGTCCAGCGACACCTATT  R:TTTGCAGGTATGTGTTGTATGG | 57.5 |
| scaffold4321_1086 | JN902240 | F:AATGATCACGGGTGCCCAAA  R:TGCAAGGCTCTACAACAGGAAC | 57.5 |
| scaffold4466_1096 | JN902241 | F:CCATCCGGTTCTGGAGAGATTTTC  R:CTCATTCATACTGCCCTCCCTCT | 57.5 |
| scaffold4665_1103 | JN902242 | F:CCAACTGGTGACCAAAAACAGAGA  R:GGAAAACAATGATTCGCTTTACCCCT | 57.5 |
| scaffold484_90 | JN902243 | F:GCGTGAGGAGATGTTTGTCTGT  R:ACTCTGTGGTTTGTAGCATGGTGA | 57.5 |
| scaffold513_107 | JN902244 | F:CACAGGCATTTCGAAACAAAACCC  R:ATGGTTACCGCCTGAAAAAGAAGAC | 57.5 |
| scaffold522_110 | JN902245 | F:ACCATCTCAATCTGGATTCTCTAG  R:CAAAGGGTGAACATCATGACA | 57.5 |
| scaffold543_118 | JN902246 | F:GGCCGACCGATTATATGCTGTTC  R:GGCGGTTAATACCTTCCAAAGGAC | 57.5 |
| scaffold548_121 | JN902247 | F:AACTCTGGCTCAGGGTCAAAC  R:GGTCGATGTTCTGAGGTCGTC | 57.5 |
| scaffold552_122 | JN902248 | F:AAAACCTTGAAGCAGACGCAAGT  R:AAGTGTCTGGGGAGAGGGAA | 57.5 |
| scaffold56_4 | JN902249 | F:GGGGCATGAATTCACTCTTATCCC  R:GGGGGAGTTGACCATCACATC | 57.5 |
| scaffold571_123 | JN902250 | F:TCCACTCACCTCTCTTTCTCGTG  R:CATGACCCCCTCACAACTAGG | 57.5 |
| scaffold6_1 | JN902251 | F:TCACCAAATGCTCTGGTCAATGTC  R:CAGGCTTTCAGCAAGACCTCAA | 57.5 |
| scaffold605_142 | JN902252 | F:GCCCCTAACGGATAAGCAGATG  R:CGAAGAAACGAGCGGGAATTTAGATG | 57.5 |
| scaffold670_169 | JN902253 | F:CAGAACATGGCAGGAACTGTGT  R:AGTGTTCAGTCACTGGGCATTC | 57.5 |
| scaffold693_174 | JN902254 | F:CCCATTTGTCAGCGTCAATAGTTCA  R:GAATCCACCCAACTAGACACCAG | 57.5 |
| scaffold718_176 | JN902255 | F:CTGCCAAATGGGTCCTAAGAGAC  R:TGCTTTGCCCGTTCCTCATAAAC | 57.5 |
| scaffold757_179 | JN902256 | F:TGAAGAAGGAAGGCAGAGTTGGA  R:CAGAGCACAGCAATGTTCACCT | 57.5 |
| scaffold764_184 | JN902257 | F;AGGTCGCATCTAGTGATTCCAAATGT  R:GACGAGGTTTGAACCCAGAGAAG | 57.5 |
| scaffold804_188 | JN902258 | F:AGCAACTTGTTCCGTTCAGAGG  R:TTCCAAGTGTAGCTGGATCGTGA | 57.5 |
| scaffold813_200 | JN902259 | F:AGGCTATGTGCATCCTTTGTTTACTG  R:CACCAAGTGAGTAGCGCTCAAA | 57.5 |
| scaffold83_6 | JN902260 | F:CCGGACAGCATACTCCCTCTAA  R:TCATATGGGACCTCAAGGTTGGT | 57.5 |
| scaffold868_211 | JN902261 | F:GATATGCATGAcccaggacactaga  R:TATGAACGCAAACGCAGCTTAGTAC | 57.5 |
| scaffold869_212 | JN902262 | F:GGGAGGTGTGTGTGTGTTTGA  R:AAGGGCCTATGGGATTCAGC | 57.5 |
| scaffold885_215 | JN902263 | F:CTCCAGGGCTAGGATGTGC  R:TGGAGCAGATTAGGAGGAGGG | 57.5 |
| scaffold887_217 | JN902264 | F:CAGACTGTTCAGTCGCGGTAG  R:GTAACTGATAAGCGACAGGATGAGC | 57.5 |
| scaffold899_221 | JN902265 | F:GCTAACAACCTAACCTAAACCAACCTAAC  R:GCTGCCCTGTCCCTTACATATTTTT | 57.5 |
| scaffold914_225 | JN902266 | F:TCCTCAACCTGTGTTTGAATGCTTC  R:GTGTCCTTGAGTGACCTGAAAGG | 57.5 |
| scaffold93_13 | JN902267 | F:ACCCAACAGTTTTTGAGTCCTGATTC  R:TTAGATGAGGCAAGGGATTGGC | 57.5 |
| scaffold1049_297 | JN902268 | F:CGCATTTTTGTGCACACCATTTGT  R:GGTCATGTTGCACAGTGCTTC | 57.5 |
| scaffold1185_367 | JN902269 | F:AAGTGAACCAAATATTCTGGAAGGTGAGT  R:TATGCCTGTGCTACACGTTTGAC | 57.5 |
| scaffold1189_370 | JN902270 | F:TGCTCGTGAACGTTCTATTTGTGG  R:GCATCGAAATGACGATCGAAATGTGT | 57.5 |
| scaffold1283_399 | JN902271 | F:GGTTCTCAGTCACCCAGGTC  R:GAAAGCACCTGGACAAAGTTCTGA | 57.5 |
| scaffold1590_532 | JN902272 | F:CCCAAAGATTACCCCACAAAGACAA  R:CTGTAACAAGTGAATTTCCCCGGT | 57.5 |
| scaffold1890_606 | JN902273 | F:CATAAGGCTCCTCGTCAACAATGTATG  R:AGAGGCTCATTGAAGTTGACCTGA | 57.5 |
| scaffold1951_619 | JN902274 | F:GGGCTCCAGGTACAAGTTTGT  R:CACACAGGGAAGCTCTGCT | 57.5 |
| scaffold205_41 | JN902275 | F:CGTCCAATCTGCAAAGTGCC  R:ATGTCCCTTCAGCCAGGACTAAT | 57.5 |
| scaffold2194_710 | JN902276 | F:ACAGGCTTTCATAGGGTGTCCT  R:GATTCTGGGTGCAATACCTCCTG | 57.5 |
| scaffold2195_702 | JN902277 | F:TGGCTCGATGGAATGAACTGACT  R:CATGCTCCCCCTCTCATTAACATC | 57.5 |
| scaffold2211_718 | JN902278 | F:CGACAACGACGGTCCAATCA  R:GCGGGTTTGGTCTGACATTCT | 57.5 |
| scaffold2322_727 | JN902279 | F:TTCTATCTGCGCTCCCAAAGC  R:AATTGGTAGGCAAACACACAGCA | 57.5 |
| scaffold2345_732 | JN902280 | F:CACCTGATGATCCCTCGAATGG  R:AGGGACAGGAATTTGTGAAGCC | 57.5 |
| scaffold2372_736 | JN902281 | F:GCCTCTGTTCCTATGCCTCAAG  R:TGGACAACTAGGAGCgatgga | 57.5 |
| scaffold2491_788 | JN902282 | F:ACTGAGGAACAACACCACCAC  R:GTGCGATACATCTGCTGTGCA | 57.5 |
| scaffold110_5695 | JN902283 | F:TGGCATAAACAACACTGCCTCT  R:ATGTTTATCACATGGTCTGGGCTT | 57.5 |
| Scaffold077431 | JN902284 | F:TGACTCAGTCTTGAACCTCACC  R:CAGGGTACACCACACGATATGT | 57.5 |
| scaffold467_24010 | JN902285 | F:TCACATGTACCACATGTGTCGAT  R:CGCATGTTTTTCAACATGTCATCC | 57.5 |
| scaffold4497_71352 | JN902286 | F:ACCTGTCGACAAAACACGAACA  R:AGCCGCTAGCTTCATAAACCAA | 57.5 |
| scaffold2670_815 | JN902287 | F:TTTCCTGGTAAAAGACCTGGCAATAAAC  R:TGCTGTGCTGTGTTTGACTTGA | 57.5 |
| Scaffold000357 | JN902288 | F:ACAGCACAAATGACTGATGTTGG  R:ATTCAGTTTGGGAATCGCAGC | 57.5 |
| scaffold2821_69157 | JN902289 | F:TCTCCACCAATAGGAGCAATGTG  R:TGAATCCCCTAGTGATTCAGGGA | 57.5 |
| scaffold1033_290 | JN902290 | F:ACACGCCGACACAGATGATAGT  R:CGCAGGTGCAATTCGAGTAATCA | 57.5 |
| scaffold2088_649 | JN902291 | F:GTGATGGAGACGGGATGTCAG  R:GTATGAGTTCCATTACCGAAGCAGAAC | 57.5 |
| scaffold37_983 | JN902292 | F:ATGAGGTGAGGCAGTGAGG  R:GCGGCTCTTATAGCCCTTCT | 57.5 |
| Scaffold077066 | JN902293 | F:GTTAACCTCAGGGGTCAAGGT  R:CACAGTGGGGTGAAGAGTCA | 57.5 |
| scaffold170_6408 | JN902294 | F:AGGCCTGGTGGAGTTCTAC  R:AACAAGCAGGGGTGGTACA | 57.5 |
| scaffold700_21074 | JN902295 | F:TCTGAGAAACATCGCCACTTGT  R:GAGCATCTCTGACTGGAGGT | 57.5 |
| Scaffold077108 | JN902296 | F:AAGCTGAAGCCTCTGTGGAT  R:ATGTGCCTCTGTCTCCTCC | 57.5 |
| scaffold076365 | JN902297 | F:CCCCAGAGCAGGTTCAATC  R:GCGCTAACAGGTGTTCAAACA | 57.5 |
| scaffold3490_70561 | JN902298 | F:GTACTGAGCGTCCGACACATA  R:CATCCAGAAACTACAGCAGGAAC | 57.5 |
| scaffold32_739 | JN902299 | F:GAGGAGGACGTTGGTCACT  R:CATGTGGGTGAGCATCAAGTC | 57.5 |
| scaffold578_27322 | JN902300 | F:AGCTCAGATCAACTGATCATGTG  R:CTGACCGTCATGAGTTGGT | 57.5 |
| scaffold578_27319 | JN902301 | F:ACTCTTCCTGGGTCCAAAGT  R:CTCTACATGCAATAGGGATGCA | 57.5 |
| scaffold1021_42142 | JN902302 | F:CCTGAGGGAGTCACTTCAGATTAC  R:TAAGTGGCAGCAGTCGGTTAG | 57.5 |
| scaffold360_12245 | JN902303 | F;TGCACAAACAAGGTGAGAGACA  R;CCCTGGATAAGGCATGAACTGA | 57.5 |
| scaffold2700_68530 | JN902304 | F:GCTCGGACGATAATCAACCACT  R:GTCATGCCTCTGCAATGTTCTC | 57.5 |
| scaffold3044_69798 | JN902305 | F:GCCGGAGCTCATCTGATCTTA  R:CAAACAGCCAATCAGTCCACA | 57.5 |
| scaffold152_7435 | JN902306 | F:ACAGCCCAGATGTCGTGTCTT  R:CCTCCCTTCTCTCTGGACCT | 57.5 |
| scaffold888_37657 | JN902307 | F:TGCTAACACGTCAGCTCGTAC  R:TTGACTTTTGGAATGCTGGAGGT | 57.5 |
| scaffold651_30754 | JN902308 | F:CCCTTGGCAAATACACTGCAA  R:AGGCCTGTGTCCATAGGATG | 57.5 |
| scaffold404_19771 | JN902309 | F:GCAGTCTGCTGCTGAGATCA  R:GTGAACACCACTGAGCCAAAAG | 57.5 |
| scaffold2430_67128 | JN902310 | F:GTCATCATCATCCACAGGTGCT  R:TCTGTGCTCTGAACTTCCCTG | 57.5 |
| scaffold2020_62950 | JN902311 | F:CGTCACATCTGGTTCTTTGAGGA  R:CATAACAACCAATGAGGCCTCTGA | 57.5 |
| scaffold351_17593 | JN902312 | F:GTCGTCCACTTGGTCAGCTTTA  R:CCTGGATGTCCGTTTGTGC | 57.5 |
| scaffold888_37653 | JN902313 | F:GACAGAGTCGTGCCTTTTAGCA  R:ACACAGTCGTTCGCTTATGTCAA | 57.5 |
| Scaffold080444 | JN902314 | F:TTGTCGACATGAAGGCAAACG  R:GAGCTGGGTTCAGGACTAATGA | 57.5 |
| Scaffold079345 | JN902315 | F:TGTCAGAGGCAACAGTCATTCA  R:AGACCAGGCTTAGTGGAGATG | 57.5 |
| scaffold2020_62952 | JN902316 | F:TGACTCACACTTTCCTGATGTGG  R:TCCTGCTGCTAAATCATCGTGTC | 57.5 |
| scaffold8099_71896 | JN902317 | F:CCGACGAGACAGACTGTTGAA  R:TCCGATATTGTCCAAACACTTGATTTCC | 57.5 |
| Scaffold000270 | JN902318 | F:ACCAGTGTGTACTCTACAGGTGA  R:CTGTACCCTGTGAGGCAGA | 57.5 |
| scaffold3676_70735 | JN902319 | F:AGGGAACACATGGAAGAATGGAG  R:ATTGCTGGTTCTCGCTGAGT | 57.5 |
| Scaffold076322 | JN902320 | F:CGTCAGTGGTTACAGGCAAC  R:CAATGACACCCTTGTCGTTCG | 57.5 |
| Scaffold078150 | JN902321 | F:CTGTAATCCACACAGACGGTGA  R:AGACAAAAGCCGGCAGAGTA | 57.5 |
| Scaffold000113 | JN902322 | F:GGCGATGTATAACGGGGTGA  R:GGTGCCTGGAAGAACATTTCC | 57.5 |
| scaffold470_22403 | JN902323 | F:CTGTAAATCACCTGCGTTGGAC  R:AAACCCACCTCTTCCACTCC | 57.5 |
| scaffold1349_51461 | JN902324 | F:AGCGAGGCCTTATATCACTGTCTG  R:CCTTCAGCTGTGTTGTCTCAGTGT | 57.5 |
| scaffold683_20653 | JN902325 | F:TCCCGTGAAGTAGCTGTGG  R:AGAGCGTCTCTCCCTCTTGA | 57.5 |
| scaffold6657_71709 | JN902326 | F:GTCATAAGAACGTGGAATTTAGCCAGA  R:CACCAGTACGTCACTCCAGT | 57.5 |
| Scaffold074485 | JN902327 | F:TGCGTGAGTTTCAGAACTGGT  R:ACTGGCACGTTCTCCATCA | 57.5 |
| scaffold1611_57313 | JN902328 | F:TCAGAGAGTCTCCACATCTGC  R:TGTTCACCAACACACAGGGA | 57.5 |
| scaffold235_10985 | JN902329 | F:CTGATGTTGGCTTCAAAGATGCTG  R:CTAGCGAAACCCTGACAAACTCA | 57.5 |
| Scaffold000102 | JN902330 | F:ACTTTCAGGACCCACATTCCA  R:ACATGTGGCTTTTGACTTCCAG | 57.5 |
| scaffold1611_57307 | JN902331 | F:TCATCGCCACGGTGATGTC  R:AGGTTCCACCCTGTCAACTC | 57.5 |
| scaffold3_539 | JN902332 | F:CTGGTTTACTGCCCACTGC  R:GACGTCAACAAGGACGAGGA | 57.5 |
| scaffold2104_64144 | JN902333 | F:CTGGGAGCTCCAGTGTCTT  R:CGTGAGGCAGCAGATGTTTAC | 57.5 |
| Scaffold078923 | JN902334 | F:TGCAACTGAGAGAACGGGT  R:AAACTTTGCCAGGGGTGG | 57.5 |
| scaffold1624_57230 | JN902335 | F:GACCGTAGCAGACAGGTCA  R:CAGAACCTGCCTCACACAAAG | 57.5 |
| Scaffold078819 | JN902336 | F:TGGTCAAGGGTCTTCATCCAC  R:CATCCCTACCAGTGCTGAGAA | 57.5 |
| scaffold409_20762 | JN902337 | F:GACACTGAATGGGCAGTGTATGT  R:TGGAGCAGATGAACCGTCTGTA | 57.5 |
| scaffold2998_69647 | JN902338 | F:CACAGGATTTCTCCAGTCGTTGA  R:TCCTTTTACTCCTCCCCTCCT | 57.5 |
| scaffold402_20228 | JN902339 | F:CACCAGTGCTCGTCTTCTGT  R:CACCAAAAACTGCTGGAATGTGAA | 57.5 |
| Scaffold000176 | JN902340 | F:GCCTGTATCTGACCCTTGTGTA  R:CACCGTTTGTCGCTATCTTCC | 57.5 |
| scaffold1719_58856 | JN902341 | F:AACCTCACCGTTTGTCGCTAT  R:TGCATGAAGCTCATTTGGTGAATG | 57.5 |
| scaffold1099_44501 | JN902342 | F:CCAGGGAGATGATATTGACAACAGG  R:TTTGAACCTCCCTCTCTCTTCGT | 57.5 |
| scaffold1298_51111 | JN902343 | F:TAGAAGGAGACAGACAGAGGCTTC  R:TCCCAACTGTGAAGTGGCATAC | 57.5 |
| scaffold1298_51107 | JN902344 | F:CAGCATTGGACTGGTAAACAGC  R:AGTCTCCTGTGGTACTGGTTGAT | 57.5 |
| scaffold375_18574 | JN902345 | F:CCATCTCCTTCTCCATTTCCACA  R:CGTTGCTGTGTTCATCTTACCC | 57.5 |
| Scaffold000163 | JN902346 | F:GGGTACCAATTGTGGGACATAC  R:TGGGACCCACAGGAATTATACAC | 57.5 |
| scaffold499_23574 | JN902347 | F:GTGAGCTGGTTTTGTGGTGAC  R:GAGAACCTGCTCTGCTCAGA | 57.5 |
| scaffold908_38918 | JN902348 | F:TGCCACTGCACTTTACATCCT  R:TGTGGCATCTGTTGTGTTTATGAGA | 57.5 |
| scaffold1544_57049 | JN902349 | F:GATGAGGGAGGTCATCTGAGG  R:CCAGATACAGTGGAGGTCGTC | 57.5 |
| scaffold215_7592 | JN902350 | F:GAGTCAGCAACGGTCAGC  R:TTGGGCTCTGGTGTTTGC | 57.5 |
| Scaffold079663 | JN902351 | F:ACACCGTCATAGTGTTTGGCA  R:ACAGCTTGGCAGTGTTCTCT | 57.5 |
| scaffold447_21249 | JN902352 | F:AGTTCCAGGACGTTTGCTTTG  R:GCTCTGGAAGGACAAGACAGG | 57.5 |
| scaffold1873_61125 | JN902353 | F:TAGAGCGTAGTTGGTGCCATG  R:TGGCACAAGGACAACGTAGAAA | 57.5 |
| scaffold569_26604 | JN902354 | F:TGTTTGTGTTCCCAGCATTCTCA  R:TTGGCGTTTGAATGTGGTCTA | 57.5 |
| scaffold5182_71517 | JN902355 | F:GAGTCGCACAAAGAACAGGGGA  R:TGGCTTATGAAGGCTGACCGTG | 57.5 |
| scaffold4620_71383 | JN902356 | F:GCTGGAGTTGGTGAAGGAGA  R:TGGACGCTAATCAGAGGAACAATG | 57.5 |
| scaffold687_31557 | JN902357 | F:GACAAATGTAGACTGCAGGGAG  R:ATAGGCGTCACACGTAAGGATG | 57.5 |
| Scaffold080390 | JN902358 | F:TGCATGAGAGAATTTACACCCGT  R:GCAACGAATGACACAGAGGAATG | 57.5 |
| scaffold340_17563 | JN902359 | F:ATTGGGAAAGCCCTTCTTTAGGT  R:ATGGAAGTTCTTTGTGTTAGCAGGAT | 57.5 |
| scaffold340_17565 | JN902360 | F:CGGCTAGCACAACAAACAAGAG  R:CTGTGACACAACAGACTGGATACA | 57.5 |
| scaffold1764_59160 | JN902361 | F:AGAGATGTGGCCGAGTAAATCAGA  R:ACACACAGTGTCATCGGCATAC | 57.5 |
| scaffold1994_62843 | JN902362 | F:GTCTGCGACAGAAAGAGG  R:TTCACACCAAAATAACACTGGAA | 57.5 |
| scaffold1764_59154 | JN902363 | F:GTTCAAGGTTGGAGTTCTTGGAGT  R:GAGGGCACAGTGAAGATTGGA | 57.5 |
| scaffold1197_47382 | JN902364 | FlTGGTGAGTGAAGAACATTGGC  R:ACTGCCATCCTACAGTAGAGTC | 57.5 |
| scaffold2188_64837 | JN902365 | F:GCGTAAACTTCCAGCGTTCAG  R:GAAACAAAACGTCAACCGAGACG | 57.5 |
| scaffold2368_66664 | JN902366 | F:GTAACAGGGCGTAGCTGTGT  R:GAAATCAGTAACCAGGCGGC | 57.5 |
| scaffold99_5221 | JN902367 | F:TGCTCCAACGGGATTTGTCTTTT  R:TCAGGCAAACTCAGTGGAAGAG | 57.5 |
| scaffold2320_65962 | JN902368 | F:ATCGCTGGTGTAAATGTTGCG  R:TGCATTTGCTTTTGTGTGAGTGAAA | 57.5 |
| Scaffold076662 | JN902369 | F:ACCCTGGTACTCACTTCGG  R:ACCCAGGTACAGTATGTGAACAG | 57.5 |
| scaffold000377 | JN902370 | F:AGCGTGAGAGAGACCTTAACC  R:GTTCTCGCGGATGCATGT | 57.5 |
| scaffold1442_54298 | JN902371 | F:GTCACGTACCCTCCATACACC  R:GAGTCGTACGTGGATAAGAGTCGA | 57.5 |
| scaffold365_18002 | JN902372 | F:CAGCTTTAACCCTGGCAACAG  R:TCATCCAGGACTCAGCCTTG | 57.5 |
| scaffold2251_65540 | JN902373 | F:GTACATCCTCACACGGACGTT  R:GTGGCTGCTCCAATCAAAGG | 57.5 |
| scaffold2251_65541 | JN902374 | F:CATCAGAAACTGATGTCGGCTCAT  R:CCAGGTTTCAAAACCACTTCACG | 57.5 |
| scaffold1906_61429 | JN902375 | F:TTGGAGCCACATTTCTGG  R:ACCAATGGGTCATTTGAGG | 57.5 |
| scaffold486_23323 | JN902376 | F:CACAATTCCGAGCTCCCAGATA  R:TGCATGAACCTGTAGCACACA | 57.5 |
| scaffold076206 | JN902377 | F:CTGGAGGTGGAGTGTGACT  R:TCTGTGAGCAGATTGTAGGTCC | 57.5 |
| scaffold365_18009 | JN902378 | F:ATATGCCTGTGTGTGGAATAAAAAT  R:TTTGACACGCGTCGGACAT | 57.5 |
| scaffold3045_69830 | JN902379 | F:GTCAGAAACAGCTGAGGAAGC  R:GACCTTGATGACCCCTCCTTTAA | 57.5 |
| scaffold897_38337 | JN902380 | F:ACGACGGTCAAGCACTCATAAATC  R:GATGGCATGAGGAAAAAGCAACAG | 57.5 |
| scaffold1906_61423 | JN902381 | F:CTCCTCATCACAACCCAATC  R:TCCGTACAGTTCATCCTCTC | 57.5 |
| Scaffold079499 | JN902382 | F:CTTCTCCTGATGGAAGGTTCGA  R:ACCTGCAGCCTCCTATCATC | 57.5 |
| scaffold1060_43080 | JN902383 | F:TGTGGGGCAAATAGGACTGAAAT  R:GGGAGGAATAATGTTGGGATTGC | 57.5 |
| Scaffold000325 | JN902384 | F:TCAAGGACTGTGTTACGAAGGAG  R:GAACCCAGATGAGCTGGATGA | 57.5 |
| scaffold1740_58904 | JN902385 | F:GGTCGCACAAATGTGAGATACA  R:GCCATTTCATCCAAACCTCTG | 57.5 |
| scaffold1934_62242 | JN902386 | F:TCCAACAGGACGTTGACATTAGG  R:ACTTCAAACTCTGTGCGATGGTT | 57.5 |
| scaffold1354_51497 | JN902387 | F:GCTAACAGTTTTCCCCAGCAG  R:AGCTGTCCGTCAAATTCACCT | 57.5 |
| scaffold3153_70072 | JN902388 | F:ATTTGTCCAGATACAGGTCAT  R:CCTTGGGCAAAGAATAGTAC | 57.5 |
| scaffold3153_70071 | JN902389 | F:CAGGTGTCCTTTCACAACTGTG  R:TGTTACCAACAGGGAGTCGTT | 57.5 |
| scaffold83_3406 | JN902390 | F:GACTCAGGTGCTCGAGTTAGAC  R:GAAAGGGAGTGTGGACTTCCA | 57.5 |
| scaffold1823_60337 | JN902391 | F:TCACTCTTGCTTCTGCACATGA  R:AAGGCCTTCATGTCCTCTGAAT | 57.5 |
| scaffold15_344 | JN902392 | F:GTGAAAGCAGCACACTGCA  R:GCAGAAATGTACCAAGGTGTCAG | 57.5 |
| scaffold662_30602 | JN902393 | F:TAGTTTATTATCCCACTCCCTGCG  R:GCAGGAGCCTTTTTACAACTCAAA | 57.5 |
| scaffold3741_70921 | JN902394 | F:TTCTCTGCTTCCGACTTTCACC  R:CATCTGACATTTCAGCCTGCG | 57.5 |
| scaffold372_12391 | JN902395 | F:GAGGCACAGAGACATGGTCA  R:TGGGAAGAGTGGGAAGTCAC | 57.5 |
| scaffold893_37729 | JN902396 | F:GGGATGTTATTCATCTGTGCAGC  R:GCTGGTGGATAAATGCACTCC | 57.5 |
| scaffold1681_58257 | JN902397 | F:ACGATGACTCATACGACGAGGA  R:AAAAGTAGGGCACTGGGACAAA | 57.5 |
| scaffold1681_58260 | JN902398 | F:CAGTGTTACGCAGCCTTTTGTC  R:CTTCGTGAAAACAGTCGTGACC | 57.5 |
| scaffold608_29071 | JN902399 | F:CACACAAACCCACTGATCCATTC  R:GGAGACAGCTGAAGTCCACT | 57.5 |
| scaffold508_23760 | JN902400 | F:GCGAGTGGGGTCAACTCTAATAA  R:ACAGCGAGGGAGAGAACTG | 57.5 |
| scaffold103_5051 | JN902401 | F:CCTGTGGGAACCTGAATTCTT  R:GCAACTTCCTCCCACTGTCT | 57.5 |
| scaffold628_29487 | JN902402 | F:GACCCACAGAATGTCTGCTGAA  R:GATCAGTACCAGGACATTGCACA | 57.5 |
| scaffold58_3304 | JN902403 | F:ACGCCCCTCATTCTGAAAGTC  R:CCAGTCTCAGGTGGCTGATT | 57.5 |
| scaffold1228_48640 | JN902404 | F:ACTGTACAGTAGGCTGCCTTC  R:AGGTGCAGGGAGGGTATG | 57.5 |
| scaffold531_25352 | JN902405 | F:GTTTCCATTGCAGAAGAGCCG  R:CGCTCTTTGAAGCAGCACAT | 57.5 |
| scaffold808_35270 | JN902406 | F:TCACTGCAGGAAGTGAAAAGGA  R:GCTCTCCAACAAACAGGTGATG | 57.5 |
| Scaffold080489 | JN902407 | F:AGATGGAAAATGCAAAACTCATCC  R:GCTGAAAACTCTCAGACCGTA | 57.5 |
| scaffold884_37426 | JN902408 | F:GGTTTCTTTGGTTCCCCATCTTC  R:TCAAACCGGACAAAGACAGACAA | 57.5 |
| Scaffold074306 | JN902409 | F:CTGCTGTGACTCATCTGTGC  R:CCCATTCAGTGGTGACAGTG | 57.5 |
| scaffold1420_53607 | JN902410 | F:GAAAACGCTCCAAACACAGACAC  R:TGTTTCCAACAACACTGTCCAGA | 57.5 |
| scaffold1114_44924 | JN902411 | F:CTGGCGAGTCCTGAAACATCT  R:CACGTACACAGCTGCATGTC | 57.5 |
| Scaffold075862 | JN902412 | F:TCTGACTGGAGGAATACACGC  R:CCTCACATCCCTGGAACACA | 57.5 |
| scaffold3321_70434 | JN902413 | F:TTGCAGTGGTGAAGATCAGC  R:GAACCACAGACGAGGCCTAA | 57.5 |
| scaffold1258_49011 | JN902414 | F:CAGCGGCCAGATAAGACAATC  R:TTGTGCTCACACTCTACTCAGC | 57.5 |
| scaffold1141_45311 | JN902415 | F:CCCCTCCTCCTCTCTCTCT  R:ACAATCAGGCTGCATGCAATATG | 57.5 |
| Scaffold078830 | JN902416 | F:TCACAGCAACGGTAGTTTTCCA  R:CAGATGATCCCAGGGTGGAT | 57.5 |
| scaffold660_30731 | JN902417 | F:CAACTGCCTGTGTCTCAGTG  R:AACTCAGTGCAGGTGGAGAG | 57.5 |
| scaffold660_30734 | JN902418 | F:CTGGTCTCATCTGAAGTGTATACGG  R:GCTGCACCAACGTCTTAACTG | 57.5 |
| Scaffold000391 | JN902419 | F:CACCGGTGTCAGTGGATGTA  R:GATTCGTATTCCTTCCGCGTTTC | 57.5 |
| scaffold2270_65667 | JN902420 | F:TCATTCCTGCAGCACCTGAA  R:GGCCAAAGCACCACAGTTAT | 57.5 |
| scaffold2005_62887 | JN902421 | F:GACTCCAGTCCAGACAATGCA  R:GGAGGCTGAGAGTGAGAAAAGG | 57.5 |
| Scaffold000356 | JN902422 | F:GCCATGTCTTCCTTATGCAGTG  R:CAAACCACTGTTGCTCTCGAC | 57.5 |
| scaffold498_23471 | JN902423 | F:AGTCGAGTTGAGATGAGGTGAGAA  R:CTCTGAGGCTGGACTGATATGTG | 57.5 |
| scaffold27_281 | JN902424 | F:GCTGGCCCTAATGAAGTGTCT  R:GACCCGGCGTAGTAGATGAAC | 57.5 |
| scaffold627_29518 | JN902425 | F:TTTCTGCTCCTGCTGTTTGC  R:AGCACCATGTGAGGACTGAC | 57.5 |
| Scaffold080375 | JN902426 | F:TGCCTCATCTTCTCCGTCC  R:TCAGTGCCAACTCCACTCTC | 57.5 |
| scaffold954_39899 | JN902427 | F:AGCAGCAGCAGCAGAAGTAA  R:ACACCTTGATCCTGAGTGAGATG | 57.5 |
| Scaffold000306 | JN902428 | F:TTAGCTCCAGACCCCAGAAC  R:CCAGAGCTCCAACATCAGC | 57.5 |
| scaffold352_17499 | JN902429 | F:AAACCCACTCCTCTGCAAATCA  R:GATGCTCTCGTGCACAAAATCTG | 57.5 |
| scaffold1181_46598 | JN902430 | F:AGGTTAACCAATCAGATGAGCCG  R:TGTGTTTCAGCTGTCAGATCCAC | 57.5 |
| scaffold533_25540 | JN902431 | F:GAGAAGAGGCTCTAACCACCAG  R:AGTCTGAACCAATGCAGCCATA | 57.5 |
| scaffold533_25474 | JN902432 | F;TGCAGTCTACACCTTAGCCTTAAC  R:GTGTTAGACAGATGCCCTTTTGC | 57.5 |
| scaffold3122_69947 | JN902433 | F:CCATCATCTAGACCACATCACAG  R:TTGATGGAAAACCTGATCAACTGAA | 57.5 |
| scaffold931_40176 | JN902434 | F:GTCCATGTCCTGAACGAGAGT  R:GACACACTGATAATGACCTGCCA | 57.5 |
| scaffold686_31835 | JN902435 | F:GTGGTCACTCTTTCAGGAAGCA  R:CATAGGTTGACGGAAGGAAAGTCA | 57.5 |
| scaffold2143_64422 | JN902436 | F:ACATTTCCCAGAACAGTTATTGCAATTCA  R:CCTTACCCCATGGTTAAGAATGTTATGT | 57.5 |
| scaffold225_10803 | JN902437 | F:TCAGAGAGACGTTGAATCCG  R:GAGTCTGAGGGAAGTGCTAG | 57.5 |
| scaffold2223_65565 | JN902438 | F:GAATTTTTGATTCGGGTGTTTCAG  R:GAAAAGTATGCCACACCTAATGGC | 57.5 |
| scaffold582_27999 | JN902439 | F:CACTAGGGGCTTCTTCGAGT  R:GCGTGATTTAGGTCACGTCTGA | 57.5 |
| Scaffold000422 | JN902440 | F:CTGCACTCAGTGTCCTGTTG  R:CAGGGACCCGTCTCTTCTT | 57.5 |
| scaffold1085_44234 | JN902441 | F:AGATCTGAGGCCATTCACAGTTG  R:TGTCCACACACTCGTTGACTTC | 57.5 |
| scaffold1525_55172 | JN902442 | F:CGAAAAACTGATAACGATCCCAAG  R:GGGTTTGGTGAAGACTGATGCTAA | 57.5 |
| scaffold2780_69312 | JN902443 | F:CGTCTTGGGTACAAGCTGTGA  R:TGGTGACCCCTGCTGTAAAG | 57.5 |
| Scaffold076797 | JN902444 | F:CTCAGTACACACTGACTGCCTT  R:GGTCCTTATGCAACCTGCTG | 57.5 |
| Scaffold077589 | JN902445 | F:TGATGGGTTCCAGGGTGTATC  R:GAACCGCCTCTTAGCCTCT | 57.5 |
| scaffold1953_62372 | JN902446 | F:TCCGTCTGCGTCTTGTGTAG  R:TGTTGTTTTGGGTGATCTGCAAC | 57.5 |
| scaffold379_19493 | JN902447 | F:CTGTGGCTCTTACCACTCGTT  R:AACCCTGAACCTAAACCTACCC | 57.5 |
| scaffold586_28587 | JN902448 | F:GAGAGGAAAGCCAGTACAAGGAG  R:CTCTTCTCCACCCACCGTTATTT | 57.5 |
| Scaffold000089 | JN902449 | F:GTGGCACAGACTTTTGCCTAG  R:CTTGTCTCCTGTGAAAAGCCATC | 57.5 |
| Scaffold079788 | JN902450 | F:CAGGCAAGCGTGAGAGTAGTA  R:GATCCTCCCAGGCACTGAT | 57.5 |
| scaffold1263_49312 | JN902451 | F:TGAATATCTGCTCTTTGTGGCAACT  R;GCCAGTGCATGACGAAGTTTAG | 57.5 |
| scaffold4670_71357 | JN902452 | F:CATGGCTGGAGCTCTGATGTTA  R:TGTACAGGAACCCGATTGTGAC | 57.5 |
| scaffold672_31081 | JN902453 | F:GTCATCGCATTGTGGAGCTAGA  R:TGCAGACGTGGAGGAAAGAG | 57.5 |
| Scaffold078212 | JN902454 | F:CACCCACTCACTCACTCGAA  R:CGCCTCTGATGAATAGCAGGA | 57.5 |
| scaffold1531_55245 | JN902455 | F:CCACAGAAGGTTCTGGCAATG  R:CCCCTCTTGTCTGAGGTTCT | 57.5 |
| scaffold855_36360 | JN902456 | F:GTTTAGGACTTGTTTCCCAATTAAAAGCAA  R:CTCAACATGTCAGGGCTGTAATGA | 57.5 |
| scaffold3370_1008 | JN902457 | F:GAAGAAGTGTGTGTGCGCTTG  R:GGGTTGGGAGCAATAAAGGAGT | 57.5 |
| scaffold1639_57815 | JN902458 | F:GCTTAAAAGTAACCCCTGCCAAG  R:GGTCTCTGCGTCCTCATTCAA | 57.5 |
| scaffold2585_68068 | JN902459 | F:AACGTGTGCATATATACGTTTGAGACA  R:GCTTATGATTGAGAAGTCTTGCCAGA | 57.5 |
| scaffold595_28658 | JN902460 | F:CACAGGAAATGAAATCCCTCTGGT  R:TTTGGACAGGGGAAATTCTGCA | 57.5 |
| scaffold2996_69760 | JN902461 | F:CTGTCTGGCCTCTTTGTCTGT  R:GCATCAGCAGGAGAAATTCAGGA | 57.5 |
| scaffold545_26174 | JN902462 | F:AGAAGGTGTCAGTCAAGCTGTC  R:GCAACCTTTGACTCCACTGC | 57.5 |
| scaffold1385_53192 | JN902463 | F:TCAGCACTGCAGCTTGGTTAT  R:TGAGTCACCATTGTGCCAAATCA | 57.5 |
| scaffold1067_43468 | JN902464 | F:CCCAGAAGGGGACTGAAGTTT  R:TGCTCTGTTCTCACACTTGCT | 57.5 |
| scaffold2138_64143 | JN902465 | F:GAACACATGCTCCATGAATGC  R:ACGAGTTCAAACCACGCTTC | 57.5 |
| Scaffold080118 | JN902466 | F:AGGCTGGAGCCTACTGAAG  R:TTTGTCAGATTCTCCCCTGCA | 57.5 |
| scaffold961_40009 | JN902467 | F:CAGGGTGGGGCTACAATGATA  R:ACCAACGCACCGTAGTTAAATCA | 57.5 |
| Scaffold076461 | JN902468 | F:AGTTCCAACTCGGCAAATAACCA  R:GAAAGGCCAAGAGCATGTGATG | 57.5 |
| Scaffold079519 | JN902469 | F:AGTCTGGCCTGGAGTTTGT  R:AGCTACCAGGTGAGGAGC | 57.5 |
| scaffold2161_64371 | JN902470 | F:GGACTACAGTGATGTGGCCTAC  R:GCCAGACTCAGGTCTGTGAA | 57.5 |
| scaffold632_29617 | JN902471 | F:GGTGATGAGAGCCACTCTGT  R:GACACACACATTAACTGTAACCTCCA | 57.5 |
| scaffold2161_64369 | JN902472 | F:CTCAATTCACGTGGCACCTACA  R:CAGCTGGATGCCTTCTTTGATC | 57.5 |
| scaffold1452_53955 | JN902473 | F:TTCTGTCGGGCTTGTGTTCTT  R:CTTTACATCATCGTCGCACCAC | 57.5 |
| scaffold626_29427 | JN902474 | F:TGGTGTGTAGTTGTGCAGTGT  R:TGAGACCCCAACCACTCATTC | 57.5 |
| scaffold647_30624 | JN902475 | F:CGTCTGGTTAGTGCTGACAAACTG  R:GATTAGTAACAACCTGGGGAGGCT | 57.5 |
| scaffold5533_71563 | JN902476 | F:CACGTCCATTCCTCATCCAGTA  R:TCTGGATCTGTCTGTGGGAGA | 57.5 |
| scaffold1921_62599 | JN902477 | F:CTGATGTGTCTGCATCTGGAGT  R:TCTGTGACGTGAAGGAAGAGAGA | 57.5 |
| scaffold301_14617 | JN902478 | F:GCTTTAGACCAGGACTGCTTCT  R:TCACACCTGAGCAGACAAACAA | 57.5 |
| scaffold604_29006 | JN902479 | F:CAGCCTGGCTTCGAGATAG  R:CCTCTGACGCTTTATCAGCTC | 57.5 |
| scaffold5533_71564 | JN902480 | F:CACGTCTCTGAACGCGTAATGA  R:TTTCCGTTGACCCTCTGCA | 57.5 |
| scaffold1890_61225 | JN902481 | F:GAGTCGTAGGCGATGTGTCA  R:TGTCCATCTGTCTGTCGCAC | 57.5 |
| scaffold332_17756 | JN902482 | F:GGGTTTTAATGGCTTCTCTGCTG  R:TCACTGTGGATTCTTTCCCTCATG | 57.5 |
| scaffold308_15469 | JN902483 | F:GGAGGAGTGGCTGTGCTTTAAT  R:TAATGCTCTCTGTCCTCCCTCT | 57.5 |
| scaffold767_34404 | JN902484 | F:GTGCAGGTGTACTGTGGGTA  R:CAGAGGACGGAGATGACGAAC | 57.5 |
| scaffold5997_71637 | JN902485 | F:CTGAACACCAACCAACGACAA  R:TGTGCAGCAGGCGTTTAAG | 57.5 |
| scaffold767_34395 | JN902486 | F:GCGCCTTCTACCTCTTCAGT  R:CCTCTCTGATCCACCAGTACG | 57.5 |
| scaffold71_7577 | JN902487 | F:TTGTGCATCCCTGAGACAG  R:GGCACAGTGGGAGTGATT | 57.5 |
| Scaffold074816 | JN902488 | F:CACTCCTCACTACAGACATAACCC  R:GGAGACTGGAATGTTTGGTCC | 57.5 |
| scaffold1447_53897 | JN902489 | F:TCCACCATACAGGAGCCC  R:AAAAGCCTTCCAGACCAGACA | 57.5 |
| scaffold513_24073 | JN902490 | F:CAGACAGCGATCAGACGACA  R:GGAGGAAGCCCAGAAAGCTA | 57.5 |
| scaffold809_35286 | JN902491 | F:TTTGTCTCCTGGGAAACTCCTTTAC  R:CCCACTGCTGTCCATACTTCA | 57.5 |
| Scaffold077280 | JN902492 | F:AAGGCTCACGTGACATCATCA  R:CTTCACCAGGCTCCATCCTA | 57.5 |
| scaffold2254_66591 | JN902493 | F:TGCAGGGGTTTTGTTGCTCTAT  R:CTCCAGCACAGACGAACTTTCA | 57.5 |
| scaffold1242_48809 | JN902494 | F:AGTGTGATGGTGTTGGTGCA  R:CTCTGGCAACTGCCAAGAAAAG | 57.5 |
| Scaffold000195 | JN902495 | F:GTTACAGTGACGGTTGAGGACA  R:ACCTGAGCCACTTCAGCAT | 57.5 |
| scaffold1109_44563 | JN902496 | F:CCCTGAGTTGCACAAGGAATCT  R:TCTCACCGTTCTCTCTCTCCAG | 57.5 |
| scaffold1635_57449 | JN902497 | F:CAGACCTGGACCAGTGGAAA  R:TGAATCCTCTGTGTGGGAGTG | 57.5 |
| scaffold347_16763 | JN902498 | F:TTTCGAGAAGCTCGTAGCTCG  R:CAATGGCAGCAAATCAATAAATATCTGAAC | 57.5 |
| scaffold2010_62849 | JN902499 | F:CGTCCAATCATCAAGCAGAAGTC  R:CTTTGTGTAATTTAGCCCAGGCC | 57.5 |
| scaffold600_29188 | JN902500 | F:GCGTGTACACAGGGTGGTAA  R:GAGGACACTGAGGACAGAGAAAC | 57.5 |
| Scaffold075277 | JN902501 | F:GTGTTTCCTCGTTGTCAGCATC  R:GGAGGTCAGGGATGGGATTAG | 57.5 |
| Scaffold000078 | JN902502 | F:AGGCTCACCACGTGACTAC  R:TGACATCACAGGCACGAATGA | 57.5 |
| scaffold2773_69031 | JN902503 | F:GACGGAGCCAATGTTGGTTTATTA  R:CCAAGCAAGGATGAAGAAGAAGAG | 57.5 |
| scaffold478_23068 | JN902504 | F:AGCCTGTGTTGGACTGATGTT  R:ACCGTTTCCCTTCGTTGTATTCTC | 57.5 |
| scaffold2305_65829 | JN902505 | F:GTCTCCAGCCTCCAAACAGA  R:AGCATGGACTTCCAGTTGGATT | 57.5 |
| scaffold147_7102 | JN902506 | F:TGTTGCCCAGCAGTCTCTTT  R:GTGGGTTGATGAGCAGTGAAG | 57.5 |
| scaffold147_7111 | JN902507 | F:GGTTGAGGGCCAAATAATGGG  R:CGCACTGTGCCGTACATAAG | 57.5 |
| scaffold2527_67825 | JN902508 | F:CGGTGCATTAGCACTCTTTAGGA  R:CGACGCAAACGTGTCATTATCTTTG | 57.5 |
| scaffold7590_71810 | JN902509 | F:GTGACACATTTGAGGCGTTGAC  R:TATATGTGGGCTGCGAATATGGG | 57.5 |
| scaffold535_25182 | JN902510 | F:CGACTACAGTTTCCTCACACAGG  R:TGGGATTAGGGATCTGAAGTGC | 57.5 |
| scaffold249_12377 | JN902511 | F:GTATAAGGGAAAGTGCCCAGGA  R:GAATCTGATTGCGTTCAGCAGC | 57.5 |
| scaffold5063_1113 | JN902512 | F:ATCCACTGTTGGCGTAATCTGAGT  R:TGTTTTGAGCTCCACTCATGACTTTG | 57.5 |
| scaffold1790_59368 | JN902513 | F:TCTGGGTTGAATGTGTTCAGAGAC  R:ACAGGAACCACGACTGACATC | 57.5 |
| scaffold543_25681 | JN902514 | F:AAGTTCAAAAAGTCGCCAGCAC  R:CACGGCGTTGAAGATGTCC | 57.5 |
| scaffold3018_70062 | JN902515 | F:ATCCATCATTCTCCCAGCGC  R:CTCAGCAGGACGTCAACACTC | 57.5 |
| scaffold108_5289 | JN902516 | F:GATCCCAGTACGGTCCAATCAAT  R:TCCTGACCCAATCTCAGATCCT | 57.5 |
| scaffold1565_56149 | JN902517 | F:AGACGTGCTGCCAGTGTTTA  R:CTCCACGTGTTTTCACCATCA | 57.5 |
| scaffold1986_62579 | JN902518 | F:TGCAGAGAAGTCTCTTCCAAGG  R:CATGCGTGATCTCTGACAGGT | 57.5 |
| scaffold325_15675 | JN902519 | F:TGCAGGATAAGCAGACAAACTCAAA  R:CACTGCTACAGGCGTTGAC | 57.5 |
| scaffold1940_62137 | JN902520 | F:ACAGAGGAGCAGAAGGAGGT  R:TCACCCACCAATACCATCAGTC | 57.5 |
| scaffold1422_53462 | JN902521 | F:AGTTTCACCAGCAACCTCCTT  R:ACGCAGGTCTGAACAGGTTTT | 57.5 |
| scaffold950_39778 | JN902522 | F:GGACACTAGAGGCAGCGT  R:CGGCAAATACTTTACAACAAAGGCT | 57.5 |
| scaffold4962_71431 | JN902523 | F:CAACGGCATGAAGAAGGACTG  R:CACTTCCTGTTCTGGGACCT | 57.5 |
| scaffold2230_65448 | JN902524 | F:GGGCACAAGAAAAGCACTCAC  R:TGGAGGATGGTGTTTGGAAAGG | 57.5 |
| scaffold149_7459 | JN902525 | F:AACAACAGGCCCTCAAAGACA  R:CTGGGTTTGGCAGAGTTTGAC | 57.5 |
| scaffold2501_68002 | JN902526 | F:AGCCGAAGCAGAAAAAGTCTGT  R:TGATGTTCCTGGATGCTGCTTT | 57.5 |
| scaffold2163_64406 | JN902527 | F:CTAAGTTCTGGGGCACGAGTT  R:AGGGCCACATTTTCACAGC | 57.5 |
| scaffold1497_55115 | JN902528 | F:TGTGGAAGGTGGACAGGTATTG  R:CAGGCCTGAGATCAGTGTTCA | 57.5 |
| scaffold2158_64365 | JN902529 | F:AGGTCGTTATTGAAATGCATGGTGT  R:GCTCTGGCATGAACCTGAAAAAG | 57.5 |
| scaffold2445_67357 | JN902530 | F:GGCTGTACACGCTTCAGTCA  R:AGTGATCCCTGGCCACTACA | 57.5 |
| scaffold63_3117 | JN902531 | F:TGGCCAATTATCACCTCCAGTAAAAG  R:ATAACAGGCTGGAGACACTCAC | 57.5 |
| scaffold3905_70961 | JN902532 | F:GCACATTGGTCAATTGTTCCTT  R:CACCAATGGTCACATCAGC | 57.5 |
| scaffold1921_62614 | JN902533 | F:CGATGGACATCCGTCCATTATCT  R:GGTCAGCTGGTGTTAAGTGC | 57.5 |
| scaffold1492_54602 | JN902534 | F:ACATGGGGGTCTCAATCTACCC  R:CCTGTCCTGACCCTGTGTCT | 57.5 |
| scaffold63_3139 | JN902535 | F:CATTCTTAAAGCGACCACATGCTG  R:TTATGACTCCGCCAGCTGATG | 57.5 |
| scaffold1580_56114 | JN902536 | F:GAACACAGGGGGTGAGTGT  R:GGACTCACGGCCATCACATTA | 57.5 |
| scaffold349_18869 | JN902537 | F:CACCACTGTTTTCTGGGTGAAG  R:CCTCTGTGATTCCATCCTCATCG | 57.5 |
| scaffold978_40424 | JN902538 | F:ACTCAACAACAACGCTAAGGATATAAACA  R:AGAAGATGGCAGCAAAGCAAAC | 57.5 |
| scaffold1217_48164 | JN902539 | F:AGGCTTTGAAATGTGTCCTGCT  R:GCGAGTTTCTGCATGTGAGTG | 57.5 |
| scaffold1694_58369 | JN902540 | F:CATGCCAAGATCCACATTTCCC  R:TGTTGAGGGGAGTAGGAGAGT | 57.5 |
| scaffold126_6942 | JN902541 | F:TGTTGTTTCCTGTCTCCGTCTG  R:GTCACAGCTGCAGTCACAC | 57.5 |
| scaffold70_2626 | JN902542 | F:TGGATCGCCTGCTTCAGTAAC  R:CAGTGGGGTTGTACCTACCTTC | 57.5 |
| scaffold997_41297 | JN902543 | F:CACAGAGACCAAGTTCTCTAACGTC  R:TTGCACTGACCATCTGGATTCTC | 57.5 |
| scaffold1694_58358 | JN902544 | F:ATGAAGACTCGCTCAAACAGAAGC  R:ACATCTGTAGGAACAGAATCCCATCA | 57.5 |
| Scaffold075622 | JN902545 | F:GCAACAGCCACCACTGAAT  R:TTGGTCCTTCAGTTGACCACA | 57.5 |
| scaffold1272_49453 | JN902546 | F:GCAGTCAACGAATGCAAGTTCAA  R:TCCTACTGATTTCCAACTGTGGTC | 57.5 |
| scaffold663_33093 | JN902547 | F:CATTTACAGCCCATCAGTCCCA  R:AGCGTGTGCGTGTCTGTAAG | 57.5 |
| scaffold1602_56906 | JN902548 | F:TCAGATTGATGTTCCAGCCCTAATG  R:CGTTTGTGGGTGTCCATTTTTTAAG | 57.5 |
| scaffold1602_56905 | JN902549 | F:GGCTGTTGTTGAGAGTAAGAGGGA  R:GCGCCACTGTGTACTTAGTGTTTG | 57.5 |
| scaffold165_8420 | JN902550 | F:GGGGGACGAAATGAGAGAGAAAA  R:CACCGTCTTCACGTCCTGTA | 57.5 |
| scaffold1055_42698 | JN902551 | F:GGAGTCACATCCTGATCCAGTG  R:TGTTGGTGGTCTTCATGTTGGAA | 57.5 |
| Scaffold000051 | JN902552 | F:TGTGGGCAACTGTGAGAAAGA  R:CGGGTTGGTGTATTGGCTTC | 57.5 |
| scaffold349_18845 | JN902553 | F:AGCTGGAGTAACTGGAGACAAC  R:GTTCTCATGGTTTTCCCTCATCG | 57.5 |
| Scaffold000087_922 | JN902554 | F:TCACACATGGTGAGAGCACAA  R:ACCCAGAAATGGACAGAGTTTGT | 57.5 |
| Scaffold076395 | JN902555 | F:CCACTATAAGGCAGCTGTGC  R:GCCTAGCAACAGGGCAGTA | 57.5 |
| scaffold216_10757 | JN902556 | F:TGGACACTCCTTGAGAAAGCATG  R:ATGACACTCCGACAGTTTCTGG | 57.5 |
| Scaffold076829 | JN902557 | F:TGTTTGCCTGTCACTGAGGA  R:TATCCCATCTTGTCAGGGTGTG | 57.5 |
| scaffold628_19400 | JN902558 | F:CAGGTCACTCGAGGACAAGAA  R:TGGGAGGATTGGGCTTGTTAT | 57.5 |
| scaffold426_14789 | JN902559 | F:GCTTGACTTTAGGGCGAGG  R:AAGATGACGGATGGATGGTTCA | 57.5 |
| Scaffold080451 | JN902560 | R:TGAGTCTCTCCTGCACCAATG  R:TCTGCACAACGTCCTTACACTAC | 57.5 |
| scaffold363_18220 | JN902561 | F:ACTGCGCTTCAAAGCAAAGAC  R:TGCAGTTTTCCCTCGCAGTA | 57.5 |
| scaffold1628_57677 | JN902562 | F:GTCCTTAGATCACAGCACCTGT  R:CTGCAGCTCACCTTCAGTG | 57.5 |
| scaffold347_11967 | JN902563 | F:ACCATTACCGAGAGAGGTTGTC  R:TTCATGCAGCAGTGCATCC | 57.5 |
| scaffold2329_66082 | JN902564 | F:TGCAGTGGAAGCACAACAAGTA  R:GCTAGCTGAGAAACATGCACAG | 57.5 |
| scaffold397_13245 | JN902565 | F:CTGAACGGACTCACTGAAGGT  R:GAGCTGGAGTCCATGTGC | 57.5 |
| scaffold1488_55203 | JN902566 | F:GTTTGAGGAGGAAGAGGAAGACG  R:GTGGGTGTGTCCACTTGAAAAG | 57.5 |
| scaffold226_10829 | JN902567 | F:ATGACAATCTGACACCGAGTTATCT  R:TTTCCATCGGTCTGGAATAAGACA | 57.5 |
| Scaffold080355 | JN902568 | F:GGCCGTGAACCAACACTTT  R:TCAGGTCCATCGCATTTAGTCC | 57.5 |
| scaffold1317_51166 | JN902569 | F:ATCACCAACTTTGCCTCTGG  R:TTCACTCTCCTTCCTGGATGA | 57.5 |
| Scaffold080359 | JN902570 | F:GCAGCGGATAAGAGGGAAGA  R:GGTTGTAGTAACCCCCACCT | 57.5 |
| scaffold552_17719 | JN902571 | F:CCGAGAGCTGAGGCTGAAATA  R:CAGGATGCAGCAGAGAACAG | 57.5 |
| scaffold1150_45464 | JN902572 | F:CCTGAACACTCACGGAGAACA  R:TGATGCTCACACTCCTGTCC | 57.5 |
| scaffold1143_45424 | JN902573 | F:TGTCCTTAGACTCCAGGGC  R:GAACTTCGAGAATCACTGATCAACAATG | 57.5 |
| scaffold3686_70732 | JN902574 | F:ATGCCCTTGGCTTTACACTAGG  R:TTAGCTGCGAGAAGGTGAGAGA | 57.5 |
| scaffold826_35961 | JN902575 | F:TTGTGTGTGTTCCCACTGATGT  R:CCGTACCAGAGGACACGTT | 57.5 |
| scaffold4219_71183 | JN902576 | F:CCCACTCCTCATTTGCTACCT  R:TGATTTTGCCACAACTCTGACGT | 57.5 |
| scaffold1333_51187 | JN902577 | F:TGAAGATCTACTGGAGACACAGCC  R:CCCTGAGACAGAACTAAAATGACGA | 57.5 |
| scaffold1844_60005 | JN902578 | F:TACAACCCTCCACTACGATCATGA  R:ATCTACGGTCTGTGCTCCTGA | 57.5 |
| scaffold2024_63217 | JN902579 | F:CCTCGTTTTTGGGAAACCATGTG  R:GCTAATGACATTTTTGTGGCGTGA | 57.5 |
| scaffold1511_55644 | JN902580 | F:GGTTCCTCGATGGTGTCTGT  R:TCCAGGCTCAACTCCACTCT | 57.5 |
| scaffold4089_71030 | JN902581 | F:AATTCAACAGTTCTCCACTCAGCC  R:GTTTTCATTATGCTGCCCTCAGTC | 57.5 |
| scaffold596_29029 | JN902582 | F:ACGCTGCTGCTTTTAGACAACTA  R:AAACATTGAACTTTGCTGTATTCA | 57.5 |
| scaffold1415_53179 | JN902583 | F:GATTAGCCACGTATTTGTGATGGATCA  R:AGCACTGATCATGTGTTGAAGCA | 57.5 |
| scaffold376_18254 | JN902584 | F:AAAAGTAGTGTTGTGCAAGTACTGTGTT  R:GCAGAAATCCAACTGTCCAGAAT | 57.5 |
| scaffold4124_71074 | JN902585 | F:CAGCTACACTGGGGGAGAAA  R:GTGTGGATGTCGTCACGTTCT | 57.5 |
| scaffold74_3002 | JN902586 | F:CAAATTGTCCTTCCTCAGCAGC  R:GTAACGCAGACCAGAAGGTATTCA | 57.5 |
| scaffold1289_49994 | JN902587 | F:CCAGCTGTACTGCAGTTCCATTTT  R:GCAAAACTGGAGGATTTGGTTTG | 57.5 |
| scaffold444_21127 | JN902588 | F:CCTGCGACAGCTGAAATGATAGA  R:TGCTGCTGTAATGATTCCTCGTT | 57.5 |
| scaffold1394_52779 | JN902589 | F:GAGCAGGATTTATGAGGCCATTACA  R:TTCAGTGACCTGGTCCGTC | 57.5 |
| scaffold710_32178 | JN902590 | F:GCACCTTATCGTTCCGTTGACT  R:TGCTGTCTGCTTGTCTGCA | 57.5 |
| scaffold7687_71823 | JN902591 | F:AACGATGACAGGATCTTCATCTCC  R:TTTCTCTTCTTACACGGCCAGTTC | 57.5 |
| scaffold7687_71824 | JN902592 | F:AGGCCGACACAGTTTACATTGATT  R:TGAGGACAGTTGGAGACATCGTTA | 57.5 |
| Scaffold074865 | JN902593 | F:TCTTGGCATCTACGCTCCATT  R:AGATGTGTCCAGAGTGTCAACTC | 57.5 |
| scaffold2336_66164 | JN902594 | F:TGTGCAGTATTTGAGCGTGTG  R:GGTGAGGGTGAGGTGAGATT | 57.5 |
| scaffold191_10459 | JN902595 | F:GTTGAGTTTGGTTATGCTGCACTG  R:CAGGGTTCAATTCCCTCTGTG | 57.5 |
| scaffold7652_71820 | JN902596 | F:TTACTGCCCTCTGCTGTCTGT  R:GCATGCAGCAAGTCCTAGGTT | 57.5 |
| scaffold969_41193 | JN902597 | F:TTAGATGTGATGGGCACTGGAG  R:AGTCGGATCAGGTGAAAACTGTG | 57.5 |
| scaffold3330_70410 | JN902598 | F:GCAGGGAGTGACCATGTCTAAA  R:CCTGTTTCACTCTGCAGCTAAC | 57.5 |
| scaffold46_1826 | JN902599 | F:GACCACTACCTGGGCAAAC  R:TGCGATCACCTGAACATGACA | 57.5 |
| scaffold3281_990 | JN902600 | F:TTCACGCGGTCACATGCA  R:GCTGCTGTTTCATCACCTCCA | 57.5 |
| scaffold1535_55453 | JN902601 | F:TGTACACATACATACAGGTCGTTCAA  R:CCAGGGCCTGATACCAACAT | 57.5 |
| scaffold413_20262 | JN902602 | F:TTCCATGTCTTGGCAGACACT  R:GCAGAATGGAGAAAACAGGCAAAT | 57.5 |
| scaffold1496_54674 | JN902603 | F:ACACACAGGAAAGACCAAAGCA  R:TCAACCTGGACACCTCTGAAAAG | 57.5 |
| scaffold2267_65927 | JN902604 | F:TCTTTGTCTGTGCCTCCCTG  R:ACAGAATGGCTTTGCAGTGGT | 57.5 |
| scaffold1867_60868 | JN902605 | F:CACCCGTGTACTGAGCACT  R:AACGATGTCTGTGTAGGAGGTTTAAC | 57.5 |
| scaffold1528_55393 | JN902606 | F:CCAATCGGCTTCAGAGATTTGGA  R:CACACATGCTCGGTCTGTTTC | 57.5 |
| Scaffold076312 | JN902607 | F:CCAGGCTCTGTTGTTTGAGG  R:GCAGTTTGGGCATAGTTCCAG | 57.5 |
| scaffold3805_71274 | JN902608 | F:ATTGCTTTTTCAAAGCGACCTCAC  R:CCACACGTATTGCCAGCTTATC | 57.5 |
| scaffold1129_45153 | JN902609 | F:TCTGCTGGTTCGGAGATTCC  R:GCAACTGTGTGTTTCATTTGCTTG | 57.5 |
| scaffold3805_71275 | JN902610 | F:GGATGAGTTCGGTGTTTGAACAAG  R:CCTGTTGATTTCAGGAAAAGATAATGA | 57.5 |
| Scaffold077693 | JN902611 | F:GTGCTCACACAGGATGCTC  R:TGTTGGGCTACTCCAACTTCAG | 57.5 |
| scaffold1867_60846 | JN902612 | F:TACTGAAGCGCAACTCATGGT  R:TACCTCCAACGGTTTCAGTCC | 57.5 |
| scaffold2227_65510 | JN902613 | F:CTGTGAGCCCCTGCAGTTAT  R:ACGTCCTGCTTCCATATCATTCC | 57.5 |
| scaffold927_25938 | JN902614 | F:TTTGCTCTGTAGCCCTGACA  R:GAGCAGAATTTGCCTGGAGAG | 57.5 |
| scaffold346_17345 | JN902615 | F:GCCCGCATGAAAGTATGCAATTA  R:TTGCAATCATTCTGATGGCACTG | 57.5 |
| scaffold505_24900 | JN902616 | F:CACGCCAGTGAACAAAGACAC  R:CTCCCCTCTCAAACTGTCCAA | 57.5 |
| Scaffold000136_672 | JN902617 | F:ACATCAGCTGGAACACCAGT  R:ACAGCGGGTCTTGTCCATTAT | 57.5 |
| scaffold3538_70710 | JN902618 | F:TACAGTGGCACATCGCCTAC  R:ACACTGTGGCAAAAGACAGC | 57.5 |
| scaffold3931_70955 | JN902619 | F:CTGATTTGGATTGACAGACGACAC  R:ACGACACTTTATGACGGCGTTTAT | 57.5 |
| scaffold6507_71698 | JN902620 | F:TGTGGAGGTCTTAGACATACGGGT  R:AGAGAATGTTGGGGACTTTTGTGA | 57.5 |
| scaffold583_19055 | JN902621 | F:AGACCTTGGTGTGCTGATGT  R:AGCATCTTCACCTGCTTGC | 57.5 |
| scaffold2679_68850 | JN902622 | F:ACTCCAAAAGGGAAATCCCAAGT  R:AAATGCCACATTCCTGGCAAAAA | 57.5 |
| scaffold317_16808 | JN902623 | F:TGGGGGAAACAGGAAGTTAAGAG  R:ACGACGAACCCAACGTGATT | 57.5 |
| scaffold317_16838 | JN902624 | F:GCACGCTTAAACAAGCTGAAGAG  R:TCACCCTGTGAAGACAGTTAAGTCA | 57.5 |
| Scaffold076174 | JN902625 | F:CCTGGTAGTTGTTGCAGATGATTG  R:GTGCTCCAGCCAGTAAACC | 57.5 |
| scaffold1660_58045 | JN902626 | F:GGGAGGTGTAGCTTCTCCAAAA  R:AGGCAGAGCAGGTGTAGTTTC | 57.5 |
| scaffold1785_59319 | JN902627 | F:GTGAGTGTTGTTGTGTTTGGAAGC  R:TTCACATTCCTCCCTTTACTCGTC | 57.5 |
| scaffold2755_69081 | JN902628 | F:GAGAGTCTGAGTGAGGCGTC  R:TGGCTACTCTACCCATAGTTGTCA | 57.5 |
| Scaffold000334 | JN902629 | F:TCAGGGCTGAAATACCTGACG  R:CGGACACTCTTCCCTCACA | 57.5 |
| scaffold2079_63850 | JN902630 | F:TCTAGACGGAGGAGGACAGG  R:GCGACGGAATCTGAAAACACG | 57.5 |
| scaffold5512_71554 | JN902631 | F:GGAACATCCAAGACCAAGAAGAAA  R:ATGGAGACGCTGAGGAGATTAGC | 57.5 |
| scaffold1784_59253 | JN902632 | F:TGGTCTGCACTGAGGAATTTGT  R:TGGTTTGAGTGCAGGACAACTT | 57.5 |
| scaffold782_35153 | JN902633 | F:CTCACTCACTCACTGGCTGAT  R:TGCTCAGGGCTAAAGAGTGTTG | 57.5 |
| scaffold1816_59732 | JN902634 | F:CTCAGGGTGACTGCTCACAT  R:CGTTGGTTCGCCTTGGATTTTAC | 57.5 |
| scaffold3647_70722 | JN902635 | F:CCACAGCCTATTCTGCTGGA  R:GACCGTCTTCTAAAGACTACATCACAG | 57.5 |
| scaffold7854_71905 | JN902636 | F:GTTTAGACGCACAGGAAACCAAG  R:TAACGACGGGTCGGTGTTATCT | 57.5 |
| scaffold3908_70949 | JN902637 | F:TCTCACCCTCCATTGGAAATCA  R:TGTGGCTGTTTAAAGCGAGGT | 57.5 |
| scaffold113_5940 | JN902638 | F:CACCACGTCTCACCTCTTCA  R:GAGAGGGAAACCCCATGTCA | 57.5 |
| scaffold51_1364 | JN902639 | F:TCATCTCAGGTGCAAACACACA  R:GCTCTGGCTTTCCTGGACTTA | 57.5 |
| scaffold255_9681 | JN902640 | F:AGGAGAACAGGTCAGTCATACGA  R:CTCGTTCCAAACTCTCCTCCA | 57.5 |
| scaffold4783_71433 | JN902641 | F:TGAATCTACCAGGCCTAGAATCAGA  R:ACATTTAAGCTAACCGGACTGGG | 57.5 |
| scaffold1549_55680 | JN902642 | F:GTTAAAGGGAGCCATTCTGTGC  R:GAGCTATTGATTGAGTCAGTGGTGA | 57.5 |
| scaffold667_31416 | JN902643 | F:GGTTCACGACGGAAGCTTTACT  R:AGGTTTGCCTCTCCAACATCTAC | 57.5 |
| scaffold224_10839 | JN902644 | F:CCTCTCCTGCAGTGCTGTTTT  R:GACCGCTCGGAGCTTCTT | 57.5 |
| scaffold377_18985 | JN902645 | F:AGACAAAACAGCGGTGGATGT  R:CCTGCATGTGCTGTGGTTTTAA | 57.5 |
| scaffold1211_47503 | JN902646 | F:TGTAGCAAAGTTGGCCTGTGA  R:CTGCCAATTATCCCTTCCTCCT | 57.5 |
| scaffold1829_59726 | JN902647 | F:ACTCAGTCCACGTCCCAGT  R:ACGGAGAGACGTTACCTCGAA | 57.5 |
| scaffold771_34966 | JN902648 | F:CAGCTGCAGAACCAAACAGAG  R:CCACAACCTGGACACATCAGA | 57.5 |
| scaffold452_22891 | JN902649 | F:CTGCTCGACTGAAAGCCAC  R:GTTGCCAATTCCCTACAGCTAAC | 57.5 |
| scaffold856_37844 | JN902650 | F:CTCGGCCATGGGTAACATG  R:CATCTCATTGGCAGACGGATTTTAGT | 57.5 |
| scaffold292_14438 | JN902651 | F:AGGGAAAGCAGCAGATAGGAGA  R:TCAGTGACAGCACTCAAGGTG | 57.5 |
| scaffold966_40257 | JN902652 | F:TCATCTCTCTCGGAGGAATCTAAGTG  R:ACCTGAAGCCTAATCTGACTCGA | 57.5 |
| scaffold2391_66683 | JN902653 | F:TGGTATTCCTCTCCCCCTCTC  R:GTGGGGTCAGTGGTGAGATTT | 57.5 |
| scaffold844_23968 | JN902654 | F:CTGACAGATACAGCAAGCAGGA  R:AGATGGACCGAGGGTGTATG | 57.5 |
| Scaffold077882 | JN902655 | F:TCCTCAGGAAAGGAGGAAACC  R:GAACACGGTGGGTAAATGTCAG | 57.5 |
| scaffold2725_68750 | JN902656 | F:GTGCATGGAACTGTGTCTGGA  R:AGCTGAGGCCTGTAACTGTCG | 57.5 |
| scaffold434_20553 | JN902657 | F:TGCTAACATCTCCCTCTGCAC  R:GTAACCCACCCATCAACGAACTA | 57.5 |
| scaffold5709_71596 | JN902658 | F:TCACGTGACCAGTTTGAGTCTTGT  R:TAGAATTCACTTGGAGGCGTTCTC | 57.5 |
| scaffold82_3397 | JN902659 | F:CCACCCTAAACTTGGAAACCG  R:CGTCTGCACTCACCTCAGAA | 57.5 |
| scaffold2135_64163 | JN902660 | F:GCTGAGGGAGTCACCTGAT  R:TCCTCCTCTGTCTGAGATGCA | 57.5 |
| scaffold923_38926 | JN902661 | F:TCCTCCATCCTTCATTTTGTCCC  R:TCTGAAGTCCCATCATGCATCTG | 57.5 |
| scaffold1446_53855 | JN902662 | F:GACGTGTAGCCTGGAAGTA  R:CCACAGTGTTGTTGACACA | 57.5 |
| scaffold1889_61211 | JN902663 | F:AGTGAACTGACAAATCCACGACAG  R:CAGGGCATTTGGACAGAGG | 57.5 |
| scaffold2114_63925 | JN902664 | F:GGACCACGTGCAGAAACTG  R:GACACGTTCTGGCTGTTGG | 57.5 |
| Scaffold080440 | JN902665 | F:TCCAGTGTGGCTACAGTAAACAC  R:CGGATGACTTTAGGCAACAGG | 57.5 |
| scaffold1278_49686 | JN902666 | F:ACATTCACTCACGTTCACACAA  R:GCAAACATGACGCTCTAATCC | 57.5 |
| scaffold4604_71363 | JN902667 | F:GATGACCTGCCCTGTATTTGC  R:CGTAATTGCTTACGCCTGTAGTGT | 57.5 |
| Scaffold074268 | JN902668 | F:GATGTGCTGAGGCACTCTG  R:GGATGCAAGCTCACTATCTCACA | 57.5 |
| scaffold442_21206 | JN902669 | F:ACCTGGGAAATTACACCACAAACA  R:GAAGAGGAGTCCAAGCAACAGT | 57.5 |
| scaffold1894_61818 | JN902670 | F:CATTTTGACCACAGCAATTTCAGCT  R:GTCATTGTCATCACTGGAAAAAGCAG | 57.5 |
| scaffold1069_43596 | JN902671 | F:TCCACATAAAGTGCTCCCAAGAC  R:TGGATGAGGTGCAGATTAATGGTG | 57.5 |
| scaffold1337_51158 | JN902672 | F:GGAGACCAATGTGCCAGATGA  R:AATTGTCAACTGTCTGGCAGAGAAA | 57.5 |
| scaffold368_18530 | JN902673 | F:AAACAACTGAATTTGCCCTTTTGA  R:TCATAGTTGTGCTTTTCTTTGGGG | 57.5 |
| scaffold8103_71897 | JN902674 | F:TGTTTGTGTGAGCTTGTGCATG  R:AGACTCTGGACTTTGATGTTCTGC | 57.5 |
| Scaffold000025 | JN902675 | F:GAGGTCAAGAAGCCCTTTGG  R:CGTTCTGGCACTGACTGTG | 57.5 |
| scaffold1685_58230 | JN902676 | F:TGGACAGCACTGGGATTTTCT  R:ACAGGTGGTCACAGGACTG | 57.5 |
| scaffold645_30065 | JN902677 | F:AGGACGTTGACAGGTTGGTGT  R:ACAGGAGATCAGCAGGGATCA | 57.5 |
| Scaffold000249_675 | JN902678 | F:CATCTTTCAACGTATCGACGGC  R:AATGCAGCGTCTGTACCAGT | 57.5 |
| scaffold1901_61969 | JN902679 | F:GTGTGTCTTTACCATCGCGTG  R:CTGCTTTCGTGACTGAAGGATG | 57.5 |
| scaffold3769_70902 | JN902680 | F:CAGCAAGCATGTAGCCTTAGC  R:CAGTGCTGTTGTTCGGCAAA | 57.5 |
| scaffold2791_69083 | JN902681 | F:TTTAATAAAAGTCCACAAATGAAGGAA  R:TTGGCTTTAAAGATTTTGTCTTCAG | 57.5 |
| scaffold367_19332 | JN902682 | F:GAGGGGACGTGGTGATAAAGAC  R:GAGATTGAAGCCGTGCTGAC | 57.5 |
| scaffold90_4658 | JN902683 | F:GTGCATGTGTCGTACAGGAGT  R:ACTGTGACACTGCATGGTGAT | 57.5 |
| scaffold1189_46715 | JN902684 | F:GGAAGCGTCACACTGGTGTT  R:TGGGCCTATAATCTGGGTCATGT | 57.5 |
| scaffold117_6029 | JN902685 | F:GACGTGAACACAGACAGCATG  R:TCTGTAGGCATGAGGACCATTG | 57.5 |
| scaffold154_8031 | JN902686 | F:TGAAATCCACTCGATCTGCCATC  R:TTCGTGTTCACAAAAGTGCTTCC | 57.5 |
| scaffold237_8580 | JN902687 | F:ACATCAACCATCAGCCAGGA  R:CATGAAGTGCGTGGACAACA | 57.5 |
| scaffold1435_53546 | JN902688 | F:TCTTACACCATATCCCTGCTC  R:AGGAATCCAGGCCACTAGA | 57.5 |
| Scaffold075582 | JN902689 | F:GTCTCTGCCCTTCTTATTCACGT  R:AGGCAGCAGGGCTAATGA | 57.5 |
| Scaffold000044 | JN902690 | F:TGCTGCTGCTGACGTATGA  R:CCAGGGCTTTGCTTTAGAGTTG | 57.5 |
| scaffold1410_53118 | JN902691 | F:AACGTGGAAATAAAGCAGTTTTGC  R:GACTGACATACCGTACACTGCCTG | 57.5 |
| scaffold177_8916 | JN902692 | F:CCACTCCATCCTCAACACTTCT  R:GAGCAAGTCTGTGGGTCTGT | 57.5 |
| scaffold2250_65597 | JN902693 | F:GTCAGCCTTGTCACAGTCCT  R:CCCCTCCTTTCTCTTCCAAATCA | 57.5 |
| scaffold2250_65588 | JN902694 | F:GCCTCTTTCAGAGGATTGCCTA  R:CCTTTAACCCAAACGGCTACAGA | 57.5 |
| scaffold251_12407 | JN902695 | F:GCCCTGTATGATCGAGTCTCTG  R:TCCAGACGAACTTCTTGTCCTTG | 57.5 |
| scaffold775_34737 | JN902696 | F:GCTAAAAGTCTGTGTTTGTATGAGC  R:GTTACACAGGCACATGAATGC | 57.5 |
| scaffold4442_71330 | JN902697 | F:AGATCAAAATAAGACGCTGCATGG  R:CCGTGATCATTTAAACCCTTCATC | 57.5 |
| Scaffold000344 | JN902698 | F:GCTCCGGCTAAACAAAAGCA  R:CTCAAAGCCCAGGTGTGATC | 57.5 |
| scaffold3335_71053 | JN902699 | F:GCCCTACCATTATCCCTCTGTC  R:GCAGCAGAGAGGTTACAGCT | 57.5 |
| Scaffold000030_473 | JN902700 | F:TTGATGGAGTTTATCGCCTGGT  R:CAGGCTGAACAAAGAGGAGGA | 57.5 |
| scaffold1581_56746 | JN902701 | F:CTGAGGAAACACCGGCAACAGTG  R:GAGAGCAAGCCATCTGTGCTCCT | 57.5 |
| scaffold3057_69884 | JN902702 | F:CAGGGATATGACGAATGAATGAAGT  R:AAGGGGTGTTGTGTTTCCAAATG | 57.5 |
| scaffold2233_65380 | JN902703 | F:TGACTCTACCACAGGCTTGGA  R:GAAGCACTTTTCGTCCTCCAGT | 57.5 |
| scaffold589_18323 | JN902704 | F:TCCGTCACACGGATCAATAGG  R:ACATCAGCCCTTCGACGTAAA | 57.5 |
| Scaffold000003 | JN902705 | F:CTTCAGCGCCTCCATCAAC  R:ACGGAGCCCAGTTTGACT | 57.5 |
| scaffold1542_55459 | JN902706 | F:AGGTGGGAGACTCATGAGGA  R:GTTTTGTCATAGCCAACTCAGCC | 57.5 |
| scaffold2714_68695 | JN902707 | F:TCCACTCTCTTGGCTCGTGTA  R:TTCCTGGTTAAGAGCAGGCTTC | 57.5 |
| scaffold697_31679 | JN902708 | F:TCCTGACCCGTGAATACTCTGA  R:GACGGATAAACAGCTGCCAAAG | 57.5 |
| scaffold7735_71828 | JN902709 | F:GTCCTAGTCCTAGTCCTAATCCTAATCC  R:GGTTGAATTTGACTGAATGTGATGGAG | 57.5 |
| scaffold2366_737 | JN902710 | F:ACCCAGGAGCCCACTCATA  R:AAAACCAGCTGAAGGTCAACGTAC | 57.5 |
| scaffold1932_62165 | JN902711 | F:GATTGGACCATGCTGACATTCATG  R:GGCTCTGGCAGTATGTGAAATG | 57.5 |
| scaffold2231_65418 | JN902712 | F:GAAACGAGTCCTTCTCTCAGCA  R:GGATGGAGGAGTAGAACGAGG | 57.5 |
| Scaffold000320 | JN902713 | F:GACGTCACATGATCTCAGTGCT  R:GATTGGTCAGACCTCAGTGCT | 57.5 |
| scaffold2778_859 | JN902714 | F:ACGCTGTGGAGACAATTACAGACA  R:GCTGAATGAACTGCCTCATTGGA | 57.5 |
| Scaffold080425 | JN902715 | F:GCAGCCAGTTCTCAGTAGC  R:GGACTGCCGTGACATCAC | 57.5 |
| scaffold3116_70372 | JN902716 | F:GCAAGGCTGCATAAATGAGCTATTT  R:CCCGTTTTAAACACCAATAAAACGTCA | 57.5 |
| scaffold48_1266 | JN902717 | F:GTGAACCTGTTTCGGTGAAGTG  R:GTATGAAGTAAAGCTGCGCGTTC | 57.5 |
| scaffold3116_70379 | JN902718 | F:CAGCTTTCTGCCGAGTAAAGAC  R:TGAATACTTGTTATCAGGGTTTTCTGGTG | 57.5 |
| Scaffold076014 | JN902719 | F:CCACAGCATGTTTGACGTGT  R:CATGATCGACGACAGATGAACGA | 57.5 |
| scaffold4228_71185 | JN902720 | F:ATTCATCAGCCGTCCATACTGAG  R:TCCCCCAAAGTCTTGTTCTCTC | 57.5 |
| scaffold2735_68897 | JN902721 | F:GGTAACCTACTAGTTCACCCT  R:ACTCAAGTGTCCTTCATCAGT | 57.5 |
| scaffold399_19427 | JN902722 | F:CGGGGGTGCAGTAAACTCT  R:CGGTCACAGCAGCAGTAAC | 57.5 |
| scaffold1644_57993 | JN902723 | F:AGCTGGAGCTACCACTACCT  R:CGGACCATGCACGTATTGAAC | 57.5 |
| scaffold1557_55702 | JN902724 | F:GCTTCGGTTCACGTTCCCTA  R:GGAACCGATCCAGAGATGATTTGA | 57.5 |
| scaffold565_27838 | JN902725 | F:TTGTCATTTCACCTGCTCACCT  R:ACCACCGTGACCTACTGTC | 57.5 |
| scaffold1777_59215 | JN902726 | F:CGATGATCGAGTCCTGAAGGTG  R:CGGACGTTTCCACTTCCTCTAA | 57.5 |
| scaffold1777_59208 | JN902727 | F:AAGCCCTGTGTTGTTAATGACCA  R:CGTCCTTTGTTCCAAACATCCAG | 57.5 |
| scaffold850_36934 | JN902728 | F:CTAGTTAAGGGCAAGGACTTGCAT  R:TTGCAGAGAGATGCTGAATGGTT | 57.5 |
| scaffold1227_48418 | JN902729 | F:TGGAGACCTTTCAAGGCTTTTGT  R:ACTGCCTGCATCACATTCTCT | 57.5 |
| scaffold1363_51755 | JN902730 | F:GCAACTGTTGGGAGTTTTTGGT  R:TCCTCCCTTTCCTCCTTCTCT | 57.5 |
| Scaffold074671 | JN902731 | F:TTCACTGACTTGCACTGGGT  R:AAAGTTCACAGTGGGCGATTG | 57.5 |
| scaffold1526_55284 | JN902732 | F:ACCCGCATGACTAACTCCATTT  R:ATTGATCAGCCTCCGTCCTTAG | 57.5 |
| Scaffold074339 | JN902733 | F:TGAACACCACGGTTGTCAGT  R:TCATCTGTGAACACCTCCACTT | 57.5 |
| Scaffold000084 | JN902734 | F:CAAGGCCATCTGTCACCATG  R:CTGTCTGGGACTCTGGGTTT | 57.5 |
| Scaffold076143 | JN902735 | F:ATGGTACGCTGAGTCCTCG  R:ACCTGGACTCCCTTCAGAAAC | 57.5 |
| scaffold1788_60032 | JN902736 | F:GTCTTCATCCAGAACATGTGGC  R:CATTTGAGAGAAGACAGACATCAATGGT | 57.5 |
| scaffold1788_60033 | JN902737 | F:GCTGTGCTTTGTCTGCTGTT  R:AGACACCTGAAACTGTCTGCTG | 57.5 |
| scaffold1102_44436 | JN902738 | F:TCCTGTGTCCACAAAGAGTGG  R:GCGCACACATACTCCAAACTTC | 57.5 |
| scaffold198_9634 | JN902739 | F:CTTTTGTCTGCAGTGGGATCAG  R:CTGGAAGCCAGGACGGAATTAT | 57.5 |
| scaffold7277_1163 | JN902740 | F:TTCAGAAACCTCCAGCTCGTCT  R:TGGTTTGAATGGGAGTTCTGGTTG | 57.5 |
| scaffold1291_50712 | JN902741 | F:GCTGGTGATGGGAGAGAAAACT  R:AAAAGCCCCCTGTGTTTGG | 57.5 |
| Scaffold000225 | JN902742 | F:GACATTATCCGTGAAACAGCCG  R:ACTACAACAACCACCCCCTT | 57.5 |
| scaffold4451_1100 | JN902743 | F:CTGACGACCAGAGAACAGA  R:CTGTTGGACGTCATTGAACAT | 57.5 |
| scaffold7321_1165 | JN902744 | F:CAATGGGAGAGGAGACATTTCTGC  R:CCTCATAAAGCAGACCATTGTTGGT | 57.5 |
| scaffold814_35282 | JN902745 | F:GGTCTCTCTGGACGTTGCT  R:CCCCCGTCCTGTAATTGACA | 57.5 |
| scaffold845_36318 | JN902746 | F:CACATCCCGGGTCATTTCATCTA  R:TGTGGCAGAGTCACACCTG | 57.5 |
| scaffold479_23523 | JN902747 | F:CAGTGCCGTGTTTCTCCTG  R:GAACCTGAAGGGGATGTTGGA | 57.5 |
| scaffold3429_70488 | JN902748 | F:AGCTCCTCATCCAGCTTCAAAC  R:CAGTGCTAACAGGGACAGCTT | 57.5 |
| scaffold4658_71338 | JN902749 | F:TACTGGCAAGGTCAGAGCTG  R:GTTGCACAGCCTTCATCAGG | 57.5 |
| scaffold414_19940 | JN902750 | F:GGCTGTTAATCTGGTCACA  R:CAGATAATTGGTCCCCTGAA | 57.5 |
| Scaffold080437 | JN902751 | F:GACTGCAGCGTAAATCTGAACAC  R:TGGAATCACTGCCAAGGTGTA | 57.5 |
| Scaffold078839 | JN902752 | F:CCGCGTTTGGAGTGTAGAAG  R:GGCTTGTGGAAGATCCAATCAG | 57.5 |
| scaffold1240_49281 | JN902753 | F:GAGGAGAGACTGGCTGAATAGG  R:TGTCCTACAGCAAGAATGGCAAA | 57.5 |
| scaffold4475_71287 | JN902754 | F:CCTGAGGGACAAAGTACATGC  R:GGAGTGTGAGGTAATGACAACG | 57.5 |
| scaffold1054_43419 | JN902755 | F:TGACACAGACTAATGGGCTCTG  R:TCTTCTGAGCCCCTCTTCATTTC | 57.5 |
| scaffold1695_58349 | JN902756 | F:TCCAGGTTTGGGACCAATTACAT  R:CCCAGCGTGGTCTCATTTACTA | 57.5 |
| scaffold2495_67575 | JN902757 | F:GATCTCACTCTGTCCCCTTCTG  R:ACGCACACATACAAAGTGATGGA | 57.5 |
| scaffold2063_63713 | JN902758 | F:ATGACTCAGCAGCCCTTCAAA  R:GTTGGAGGTGGCCAAGATAGTT | 57.5 |
| scaffold2632_68357 | JN902759 | F:CAACACAGGCGGTTTGATGAC  R:GACAGACCCTGTCCCTCTAATTG | 57.5 |
| scaffold1032_42172 | JN902760 | F:CTCTGTGACCCCGTTAGATTCC  R:AACGCTCACATTCACAGACGTT | 57.5 |
| scaffold86_3568 | JN902761 | F:ATATCGCTCCCCTTCTGTCAAAC  R:CGCTGCCTGACATATACACACA | 57.5 |
| scaffold547_25905 | JN902762 | F:ATGGAGAGTCACGAGACATGC  R:CTAGGGGGTGCTCAAGACAT | 57.5 |
| scaffold3198_70312 | JN902763 | F:GATCACTGCATCCTAACCCAGC  R:GCTTCTATGGCAAGTCAAAGGGA | 57.5 |
| scaffold2065_63431 | JN902764 | F:CGCAGTGAGGACATACGTGAA  R:ATCATGATGGAGAACAGGAGCAAAA | 57.5 |
| scaffold93_3826 | JN902765 | F:GTGGTCTGCCTGTTAGCTG  R:GCAGAATGGCAGTGACGTAC | 57.5 |
| scaffold2058_63242 | JN902766 | F:GACACACGAATCTGCAGGA  R:AAGGGCTCATTTAGGCTTCA | 57.5 |
| scaffold2058_63244 | JN902767 | F:GCTGGACAGTGAATGACGT  R:CGACCAAAGGGTGGAAGTAA | 57.5 |
| scaffold3355_70439 | JN902768 | F:CGTTCCGTAGGAAAACAAAGACAG  R:TGGGTAATTACGGTTATGGAGGTG | 57.5 |
| scaffold1213_48105 | JN902769 | F:AACCACTGCATAAAACGGACAAAC  R:GAAGCATCATCTGGCCCAAC | 57.5 |
| scaffold2068_63627 | JN902770 | F:ACCACTTCTTCTGGGTCTG  R:CCCTGAGGGAAATTGTTGAC | 57.5 |
| scaffold1665_58305 | JN902771 | F:CCAATTACGTCTCACCGACCAA  R:ACGCCGTGTGTGCATTTG | 57.5 |
| scaffold1665_58308 | JN902772 | F:TCACAGAACTCACGCAGGAC  R:CACAGTGACGACCAAAAACAAACA | 57.5 |
| scaffold532_25089 | JN902773 | F:GTGGATTTGAGGGTTAATCTGTGG  R:AAAAAGGGGCAAAAGGCAGAT | 57.5 |
| scaffold2068_63631 | JN902774 | F:TAGGAAGGGTTGTCCACAAG  R:ATCCCAGCAAGCAATTTCAC | 57.5 |
| scaffold1170_46055 | JN902775 | F:ACGCCACAGCTGATTCATTAACT  R:CTGTGCTGTCACTCCGAGAT | 57.5 |
| scaffold2841_69827 | JN902776 | F:TGCTGCAGTGAGGGTTTCAC  R:CCAGAAAACACTGGCAGCTCT | 57.5 |
| scaffold138_8327 | JN902777 | F:GTCGCTACAGTCAATGGGGTA  R:TTTCTCTCACGGGATGTATGCTC | 57.5 |
| scaffold1118_45406 | JN902778 | F:GTCACAGGCAAGAAGTGCAG  R:AGTGAAAGAAATAGGCACGGACAG | 57.5 |
| scaffold94_5336 | JN902779 | F:ATAACCTGCCGTGACAAGCAT  R:GATTTGTGGTCCAGGTGACAGT | 57.5 |
| scaffold556_26255 | JN902780 | F:TGTCCACTTCATGGCCTAATGG  R:GTCAGATTCAGTTGCCTCCGTA | 57.5 |
| Scaffold000098 | JN902781 | F:TTTTAGCCCCAACAACGGTG  R:TGGATAACATCACCTAGAGCTGAAC | 57.5 |
| scaffold556_26262 | JN902782 | F:GGGCCTTAATGGTTCCAAAACC  R:CCGTAGCAAGGTGAGCTGT | 57.5 |
| scaffold1865_60496 | JN902783 | F:TTTCCATATCTCCTGTGAGATCGAAGA  R:TTTCTGTGTGACAGGCCAAAGT | 57.5 |
| scaffold1461_54027 | JN902784 | F:GTGGTGTGGGGTTCGATAGTTT  R:TCTCCATCCACTCGAAAACAGG | 57.5 |
| scaffold72_2796 | JN902785 | F:TAAACTGCAGCCGCTAAAAGC  R:TCGCCTGTGGTGAAAACACT | 57.5 |
| scaffold169_8279 | JN902786 | F:AGGATCCACATGGGAGATGG  R:CCACGTGGTTTCCAGTCATTTT | 57.5 |
| scaffold169_8277 | JN902787 | F:TGCCTCTGTGACTCCGTTC  R:TCGCCCTCCTTTCTCAGTC | 57.5 |
| scaffold1677_58093 | JN902788 | F:GCCGTACGTTTGGTTAAAGGAC  R:CCTTCATTGTTCCCTTGTTTCCTTC | 57.5 |
| scaffold1876_60931 | JN902789 | F:GAGATTTGCCTCAGCGAGAGA  R:TAGAGGGAGGCTTAGGAGTGG | 57.5 |
| scaffold296_15116 | JN902790 | F:CGCGAGAGTTCTGTCAAATCAT  R:CACCTGTGGTCAATCACTCTG | 57.5 |
| scaffold6975_71737 | JN902791 | F:TCATCAATTACGGCCACAGA  R:AGCAGCCCACACTGTCATTT | 57.5 |
| scaffold1797_59428 | JN902792 | F:GGTAGAGTCAGCTTCACAGGTAC  R:GATTGTTGACCATGCAGCGATAAAC | 57.5 |
| scaffold637_29530 | JN902793 | F:GCTCACACAATGCTGACGTATG  R:AGGCAGTTTCGGACCAGATTTA | 57.5 |
| scaffold637_29532 | JN902794 | F:GCGCAAATAAAATTCCACATCCTCA  R:GATCGTTTCTCTCCTGGCAGTT | 57.5 |
| scaffold1643_57851 | JN902795 | F:AGCCAGAGTCTCACACATCG  R:GCGTGCTACAAAGAACAGACG | 57.5 |
| scaffold255_12500 | JN902796 | F:GTCCCGATTCCTGACACTTCTT  R:CGGCATGAGCAGCAAACTT | 57.5 |
| Scaffold075463 | JN902797 | F:CAGGTCAGTCAAGCATCCAC  R:TCCCATATGTCTGAACCACCATG | 57.5 |
| scaffold450_21180 | JN902798 | F:ATCATAGCTACGGTGGTAACCTG  R:TCTGCCACATTTACGACCAGA | 57.5 |
| scaffold822_35662 | JN902799 | F:CGCACAGCAGCCATTTATACTTC  R:CGTGAGAATGAAACACGTCTCCT | 57.5 |
| scaffold815_35701 | JN902800 | F:GATGCCCACGGACTGATCTAC  R:TTTCCCTCAGTGTTTGAGTGTGA | 57.5 |
| scaffold450_21173 | JN902801 | F:AGAGCCATAAGCTCCTCGA  R:CACCACTTCTGATACAGAGAGC | 57.5 |
| scaffold309_15574 | JN902802 | F:TGTAATTTGGGATGAAGCTGTTTGGT  R:GCTCCAGTCAGTCCAACAATG | 57.5 |
| scaffold754_33566 | JN902803 | F:GGCTGCCAACAACCTGTTTTAA  R:TGAAAGTCGGGGTCAGTTTCTAC | 57.5 |
| scaffold309_15553 | JN902804 | F:GAACACGGGCGATAAAATCTAAATGG  R:GAGCAAACGCTCTCATCTTCAAAC | 57.5 |
| scaffold7825_71863 | JN902805 | F:CACACTGCCTCAATCAGCATC  R:GTCTGAGGCGTTTTGTCTAACGA | 57.5 |
| scaffold4761_1107 | JN902806 | F:AAATTGACAGCCCAAAGAGGAAGG  R:CAGGGGATGGAGGATCTGATTTTG | 57.5 |
| scaffold1108_45079 | JN902807 | F:TCGGGTGTCATTTAGCCTGT  R:CTTCTGCCCTCTCTGGTCA | 57.5 |
| scaffold1153_47001 | JN902808 | F:TGTAGAAGCCAAAGCAGCTCA  R:TCACTGATGGTTTGTTCCTCCAG | 57.5 |
| scaffold2609_68329 | JN902809 | F:GCCCTGACACACGACTTAATGATA  R:CAACAATTAAAGCGAGGAGCGG | 57.5 |
| Scaffold978_40425 | JN902810 | F:GCGCACAGTAAACAGTCCG  R:ATTAGTACCGTTCCGCAGGAG | 57.5 |
| scaffold2001_62883 | JN902811 | F:GCTGTAGGACGACGACCCAGAGT  R:GAGTGTCCTTCAGGCTACAGCGG | 57.5 |
| scaffold2484_67905 | JN902812 | F:GTGTCACAACAGATCTCCAGC  R:CTCGTCTCCACAGGATGACA | 57.5 |
| scaffold604_29007 | JN902813 | F:CTAACAGTGAGATCCATTGGGT  R:TTGTGACCAACACGGACTTG | 57.5 |
| scaffold1232_48977 | JN902814 | F:TACCACTTCAACTTTCACAGA  R:TAGATGTGAGTCAGAGTCCA | 57.5 |
| scaffold1108_45058 | JN902815 | F:AGGTTGACTGTACTACTGACA  R:CTGAGGAGCCAGTGATTTC | 57.5 |
| scaffold641_30545 | JN902816 | F:GCACCACCTACTGTTTGACTGT  R:CTGCCATGTTCACCTCAGCTA | 57.5 |
| scaffold2694_68734 | JN902817 | F:TACCTGGACGCAGGCAATTAG  R:AGCAGCACTGTTTCAGGAAGG | 57.5 |
| scaffold1326_50968 | JN902818 | F:CACACACACGAAGAGCAAACA  R:GCTCTGATGTCACACTTAGAACG | 57.5 |
| scaffold6462_71684 | JN902819 | F:TGACAGCCTGGAGAAAACCTC  R:CATGGTGCCCCTGTAAGTGAT | 57.5 |
| scaffold99_5163 | JN902820 | F:CGGTGTAATTATGGGGCTGGT  R:TGTCGTCTCCCTCCTCTACTG | 57.5 |
| scaffold426_20511 | JN902821 | F:GGACATGTGATTCAGCAGCG  R:GCTCTCCGTCCATCCATCTATC | 57.5 |
| scaffold1347_51764 | JN902822 | F:ACCCGTTATCTCCAATGACAGC  R:TAGTCACGTCCGGAGATGTTTG | 57.5 |
| scaffold1569_56040 | JN902823 | F:GTCACACTAGTCCACAGAGGC  R:CGAGGGGTTCTGGATTATGCA | 57.5 |
| scaffold1961_62418 | JN902824 | F:CACATACAGGGCAAATGGAGAGA  R:GAAAGAGCATCATCAGGGGAGT | 57.5 |
| scaffold2430_67119 | JN902825 | F:AGGAACCAGTGATCCATACTGC  R:CTGTACTGGAGGGGCTACAAC | 57.5 |
| scaffold293_14390 | JN902826 | F:CCACCTGTAATGTGGAGGAACAT  R:ACACCAGAGTCAAAACCAGTTCA | 57.5 |
| scaffold1410_53106 | JN902827 | F:GCACATGTTGCAAAAGCTCATATT  R:CATCACCAAGAGTCAAACCAAGG | 57.5 |
| scaffold1405_52741 | JN902828 | F:ATTTGACCACTTTGTGCAGATTGA  R:AGTTACTTGAACACCTGACGGACC | 57.5 |
| scaffold1886_61068 | JN902829 | F:GTGAGGTGCATTCAGGGAAAC  R:AGACCAGTGCTAAGCTGACG | 57.5 |
| scaffold119_6091 | JN902830 | F:GAGGCTGAGGTTCTGAGTGAG  R:CCTGTTGTTTGGAAAAGTCGTGTG | 57.5 |
| scaffold1919_61948 | JN902831 | F:GAGCTTTTGATCCTGAACG  R:CAGAAGAATGAGAGAGTCGG | 57.5 |
| scaffold2094_64308 | JN902832 | F:CTGCAGTTTGTCCTCCAGTC  R:GGATAACAGGCTGGGGAATAC | 57.5 |
| scaffold917_38834 | JN902833 | F:GTCCTCAGGTTTCTGCCGA  R:AGAGTTGGTTTGGTGTCGCT | 57.5 |
| scaffold103_5044 | JN902834 | F:TATCATATTGTGTGCGCATGT  R:TGTGGCCTTTATGTCAAGTG | 57.5 |
| scaffold32_741 | JN902835 | F:CGTCCGTCTTCAGACCCTG  R:TGTCTGTGTTTCGCAGGGA | 57.5 |
| scaffold2084_63691 | JN902836 | F:CCGTCACTACAGTCTGTGTGAG  R:GGAGTCGGTGAAGAAACGCTATT | 57.5 |
| scaffold4399_71388 | JN902837 | F:TAACCAGTGTTGCACTAACAGAGG  R:TGGTGAGTCCACTGGAAGG | 57.5 |
| scaffold2622_68440 | JN902838 | F:GAGTCCAGTGCAAACACACC  R:CCGACAGCTCATGTAACCTCA | 57.5 |
| scaffold251_12419 | JN902839 | F:GCTGCTGGTCTTCCTATTGACT  R:GCAAATTCCAGTGCGAGCT | 57.5 |
| scaffold113_5945 | JN902840 | F:CAGAAGTTGGCGAGTCATCAGT  R:CATGTCTTTGAATGAAGGGATGGAGA | 57.5 |
| scaffold681_31972 | JN902841 | F:GTGGGTGGGCAGTAAGTATTAGTATG  R:CCGCAACCTCTTTTCTGTGAG | 57.5 |
| scaffold548_26593 | JN902842 | F:CATCTGTTTGGGAGCAGAGCT  R:ACTCCTGATGCCTCCTGGAATTA | 57.5 |
| scaffold709_32811 | JN902843 | F:GAGGAGGTGGACCAGAGAC  R:AGGTTTCCCAGTAGGGTCAG | 57.5 |
| scaffold1871_60636 | JN902844 | F:GAGGCTTGAGGTCGATTAACACT  R:GTCCCAACAGCTGAGACAGAT | 57.5 |
| scaffold2301_65872 | JN902845 | F:GAAAACATTGTGTCCCCGCATT  R:CGAGGTTGCACACTTTGTCTG | 57.5 |
| scaffold478_23084 | JN902846 | F:CGTTAGCTTCTTATGCAGCCC  R:GAGGCAACGTTCTACTGCAATTT | 57.5 |
| scaffold1484_54662 | JN902847 | F:CTGATTCAGCAGCCACTCTAATGT  R:GCTGTAGTGGTGTCTTTGGC | 57.5 |
| scaffold889_38452 | JN902848 | F:TCTCCACTGATCCTGAACCG  R:GACAGAACAGCAGCGTGAG | 57.5 |
| scaffold68_2610 | JN902849 | F:ATGCGGTGGGATGTTTGGA  R:GTAAGGAGCAGGCGTGTTCA | 57.5 |
| scaffold1013_41640 | JN902850 | F:CGTCATGCACGGAAGGGTTA  R:GCTGTGAGTATCGGTTTGACCA | 57.5 |
| scaffold400_19513 | JN902851 | F:GCTTGTTTGTGGGAGGAGATG  R:GACAGCCGGTGATGGGTAATA | 57.5 |
| scaffold170_8353 | JN902852 | F:AATACAAATGCACACACGGGAAGT  R:CCGGTGGAGGTTAAACACTGA | 57.5 |
| scaffold2729_68746 | JN902853 | F:ACCAAACCTATGACAGGGAGG  R:CAGCGTCACAAAGATGAACTCC | 57.5 |
| scaffold3045_69836 | JN902854 | F:AGCAGCAGCAGAAAGAACCT  R:TCCCCTCTGATAAAACCCCTCT | 57.5 |
| scaffold1582_56457 | JN902855 | F:CATGCTGACTCTCAGCCTCA  R:TGTCGTTCAAAGCCGTTCAAAG | 57.5 |
| scaffold849_37234 | JN902856 | F:CATGCTGACTCTCAGCCTCA  R:TGTCGTTCAAAGCCGTTCAAAG | 57.5 |
| scaffold15_965 | JN902857 | F:GAAGTGTTGTTCAAAGCGCAGAA  R:TAATGAGGCAGCAGGAGAGGTA | 57.5 |
| scaffold1853_60495 | JN902858 | F:ACACAAGAGTCCGATGAGCATG  R:TGACTTGTTTTATCGGCGTTTCCT | 57.5 |
| scaffold2111_63901 | JN902859 | F:TCACGGTAAACACTGAATATGACGG  R:GACCTTGGTGCGGGAGTTA | 57.5 |
| scaffold2798_863 | JN902860 | F:AGGTCGCTCTCCACCATCT  R:CTGAAGTACCTGCCGAGCATC | 57.5 |
| scaffold5398_1118 | JN902861 | F:CTGACCATGTCAACTGTGTTGTT  R:CCAGCGTTACATAGACAACTGC | 57.5 |
| scaffold600_132 | JN902862 | F:CTCCTGCCGCCGTATGTAAAT  R:GATTCCTCAGGGAACCTGGATC | 57.5 |
| scaffold7742_1173 | JN902863 | F:GCAGCTCACACGAGGGTATTT  R:TAAACCAGTGTGGACCGAGTCA | 57.5 |
| scaffold2540_796 | JN902864 | F:AGCTGTTAGCCAGGAGAAAGG  R:AGCTGTTAGCCAGGAGAAAGG | 57.5 |
| scaffold2393_764 | JN902865 | F:ATCAGAGTTGGTGCTGCCTC  R:CTGCCTTATAATCCTCCCTGCTG | 57.5 |
| scaffold464_22524 | JN902866 | F:CCTATGACCCCGATGACCAAG  R:TGACGTCCTTTCTTCCTGAGC | 57.5 |
| scaffold2585_68067 | JN902867 | F:GAGGGAGGAAGGCTAGTGAG  R:TGGAGGAAGTGTAAAGCCAAGG | 57.5 |
| scaffold314_15619 | JN902868 | F:CCCACGGGCCGTATGATATT  R:TGTCTCAGTGTGGAGATGCAG | 57.5 |
| scaffold4180_71193 | JN902869 | F:CAAAGACTTTTGCGGGTTAGCAA  R:CCTAGAGAGGACGTGGAGC | 57.5 |
| scaffold1285_50019 | JN902870 | F:CTGTGGCGCTATGCTCAATTAC  R:CGGTGTCTGTGGATCTGTTCT | 57.5 |
| scaffold2017_63119 | JN902871 | F:ATCAGGCTGGAGAACAGTACC  R:GAGGAGGAGGAGGAGGAGATAA | 57.5 |
| scaffold86_3546 | JN902872 | F:TGGGAGAGGGTTAAATAAAACTGACAAG  R:TATTCGCTAGAGGGCGCTAAAC | 57.5 |
| scaffold1021_42137 | JN902873 | F:CTCAGCCACCGTGAATAATTTGAC  R:AGCGAGTTTTTGGTTGAAAAGCAG | 57.5 |
| scaffold1865_60502 | JN902874 | F:TCAGTGATGGATGTGTGTCTGG  R:AACTTGAGCGTCTGAGCTTGT | 57.5 |
| scaffold310_15530 | JN902875 | F:AGTAGTGCACATTCCTTCATTTCTCAC  R:AGAGGAAGAAGGAGAACGAGGA | 57.5 |
| scaffold5060_71560 | JN902876 | F:CAATCAAGTGGCTTCACCAGTG  R:AACCAGGAGTCCCTTCAGG | 57.5 |
| scaffold2770_69061 | JN902877 | F:GTTGTGCTCCTGTGATGCAAAT  R:TGGACAACATCCACCTGTCTC | 57.5 |
| scaffold351_17591 | JN902878 | F:AACACAAGTCCAGGGCAAGAAT  R:ATACGGAGGCTGTTGAGGAC | 57.5 |
| scaffold1466_54398 | JN902879 | F:TTCATTTCCAGCAATTGACCAGC  R:GGCTGCATACCTGATGTGC | 57.5 |
| scaffold1894_61786 | JN902880 | F:ACCCAAGATGTGATGTGGTTTGT  R:TTTAGAGGTTGGGTTGCAGGT | 57.5 |
| Scaffold000136_345 | JN902881 | F:CTGCAGGTACAGTGCCTTCTA  R:ATCACCACAGTGACACACAGAG | 57.5 |
| Scaffold079995 | JN902882 | F:TCTCAGAGGAAAGTCTGCTGC  R:AGCTCCTCTTCAAAGAACCCTG | 57.5 |
| Scaffold000052 | JN902883 | F:ACAGCTGCTCCTGGAAATCAT  R:GCTGTCAGCTCTCTCACCA | 57.5 |
| Scaffold000121 | JN902884 | F:GAGAAGCTGCTCAAATGAACG  R:GAGAAGCTGCTCAAATGAACG | 57.5 |
| Scaffold000429 | JN902885 | F:ATTCACTGATGGTTGTCACCGA  R:AGCGTGCTCGTATCTCGAAG | 57.5 |
| Scaffold000087_137 | JN902886 | F:ACGGCTTGGTCAAAGAATCAGT  R:GATCACACAGCAGACGAAAACC | 57.5 |
| Scaffold000033 | JN902887 | F:CGTCTCTTCTCCTCTGTTGCT  R:TGTGTTTCTGTGTCCCTCTCTTG | 57.5 |
| Scaffold076901 | JN902888 | F:TTATCGCCATCGACAGTTAGACAA  R:TGCACTCCATGTTCTTCAAAAGG | 57.5 |
| Scaffold000360 | JN902889 | F:CTGAAGCTGGCTCAATTCTTTTTC  R:GTGTCAAACTGACATGTGCAACAG | 57.5 |
| Scaffold080427 | JN902890 | F:TGGAAGGAGATCAGCTCTGTG  R:TTTCTGCCCAATTGCTCCC | 57.5 |
| Scaffold074542 | JN902891 | F:TTCAAGCAGAGCAGTACACGT  R:AAAGACTCCAACGTGCTACGT | 57.5 |
| Scaffold080351 | JN902892 | F:GACTCTTCATCGACTGGGAGAC  R:GCTGGGCGATAGAGAGACAT | 57.5 |
| Scaffold080345 | JN902893 | F:GTTGATGACTGCTGGTGTGTC  R:CCACAGTACTGCCACCAAG | 57.5 |
| Scaffold000342 | JN902894 | F:CAGGTGTAGCCATGGTGTTG  R:CATCTGGTCAGAGCGGATCT | 57.5 |
| Scaffold077568 | JN902895 | F:GAGACTACGACCCAGGTAACTTG  R:TTGGGGCAAAACTCAGTTCAC | 57.5 |
| Scaffold080443 | JN902896 | F:TCTCGTCATGAGTTGGATGGAC  R:CTACGTGTGTGAGTGTGTTAGGA | 57.5 |
| Scaffold077077 | JN902897 | F:CTCAGAGTCGTGCTCTGTCA  R:CTTCACACTCCAGGGTCTGT | 57.5 |
| Scaffold075491 | JN902898 | F:GTCAGGAAGTCATTGAGGCG  R:CCTGCTGACATGAAGGGAC | 57.5 |
| Scaffold074718 | JN902899 | F:ACTGTCTGGTTCATCAGACCC  R:CCTACGGTTATGTCAGTCACAGT | 57.5 |
| Scaffold076622 | JN902900 | F:GCGTTCCAAGTGCTGATTGAT  R:TTCCCCATTCTTTAGCTGTTCAGA | 57.5 |
| Scaffold074874 | JN902901 | F:CCAGTAGATCACAAAGGGAGTGCT  R:TCTGTAAAACGGCTTTTATTTAGCCA | 57.5 |
| scaffold846_36023 | JN902902 | F:CACCTTGTGGTCCAAGATGAAATAGA  R:CTGAAGAATGCACCAGAACCAC | 57.5 |
| scaffold3110_70220 | JN902903 | F:CACCTGCGTACTCGTCTTCT  R:CCAGCATGCTCAGTTAGGC | 57.5 |
| scaffold490_23391 | JN902904 | F:TTACAGGACTGTGGTTGTCAGG  R:TTGTTTTGACTGGTGCTGGTCT | 57.5 |
| scaffold455_21462 | JN902905 | F:CGGCTGATTTCATCGTTTCTCC  R:TCTGAGGGCTGAATAACATGACG | 57.5 |
| scaffold5593_71599 | JN902906 | F:GGCAGAGAGTGTGACCGTTTA  R:TCCACCAGAGAGTTTCTCCTTCA | 57.5 |
| scaffold1263_49343 | JN902907 | F:GGCAGACAGCATGATAAACAGC  R:GGCAATGTGATGCTCCATCTG | 57.5 |
| scaffold1536_55732 | JN902908 | F:GGAGGGAGAGAAGATTCAGGTAGT  R:GGACGTCACTTTGAACGCC | 57.5 |
| scaffold114_4942 | JN902909 | F:TGTAACTGTGCCTCCACCA  R:AGACGCCGATGGTCAACA | 57.5 |
| scaffold276_9885 | JN902910 | F:AGTCTCCAGAAGCCACTGAG  R:CAGCGTAAACACACCTTTCCAG | 57.5 |
| scaffold421_14330 | JN902911 | F:GGTGACACGGCTCAGTTG  R:GGCTGAGGGACTCAGGAT | 57.5 |
| scaffold758_22695 | JN902912 | F:TCACTTCCTGACTCGTGTGTC  R:TTACTTGGGGGCGAATGGAT | 57.5 |
| scaffold900_25788 | JN902913 | F:TGAGAGCTGTGATTGGCTCA  R:TGAGGTTGTCTGCGGTTCT | 57.5 |
| scaffold1721_1835 | JN902914 | F:GGCTGTTGTGGATGTGTCTG  R:CCGTAGACCACAGTTTGAACC | 57.5 |
| scaffold20_1307 | JN902915 | F:CAACCGTGATCAATGAGTGTCC  R:AGGTGTCTTCTGAATGGAGAGC | 57.5 |
| scaffold1068_2577 | JN902916 | F:GCCACCACACCTGTTAAGAAA  R:GCCACCACACCTGTTAAGAAA | 57.5 |
| scaffold100_353 | JN902917 | F:TAAATATTTCAGCGGGGAGAACAA  R:CTGCTCATATTTCTGCTTCAACCC | 57.5 |
| scaffold79_3756 | JN902918 | F:GTGACACTTCCATGATGCCC  R:GATGACCAGGGACAGAGAGAC | 57.5 |
| scaffold794_23331 | JN902919 | F:TGGCACCACCATTGATACTGA  R:GCTTGTTGCCCTTCAGGT | 57.5 |
| scaffold870_2008 | JN902920 | F:CCTTTCCTGAAACCTAACTGGC  R:AGAAGAACCCAGAGCAGAACC | 57.5 |
| scaffold1722_950 | JN902921 | F:AGGCTGTTTAACTCCTCGGAC  R:GTTCATAGTGTGCAAGAGCATAGC | 57.5 |
| scaffold189_841 | JN902922 | F:ACCAAGGAAGAAGTGCGTAGAC  R:GACAAACAGACGTTGACCCTC | 57.5 |
| scaffold268_1368 | JN902923 | F:GGTGTCATGTTGAAGGCTGTC  R:TCCAAGTTCTTCTGAGCACTCC | 57.5 |
| scaffold693_446 | JN902924 | F:AAATGTCAACAGCACTGACACC  R:TGAAACTTGATGTAGTCACTCAGGC | 57.5 |
| scaffold560_163 | JN902925 | F:AGCCTGATAGAGGCTAATGATGC  R:ATGTGGACTTAAGGAGCAGAGTC | 57.5 |
| scaffold1029_2408 | JN902926 | F:TGCACGGTGTGATCTACAGTC  R:TGTGGTGCAATGTGTGGTTC | 57.5 |
| scaffold423_746 | JN902927 | F:GCCTGGTCAAATCAGATCCAC  R:GCTGATTCAGAGCTACGGACT | 57.5 |
| scaffold354_740 | JN902928 | F:GAATCTTCAACGGTTCCAGGC  R:AGGCAGAGTTCCTGAGTGAG | 57.5 |
| scaffold317_43 | JN902929 | F:TGGTCCAGTTAGTTTGAAGGAGG  R:GCATGAACAGACTCACAGAGG | 57.5 |
| scaffold317_91 | JN902930 | F:TACAGTGCATTAGGGGAACTCC  R:GCGTCTAACCAAAACCGTAATCG | 57.5 |
| scaffold1265_2428 | JN902931 | F:GATCTCCATTTTCCGGTCTGTG  R:TCTACAGTGACAACGTGTTATCGC | 57.5 |
| scaffold490_218 | JN902932 | F:CTGTTAGTATTCTGCACTAGGGTGTC  R:CAAGCTAGAAATGAAGTGTGACGTG | 57.5 |
| scaffold506_2264 | JN902933 | F:CTGTAAACTCCTGACATTGGCC  R:TAAAGGTCTCTCGACCCAAAGG | 57.5 |
| scaffold553_325 | JN902934 | F:TTTCTCCATGTGTGATGCTAAGGA  R:AACCCTCCCCAAATTAAATCAATG | 57.5 |
| scaffold781_1491 | JN902935 | F:AAACACGTCTCACCACCAGT  R:GTCATTTGTAGAGCATGTGAACACG | 57.5 |
| scaffold481_2972 | JN902936 | F:GAAGGAAGTGGAGAAGCCG  R:AGTGGAACAGGACGACTGAC | 57.5 |
| scaffold757_2801 | JN902937 | F:TGATAATCCTGGAGCGTCCAC  R:CACATGACTCCATCACGGC | 57.5 |
| scaffold631_2538 | JN902938 | F:GAAGACAGCGTGGTTACGTC  R:ACAGACAGGTGAGTGGGATC | 57.5 |
| scaffold304_3133 | JN902939 | F:TCTTTTTGCTGGTGTTCACAACC  R:GCAAACAGAGGACTCTACGC | 57.5 |
| scaffold464_2496 | JN902940 | F:GTGGTAATCACTGCTGCTCC  R:GGAATCAGGAATAATGATGGGGC | 57.5 |
| scaffold813_2625 | JN902941 | F:CTAGGTTTCAAGAGCGCCTG  R:CTTACTTGTGCAGCCAGACAATAC | 57.5 |
| scaffold255_2879 | JN902942 | F:CAGTGACTCCAAACGTTGACG  R:AGACAGACACCGTCGTCC | 57.5 |
| scaffold893_2732 | JN902943 | F:CAGTCTGTAATTTTTGGCTGTGGT  R:TGCACAATTCTGGATATGGGGT | 57.5 |
| scaffold170_2073 | JN902944 | F:GACTGTGTAAGCAGTGTGTCC  R:GTTATGGTGCCCCGTCTC | 57.5 |
| scaffold677_2938 | JN902945 | F:TCACAAGGGCGACTCGTT  R:TGTAGCAGTAGTGTGGAGGTC | 57.5 |
| scaffold76_1929 | JN902946 | F:AATGTACCAAAGCTGTCGCAC  R:GATTCTCTGTGGCAACCCC | 57.5 |
| scaffold676_2177 | JN902947 | F:AGTCTTTATTTTCCTCAGGGAGTGG  R:GAAGTATCGCATGGAGGTGC | 57.5 |
| scaffold831_2476 | JN902948 | F:GTAACGCTGGTGTGACCAC  R:GTGGAAGGATGTTAGGGGC | 57.5 |
| scaffold351_2697 | JN902949 | F:CCTCAGTCCTCCCCTCTCTAA  R:TACCAGGCTAGTGGTTTGTGG | 57.5 |
| scaffold282_2659 | JN902950 | F:CATTATGAAATTCTAGCCCACGGC  R:AGATAGACGAGCAGAGCCC | 57.5 |
| scaffold878_2753 | JN902951 | F:GAGAAAAGAGCACACAGGACG  R:GGTGTACAATAGCTCGCCAC | 57.5 |
| scaffold566_2385 | JN902952 | F:ATCACCAGGTTGGACACGT  R:TGAAGTCGATCAAGACGAAGTCG | 57.5 |
| scaffold589_3028 | JN902953 | F:GGTGAACTACAGACTGACGGT  R:TGTACGTGGAAATTCAAACGCC | 57.5 |
| scaffold636_2245 | JN902954 | F:GTCAGCTCCATAACCCAATCAG  R:CTGATCCTATCAGCGTGTGC | 57.5 |
| Scaffold000035 | JN902955 | F:AGAGGAGTGAAGTGAACGACCC  R:ATATTCCCATGAGCACATCCTGTT | 57.5 |
| scaffold1286_49924 | JN902956 | F:TCAGATAATGAGCCGGACAACG  R:TCCACAGGTGTGAGCCAATTAG | 57.5 |
| scaffold1286_49912 | JN902957 | F:TACAGTACCTGCAACTGTCTCC  R:TCCGACATGATTGATCTGGCT | 57.5 |
| scaffold1346_51929 | JN902958 | F:CGTTAGGCGACTCTTAGCGTT  R:ACAGAGGGGGGAGACATTTAAGA | 57.5 |
| scaffold2462_781 | JN902959 | F:GAATTACCACACCATGCCCCTA  R:GTTGGTACGGTGCAATGTGATTT | 57.5 |
| scaffold749_22217 | JN902960 | F:TCCACCACACCTTAAGGGAC  R:GGATGCTTGGATTCGATGCTTT | 57.5 |
| scaffold000127 | JN902961 | F:CAGAGACACGGGGGAGTAC  R:GGGGAGTTTCCATCGTCTTTCA | 57.5 |
| scaffold000216 | JN902962 | F:CAGATCTTGGTGTCTTGAAGAAGGT  R:CCCTGTACTGTCAATCGTGTCA | 57.5 |
| scaffold504_23626 | JN902963 | F:GTACGCAGCACTGGAACAG  R:GGCTGCAGAGGTATCACAGA | 57.5 |
| scaffold554_26291 | JN902964 | F:CAGGGAATCTGAGAAATGTCCCA  R:AAACCCAGAAAGGCAGCAG | 57.5 |
| scaffold593_28614 | JN902965 | R:GTGTCCTCCCTTGTTAAGTGGCT  F:ATTGCTTTCACAATTCAGCATCAG | 57.5 |
| scaffold078467 | JN902966 | R:AAACAGAGCAGCAGGAAAGGAGT  F:AGATCCTGATAATGTATGTGCCCG | 57.5 |
| scaffold16_902 | JN902967 | R:GGGAGTAGGTTTGAGGTCCA  F:CAGCACAGCTGAAGATGTCC | 57.5 |
| scaffold1823_60336 | JN902968 | R:CGAAGAGACTGGGTGGGAATAA  F:TCCTTCCAACAGCACGATTCTAC | 57.5 |
| scaffold000353 | JN902969 | R:CAACAGAGTACGCCTTAAGGGT  F:CGCAGCAAGAAAGTACCAGG | 57.5 |
| scaffold1970_62652 | JN902970 | R:GCGTTGAAGCTGACGACTCTT  F:CACGTGAAACAACTGGGAGTG | 57.5 |
| scaffold347_16764 | JN902971 | R:GCGTAATTACAGAGGAGCCAGT  F:GCATAATGAGAAAGAGGACGGGT | 57.5 |
| scaffold7810_71859 | JN902972 | R:CTTCACGTCAGTCCAACCATG  F:AGGAAGTCAGCTGCAAACGTAA | 57.5 |
| scaffold106_7285 | JN902973 | R:AGGCTCGGTTCAAAGACCA  F:GTGGACCAAGTTTGTTCACTTCATT | 57.5 |
| scaffold5643_71579 | JN902974 | R:AGACGACGTGAGTGAAAAGAAGC  F:GATGCCTTTAGTGTCTCCAGCA | 57.5 |
| scaffold000023 | JN902975 | R:TCAACACAGAGTGGTGCGA  R:TGCCTCTGATCTGCTCATGTC | 57.5 |
| scaffold079397 | JN902976 | F:ACAGACTGTCCTCCCTTCTCTCC  R:TGTTTGTGTGATTCTGCCTGAAGT | 57.5 |
| scaffold1093_44605 | JN902977 | F:CCAGGGAGATGATATTGACAACAGG  R:TTTGAACCTCCCTCTCTCTTCGT | 57.5 |
| scaffold203_9600 | JN902978 | F:GCCTTGAAAGCGTAGCCTCTA  R:ACTGCCTCCTTCACTGCA | 57.5 |
| scaffold2138_64139 | JN902979 | F:TGTGTGAATGGGTGGTCATC  R:AAACAGGAACTGGTGCATTC | 57.5 |
| scaffold7043_71761 | JN902980 | F:CTGTGTGACGAGGCAAAAGC  R:CGCCTTCATGCACAAAGGTT | 57.5 |
| scaffold7857_71871 | JN902981 | F:AGCCACGAGCAGATGAAAGAG  R:GGAAGGGAAGCAGAGGTGT | 57.5 |
| scaffold112_5684 | JN902982 | F:GTTCCACACCAATGACAGAGAC  R:GAGCTGTGTGCTAGCTGAAC | 57.5 |
| scaffold112_645 | JN902983 | F:TTATACGCCAACAACATGTGCC  R:TGTGAGACTCTGGAGGTGTC | 57.5 |
| scaffold2505_67588 | JN902984 | F:CAGTTTGAACCAACGGGAGAG  R:CAGATCCACTGTGCAGCTCA | 57.5 |
| scaffold2622_68432 | JN902985 | F:AGACTGAAGTGTGAACGGATTGG  R:TGCTGGTACTGCTCAGACC | 57.5 |
| scaffold28_1526 | JN902986 | F:TGATGTTCAACGCTGCTGTC  R:TGAGCCAATTAGGCCTGTTGA | 57.5 |
| scaffold3631_2291 | JN902987 | F:TGCACTGATGGATAAGCACAT  R:TGCACTGATGGATAAGCACAT | 57.5 |
| scaffold454_22122 | JN902988 | F:CGACACAAATTGCCTCACAC  R:TCTGCTGAAGAAAATGCAACC | 57.5 |
| scaffold607_29337 | JN902989 | F:GTGGAGACACAGAACAACAGGT  R:AGTGTGTCTGTGTTCAGCCTAAG | 57.5 |
| scaffold7769_71835 | JN902990 | F:TGAGTCGAGGCAAACCTGC  R:ACTGCCGTCGTCTTCACTTC | 57.5 |
| scaffold865_37021 | JN902991 | F:AAGATCAAACAGTGCCTCGGA  R:AAAGGATTAATTTCTTGAACGGCATG | 57.5 |
| Scaffold080405 | JN902992 | F:CCAGCACAGAGACTTGGC  R:GAGGTTCCACCCTCTTGTTCA | 57.5 |
| scaffold1055_42679 | JN902993 | F:GGAGTCACATCCTGATCCAGTG  R:TGTTGGTGGTCTTCATGTTGGAA | 57.5 |
| scaffold1748_58947 | JN902994 | F:GCTCCAGGAGTGGAACCATA  R:GACCCGTGCTCGTGATAAAAAC | 57.5 |
| scaffold857_39088 | JN902995 | F:AGGAACGTTCGACAAAGAGAGG  R:ATGGTGATGAGGTTTGGTTCACA | 57.5 |
| scaffold000294 | JN902996 | F:GTCATTCATGTTGTTGGCGTGA  R:GCTTACTGCACTGACGGATTG | 57.5 |
| scaffold1412_53141 | JN902997 | F:CGCCCACTGTTTGAGAAGTACA  R:AAGCTCCAACACATTAGCCACA | 57.5 |
| scaffold1425_53260 | JN902998 | F:GGGGGCAGATTCTTGAATGACT  R:GGGCCAGATCTACACCAACT | 57.5 |
| scaffold1771_59501 | JN902999 | F:ACGTCCAATCAGAGGACACAG  R:CTGAGGTGTCGGACAGAAGAC | 57.5 |
| scaffold1965_63173 | JN903000 | F:CAAGGTCACGCTCATAAACAGC  R:ACCCATATGAAAATGCCCTGCA | 57.5 |
| scaffold936_39673 | JN903001 | F:ATTTCACAGTCAGGACGACGAG  R:TCAGCAAGTGAAGTTTTCAAAGGGA | 57.5 |
| scaffold080131 | JN903002 | F:GACAGAGTGTCGGGTTCACT  R:GACAATGTCATGCTGTCTTGCTC | 57.5 |
| scaffold1784_59258 | JN903003 | F:ATCAATCAGACTGCAGCAGAGTAAAG  R:CACACAAACACCATGCAGTCAT | 57.5 |
| scaffold2231_65427 | JN903004 | F:GGTTTTAGGCCCATTGTTTCTGAG  R:CCATGATTCCTCGGCTTCTTCTT | 57.5 |
| scaffold2900_69270 | JN903005 | F:GACACGTACTCACACACTCCA  R:CTTTCACCTCCTCCCCTTTTCT | 57.5 |
| scaffold5_4 | JN903006 | F:CACAGTCTTTGGAGGCCAATTAC  R:CTATCTGTCTATCCAGTCCACCCT | 57.5 |
| scaffold078393 | JN903007 | F:AGCATGACACAATGAGCAGGA  R:GCTCTGTGTCCAAACGGTG | 57.5 |
| scaffold374_18282 | JN903008 | F:TTCCATAGTTGGTTGTTGTCACGT  R:GGGAAATGGGAGTACGAGGT | 57.5 |
| Scaffold000005 | JN903009 | F:GACATGGACAACCAACTTTGCA  R:CTGCAACCAGAGGAGAAAGACT | 57.5 |
| scaffold1746_59096 | JN903010 | F:GATGGCATGGTGTGAAAGACAG  R:GATTTTTACCTTCTGCCCCTTGG | 57.5 |
| scaffold2391_66684 | JN903011 | F:TGAGGGAGGGGGTAAGTTTG  R:GTGACACCCACCAAGATCTCATT | 57.5 |
| scaffold511_24239 | JN903012 | F:GCTGCAGCCTCTGTCTTTAC  R:CTGCAACTTCTCATCACTACTGTCAC | 57.5 |
| scaffold1424_53263 | JN903013 | F:TCAGATTAGCGCTAGGGTAAGGA  R:TTTTTGCGAGAGGGAACACTG | 57.5 |
| Scaffold000193 | JN903014 | F:CCAGTCTTTGGGAAACAGTCG  R:GGTGGCTGCTCTACTTGC | 57.5 |
| scaffold1002_41697 | JN903015 | F:GAATAGGAGAAGCAGGGTGGT  R:TGGTACAGAGTTAATGGTTTGATATTCA | 57.5 |
| scaffold2046_63282 | JN903016 | F:TCCTTCAGGTTCAGGTAGCAC  R:GACGAGCGAGTCAAATGTCTGT | 57.5 |
| scaffold5402_71541 | JN903017 | F:ATTGAAAGAGTCCGGCAGATGT  R:CGTTCCTATTCCTGCCTTCAGT | 57.5 |
| scaffold57_2019 | JN903018 | F:ACATGTGGAGGGAACCAAGAG  R:TCTGCCACTTCATCAAAACTTGC | 57.5 |
| scaffold448_22034 | JN903019 | F:TCATGGAGCGAGTCAACAAG  R:CCAGAGGTTCACTCACGAT | 57.5 |
| scaffold172_9047 | JN903020 | F:TACCACAAAAGCTTTGCAACCC  R:GTACACTAGTGCTGGGCAACA | 57.5 |
| scaffold2017_63115 | JN903021 | F:GAACCGTACGACATCACAGGAG  R:CGCTGCGTGAATAATGATGACC | 57.5 |
| scaffold2115_64237 | JN903022 | F:CAAACTTCTGCCGTCCACAG  R:GGTGCCAACAAAGAGAAATGC | 57.5 |
| scaffold2313_65865 | JN903023 | F:ACGACATTTCAGGGCAGATTTGA  R:AGAAGAACAAAGGCCGGAAGTT | 57.5 |
| scaffold547_25916 | JN903024 | F:CACTGGGGGAGTAAGCAAGAA  R:AGCTGTGCTAACAACCAGGATT | 57.5 |
| scaffold6262_71664 | JN903025 | F:GGCAGTGGATTTGCTATTTCCTG  R:GTCTTCACGGCTCCTGTTTTC | 57.5 |
| scaffold1132_45520 | JN903026 | F:CCTTTAGTCCCCCTAGTGTCC  R:TCTGTCCAATCACGTGTCTCC | 57.5 |
| scaffold30_2675 | JN903027 | F:CGACTGTCCAGTGAAATTGAGACA  R:CCAGACGAGTTACGTTCCACA | 57.5 |
| scaffold000174 | JN903028 | F:TGCAGTGTTGAAAGGGAGGA  R:ACGAAGAACATATTGGTACCAGGA | 57.5 |
| scaffold654_30814 | JN903029 | F:TTCCAGCGTTATCGTCGATTTCT  R:ATGTGTGGGAAAGATGGCAAG | 57.5 |
| scaffold984_40533 | JN903030 | F:CTGCTGGTAGGACCCTGAAA  R:AGAATTCAGCTGCTGTTCGCT | 57.5 |
| scaffold391_19156 | JN903031 | F:TCGTGAACAACAGCTTGAGGA  R:TCAACATGAGTTGACCTTCTCCA | 57.5 |
| scaffold167_8468 | JN903032 | F:TTCGTGCTTGTGGTGTGATATGT  R:AGCAACAGTTCAGTCACGGT | 57.5 |
| scaffold42_2392 | JN903033 | F:GAAGCAGAGAGCAGGGGAAATA  R:ATTGTCGCGTGTCGCAC | 57.5 |
| scaffold000030_774 | JN903034 | F:CCTTTCAGAGACAGGCCAAC  R:GGGACGTTTACTGGCTCAC | 57.5 |
| Scaffold078488 | JN903035 | F:CTCACAGTGGACATTCAACTACGA  R:TGCTCACGCAATGCTGATG | 57.5 |
| scaffold198_7183 | JN903036 | F:ACTTCCTGTGTGGATATTAGGGC  R:CCTGCTTAACCAAGCACCTC | 57.5 |
| scaffold410_20474 | JN903037 | F:CTCTCTCCATCTGTTCCTGTGG  R:TGGAAGGACTTCATGACTGTGG | 57.5 |
| ST6 | JQ272743 | F;ATGTACCCAAACACTTCAAAGC  R:CGCAAATGTCTGTACCTCCTTA | 55 |
| ST10 | JQ272744 | F:CGGACTCTGGTTTAGAGTTT  R:CTTCCTCCAGTCCACTAAAT | 53 |
| ST15 | JQ272745 | F:GAAGAAGGGTGAGAAACAGAAAGT  R:CCTCGTGTTTGATTTGATTGTC | 55 |
| ST19 | JQ272746 | F:CGTGTATTCAGGATTATGGCGTAG  R:CAACCCCTGGTGTTAATGGC | 57.5 |
| ST26 | JQ272747 | F:CAACAACTACACCGTCAACCGT  R:GACGCTCAATGAAAATGCTCTG | 58 |
| ST30 | JQ272748 | F:CAGTCCGACAGGTGATTTTA  R:CACCTACGACCTTGACATTG | 54.1 |
| ST31 | JQ272749 | F:ATTTTGACGGACACGAGGAA  R:TCGTCACGCGAACTTTAAGA | 55 |
| ST40 | JQ272750 | F:CGCTTCGTTTGTTCACTTCA  R:CAGTAGAGGCTAATGGTGGCA | 57 |
| ST43 | JQ272751 | F:GATTCCTTTTTTTCCCCCAA  R:ATCAGGCTAACCATCGGAAA | 55 |
| ST50 | JQ272752 | F:CAGCGTTCTGATGATGTGGGA  R:TCGGGATGAGAAGGACTGGTG | 59 |
| ST59 | JQ272753 | F:GTTACCCACGGCAACACTTA  R:AACTGCTGCTGTTTGGATTTA | 56 |
| ST87 | JQ272754 | F:GTTTAAGGGCTTCCCAGTG  R:TTGTTCAGATTAATAGTGTGACAGA | 55 |
| ST109 | JQ272755 | F:ACTCGCATGGCATCAATCAC  R:CATTCAGCTCCCTGGACAAA | 57 |
| ST130 | JQ272756 | F:AGGGATGTTTGTTAGGATGAA  R;GGCAGCATAGAAAAGGAAAG | 53 |
| ST151 | JQ272757 | F:CTCAGCCACACATCTCAGCA  R:CCTAATTGGCATTGCTTGTG | 58 |
| ST152 | JQ272758 | F:AGTCTGTCTGCGACGCTGTG  R:AGGGCGAAATGTAACTTATCCA | 60 |
| ST153 | JQ272759 | F:CTAAATGTCCTTTGCCTGTC  R:CCAAGTAAAGAAAACCTTCTAG | 52 |
| ST154 | JQ272760 | F:TGTAGGTTTTTGTGGACATCTT  R:CCTGGGCTAAAACCAAACA | 54 |
| ST155 | JQ272761 | F:GGTTTCCCGTTACCTTCTGT  R:CCATCCAGTCTCCCTAATCTA | 55 |
| ST157 | JQ272762 | F:GTCAGGTTGTAATCATAGAGCACT  R:ATCTTGCACCGGCGTTT | 56 |
| ST160 | JQ272763 | F:TCCGTTTTCCTTTCAATGCT  R:CTCATAAGCGTTTGGGGAAT | 54 |
| ST166 | JQ272764 | F:CGTGTCTGTTATGGCACCTT  R:TCCTGGAGCAGATCACTAAGTC | 56 |
| ST169 | JQ272765 | F:GACAGCAAGGTAAAAGAAGA  R:TTCACAGCAGTTTTGGTAGT | 53 |
| ST170 | JQ272766 | F:CGAACGATGAGAATTTGGAATA  R:GGACTTCACAGCAGTTTTGG | 55 |
| ST173 | JQ272767 | F:CAGCACCGCATTCTGAACCT  R:GTGGATTACGGGATGAGCCT | 59 |
| ST175 | JQ272768 | F:CGGGTCTGAACCAGTCCAAAC  R:TAGGTGGATGTGGGCGAAGG | 60 |
| ST176 | JQ272769 | F:AAGCAGTCTTATCAGTTCCC  R:TCCTCTTTCTTCCATTTGAC | 53 |
| ST177 | JQ272770 | F:GTCAACAGTGCGACATCTGAA  R:ACATGCCGGAATAAAGTGGA | 56 |
| ST181 | JQ272771 | F:CGCATCAGGACGAAACATTG  R:CTGCTTGTTTGCGGCTGTC | 58.5 |
| ST184 | JQ272772 | F:CGATTCCGTGACCATTCAGA  R:CGCTTTAATGGATCAGTTGGATA | 55 |
| ST186 | JQ272773 | F:CGACTGACAATGACTGACTTCTG  R:TATCTTGCACCGGCGTTT | 56 |
| ST192 | JQ272774 | F:TAATCAACCTCCAACTCCCT  R:AGAAAGAAAACTGTGCGATG | 53.5 |
| ST193 | JQ272775 | F:CGGACTGGCAGGTAGTAGAG  R:GCCACATTTCCTTCCTTATC | 57 |
| ST195 | JQ272776 | F:GTGCTCACTGGCGGAATAAT  R:TGCTTCTTTCCTTTGCTTTGT | 56 |
| ST196 | JQ272777 | F:ACAGCACCATGGGGTATTGA  R:GCATTTGACTTCAACAGTGTCAT | 57 |
| ST204 | JQ272778 | F:GCGTTTTCTGGCTTGAGTGA  R:ACCAGCAGACAAACCAGGAGT | 59 |
| ST208 | JQ272779 | F:TGAGTTTCATGCGCAATTTC  R:CACAGGTTCCTAATGGCAGAG | 56 |
| ST209 | JQ272780 | F:CAATGAGCAACAATAACACC  R:TCTCCCTTTGTCTCAATCTC | 52 |
| ST210 | JQ272781 | F:GCTCTGCCATTAGGAACCTG  R:TACAGGCGGAGTTGTTTTGA | 57 |
| ST211 | JQ272782 | F:GGTGAAAACACGCTTAGGAA  R:ACTGTGAGAAAAGCAGAGGAA | 54.5 |
| ST213 | JQ272783 | F:AAGAGCGGCTGGCGGTAGAG  R:TGCGACGGCAGGTTAGAAGAG | 62 |
| ST214 | JQ272784 | F:AAGTCTGTGAGAAGGTGGAG  R:TAAAGACATAACGAGGCACT | 54.5 |
| ST215 | JQ272785 | F:ACACTTTTGTAATCGGCGTCC  R:CAATCTACAAGAGTGCGGTGC | 57 |
| ST219 | JQ272786 | F:CTCCTCCCGCTCCCTCAGTA  R:CGCAAATGATCTTGCCTCAAT | 60 |
| ST222 | JQ272787 | F:TAAGGCATTGTCAAAGAGTGT  R:TGATGACGATGATAGGGATG | 53 |
| ST227 | JQ272788 | F:TGTTTATCACTAACTTGACGCTGT  R:CAATGCTGCCTGATGCTAAT | 55 |
| ST228 | JQ272789 | F:TTCCAGGCGTACTGCTACAT  R:CGTTTCATTCCGTTCACTTT | 56 |
| ST232 | JQ272790 | F:ACGCTGTCAGAGGTTGCTTC  R:TGTCCCTGAATTTGGTTTGG | 58 |
| ST233 | JQ272791 | F:ACCATTCACAGTCTTCAATCGTC  R:GTGACAGCGAGTGGATCGTG | 59 |
| A73-56 | EU159320 | F: GCGGCTTCCGTCTCAGGTT  R:CCTTCAAAGCGCAGCGTCA | 63 |
| B121-20 | EU159331 | F: CTATTTACAGGGCTACAGTC  R: GTCCACAGGAAGCACCAT | 60 |
| cs006 | FJ476269 | F:AGTAAACAGACCTGACCAATCAG  R: CGGACTACCAATGCTAATGTG | 64 |
| cs007 | FJ476270 | F: TGCCTGCTGTTCATGGATAG  R: TGGGATTGCCACAAAGACAG | 67.5 |
| cs10 | FJ476271 | F: TCCTGTCATTTCATTCCTTTG  R: ACCTACTGATGAAGAAAGGTTG | 60.5 |
| cs26 | FJ476275 | F: TCAATGGGTGGAGTTAACGTC  R: AGCCAAGTGCAAGAGCAATC | 67.5 |
| cs54 | FJ476279 | F: CCTGTCAACTTGCATCAGAAG  R: AGGGATGTGCATGAATGTTG | 65.5 |
| cs61 | FJ476280 | F: ATGGCATTCGACTGTTCTCAG  R: GATAGGGGCTGCTGGTACTTAC | 66.5 |
| cs67 | FJ476281 | F: GGCATCGTTTTTTCAACATC  R: ATTGAGCCACAAAGCTACTTG | 63.5 |
| cs77 | FJ476283 | F: AATGATGCTGCCGCATCTTC  R: AATGTCATTGCATTACGATGCAG | 66.5 |
| cs101 | FJ476288 | F: TCGTGATAGAAAAACACAGGTC  R: CCAAAGAGAGAACAAATCAAAG | 60.5 |
| cse009 | GH235041 | F: CAGGACAGGCATGAGACTGA  R: CCTCCTCTGCTGTTCTCACC | 54 |
| cse22 | GH232828 | F: GGGAGTCAACAACCAAACCA  R:CACTCGCAAAGACTGAGGTG | 55 |
| cse36 | GH234927 | F: TTTCACCCAAGATGGAGGAG  R: CTCCCTTCTCGTCTCCACAC | 50 |
| cse42 | GH229582 | F: ACTGCGTCCAAACTGAGGAG  R: CATTCCCCACTCACTGTCCT | 54 |
| cse45 | GH232494 | F: GGGGAGAGAGTCAAAGACAAAG  R: TGTCAACGCAGAGCTGAAAG | 53 |
| cse54 | GH231568 | F: AGAGCTGCTGGCTCTGAATC  R: GACAGCCTCAACCTCAGGAC | 55 |
| cse55 | GH233577 | F: TTGTTGCTGTTCTGGTTTCG  R: CACACTGCTCCGCTGAGATA | 52 |
| cse56 | GH231141 | F: TCGATCAGATGGAGGTGAAA  R:AGCTGTTGAGCACATTTATTTATTT | 52 |
| cse61 | GH230059 | F: GTCCAACAACAACAGCAGCA  R: CTGGAAGTTGAAGCCCTGTC | 54 |
| cse66 | GH231699 | F: TACTGAACACCGTCCACCAA  R: CCAAGTCAACTCGGTTCTGA | 55 |
| cse68 | GH237871 | F: CCCTTCCATCTTTCCTTCCT  R: AGCAGCAAACCTTCACAACC | 54 |
| cse71 | GH236888 | F: TTCTGTGGGAGGAGAGGAGA  R: ACACACCTGCACACACCAGT | 54 |
| cse72 | GH238433 | F: TGCGAGGGGTAACAGGATTA  R: CAGCACGAGAGACGAAACTG | 53 |
| cse73 | GH229236 | F: CTAGCGCAGGACTCAGGAAT  R: GCCATCCTGCTCCAATAGTC | 55 |
| cse74 | GH229354 | F: GCCAGCTGGAAATAATCTGG  R: CCCTGGCAGGTAATAACGAC | 52 |
| cse83 | GH230001 | F: CACTGTGCTCCACTCCAAGA  R: AGACCTCCGTAGGCTGTGAA | 55 |
| cse84 | GH230045 | F: GTCGCTCTCAGCAGACACAC  R: GATCCCTGATGCTGACGAGT | 55 |
| cse85 | GH230045 | F: AGGTGGAGGAGGAAAATACCA  R: GTCACCGAGACGAACAAACA | 55 |
| cse86 | GH230045 | F: AGTGGACGAGTTCCATGACC  R: TCCATGTCTGTCCCCTCTTC | 51 |
| cse92 | GH230045 | F: AGTGGACGAGTTCCATGACC  R: TCCATGTCTGTCCCCTCTTC | 53 |
| cse98 | GH231344 | F: GCCAGCTGAGGATCAGAGAG  R: GGAAGGAAGGAAGAAAGAGCA | 51 |
| cse105 | GH231811 | F: CCCGCATTTGTAAGAAGGAA  R: TATCACCGCTCAATCACTGC | 51 |
| cse112 | GH232114 | F: CCATCCGAGACCTTCAGTGT  R: ACCTGCTCCTCCTCTGTGAG | 54 |
| cse119 | GH232548 | F: TGCTGCTGAGGAGAGTTCTG  R: CCCTGTTTCAGCTCTTCCAG | 55 |
| cse120 | GH232637 | F: GGTTCAGACGAAACGGTGAT  R: CCGCGTTTAATCCCCTTACT | 52 |
| cse122 | GH232637 | F: TGACCCAGGTCTGTACTGTTTG  R: ACTGGACGCTTCATTCTCGT | 54 |
| cse123 | GH232882 | F: GACAATTTTCCACCCCACTG  R: TCACAGTTGTTCGCTGTTCC | 51 |
| cse134 | GH232882 | F: CACAGAGCTCCACCATCTCA  R: TACGGAGGCTTGGTAGTGCT | 53 |
| cse135 | GH233446 | F: TGCGTAAATTTGGTTTTGGA  R: AAGAGAAAAGCCAGGCACAA | 52 |
| cse138 | GH233643 | F: AGCTGTGAATTTGCATGTGG  R: TTTTGGTGTGGCAGAGTGAC | 52 |
| cse148 | GH234255 | F: TGTGGGGAATGAAGTTTGAA  R: TAAGAGTGTGCCGCTTCCTT | 50 |
| cse151 | GH234379 | F: ACAAAGCTGCGTTCCTATCG  R: GGATCCTGCCTTTGGTTTCT | 53 |
| cse153 | GH234563 | F: GTTCCCTGTGATCCAGCACT  R: GACTGGATGTGGTGGATTGTT | 55 |
| cse155 | GH234673 | F: CCATTCACGCCTTTAACACA  R: CCGGTTTACTTCCGTGTTTG | 52 |
| cse160 | GH234992 | F: CGAAGGCCAACACTTTGATT  R: TCACAGATCGTGCAGTGACA | 52 |
| cse164 | GH235022 | F: GAATCATGCGAACACCTTCA  R: AGAAGATGGCACTGGGTCAC | 51 |
| cse167 | GH235148 | F: CACATTTCATGGCAGCAGAT  R: CCAGCTTCGAAGACGATACC | 51 |
| cse176 | GH235365 | F: ATGGGCTGGTGCAGATAGAG  R: GGAGCTGCTGATGATCCTTC | 55 |
| cse179 | GH235529 | F: ATCGAGGAGGATCTGTGTGG  R:GCGTGAGATGCAAGAAAACA | 53 |
| cse190 | GH235859 | F: TTCACTTCCACCTCCTGACC  R: AACCCCTGAACCTAAGATGG | 52 |
| cse194 | GH235987 | F: AAAGCTCTCTCCCGACCACT  R: TTTCACAGTTCGGACCCTTT | 51 |
| cse195 | GH236050 | F: ATGGTCGGATTATCGCAGAG  R: TACCCTGGATGCCTTCAGTT | 51.5 |
| cse196 | GH236076 | F: GGGAGGAGAGGAGGACAAAC  R: CATTTCCTGGACTCACACCA | 53 |
| cse206 | GH236748 | F: CCTCAGAGTGAGGCCTTTTG  R: CCTGCAAAATGGGAAGGTAA | 51 |
| cse208 | GH236972 | F: GCACACAAAAATGGAACGAC  R: GCCTATGAGGTTAATGCCACA | 50 |
| cse221 | GH237405 | F: GGGCATTTAAAGAAGCGTTG  R: GAACGTTTGGTTCAACAGCA | 51 |
| cse224 | GH237561 | F: TTTGTTCATGTCGTGCCAGT  R: GGCGGTTACGAAAGTACCAG | 52 |
| cse225 | GH237637 | TGCTGAGGAACACTGCGTTA  TTTTCCTGCAACAACATCCA | 51 |
| cse228 | GH237834 | F: GGAGCAAGAACACTGGGCTA  R: TTTTTCTGGTCCAGCATTGA | 51 |
| cse236 | GH238140 | F: TACACCCTCACAGCGAAATG  R: GTGAGGCTCGATCACTCCAG | 54 |
| cse243 | GH238303 | F: TCCCTGAACCTGAAGACGAT  R: AGTGTCGTCTGGACATGTGG | 52 |
| cse248 | GH238518 | F: CGCCTGTTTCTTACGGAGAG  R: AGATTGACGCTTCTGCGTTT | 53 |
| cse249 | GH238528 | F: GCAGCAGCGTAGGTCTCTTT  R: CCTTGAACCTGAGACTACTGGAA | 55 |
| cse251 | GH238695 | F: TGCAGCGCACATTACCTAAC  R: GGCACGCTGAAGCTTTACA | 55.5 |
| cse252 | GH238801 | F: TACGTTCAAGTTCGCATCCA  R: TCGAGTAAAAGCCACCGAAG | 51 |
| cse257 | GH238923 | F: CCTGGGTGGAATTGGACATA  R: GCTGAGCGTCCAGAAGAATC | 50 |
| cse266 | GH239197 | F: CCACAGACCACTCTGTGCAT  R: CCCCCTCCCCCATGATTA | 51 |
| csou05 | EU159308 | F: TTCTCGAAGGGAACTCCAGA  R: GCTGGGCCACAGTAATCCTA | 58 |
| csou06 | EU159309 | F: CATGTGAGTTGGAGGTGTGA  R: GGCGTGGATGAACTGTAAAA | 58 |
| csou07 | EU159310 | F: CCCAACATTAGGCAATTCAC  R: ACTCTTTCAAGTGGCTGCAT | 58 |
| csou12 | EU159316 | F: CGTTGCCATCACACAATTTC  R: CAACGTGCATCGTGTCTCTT | 60 |
| csou15 | EU159318 | F: CCCTGTGGACTGATATAAACA  R: AATCCAAGTGGCATAAACAC | 55 |
| csou16 | EU159319 | F: TGTGAAGTCATGTGGTTCTG  R: TGAGTTGGTTGTATGTCCTG | 55 |
| csou19 | EU159323 | F: CCAAGGCTGTGTCGAAAAGT  R: TCATGGTGCTGAAGAGTTGC | 58 |
| csou21 | EU159324 | F: TAGTAAGTCCCTGCGGTTG  R: CATCCCACAGGAATCAAAAG | 58 |
| csou22 | EU159325 | F: GGCTGCCTACAAACCTCAAC  R: GAGGACTCAGATGCACACGA | 58 |
| csou24 | EU159327 | F:CGGTCGTCATGCTAATGTGT  R:AGATCTGGCGGCTAATTGTG | 60 |
| csou27 | EU159332 | F: ACAAACCCCAGAAGAAGTG  R:AATCAGGAAGCTACGTTCAG | 55 |
| csou30 | EU159336 | F: TAATCCCTGGTGCATCTTCA  R:TGTTGCCACTTAGCCAACTC | 59 |
| csou31 | EU159337 | F: CCGTCAGTGTTTTTGGTGAA  R: CAGTTCCCTGTCAAGGTTCAA | 60 |
| csou32 | EU159339 | F: TGCTGCTGCTGCTCATTAGT  R:CGGTCGGGTTTTAGATCAGA | 60 |
| csou33 | EU159340 | F: TTGGGCAAAAGTATAACAAC  R:TGCTAACATGTGATTTTGTG | 53 |
| csou34 | EU159340 | F: GCGGCTGTAGATTCAGATTT  R: CTTCCTGCTCCACAGTTTCT | 57 |
| csou36 | EU159342 | F: AAATCACAGACGTCCATTTC  R: GAAGACGTTCATTCTTCAGC | 55 |
| csou37 | EU159342 | F: GTTTCCTTCCTTTCATTCCT  R:GAAATGGACGTCTGTGATTT | 55 |
| csou38 | EU159343 | F: CGGTTTAAATCCCCGTCATA  R:CCCTCTTTGGACCATGTGTT | 60 |
| csou39 | EU159344 | F: CACAAAATTGCTGCTTGCAT  R:AACACTCCAACCCCAGGTAA | 60 |
| csou41 | EU159346 | F: AGTGAACCAAACAGAGGCTCA  R:CCCAAACACAAACCCGATAG | 60 |
| csou42 | EU159347 | F:CTGCCGTGATCTTCACAAAA  R:TCCACTGGGACAAGAAAACA | 58 |
| csou43 | EU159347 | F:GGAACTGCATGTCATTAACC  R:CGTCCTAGTGTGTCTAATGGT | 55 |
| csou45 | EU159348 | F:CAGTGGCTGTGGTTGTCAGT  R:GGAGTGAGTGGGCTGTGAGT | 59 |
| csou46 | EU159349 | F:CTGTGACCCTCCTCCTGCT  R:CTCCATGCTGCCAGTGTTT | 60 |
| csou50 | EU159353 | F: AGGGGAGGGGGAGATAAAAT  R: TCAAATCCAATCGTGACCAG | 60 |
| csou51 | EU159353 | F: CGGTGACTAAGCAGTGTGAGA  R: TGAGTGTTGCTCATCCTTCC | 60 |
| csou52 | EU159354 | F:TGGAGCAGCACACTGATTTC  R:CGTGGAGGGAACAAAGAGA | 60 |
| csou54 | EU159356 | F: GATCATGGCAGATGCACAGT  R: ACAACGCCTTCGTAATTGCT | 60 |
| csou56 | EU159358 | F:GGATAGAACATCATGCCCAGA  R:CAGTTGGTTGGGTGACTGTG | 60 |
| cyse03 | EF363801 | F: TCATGCCTAAGCAATGTTCTTG  R: CCAGCACAGCATCTCCTACA | 60 |
| cyse05 | EF363803 | F:TGATGATGCAGTCTGTGCTG  R: TAACCGTCCATCCATCTCCT | 60 |
| cyse07 | EF363805 | F: CCACTGGGATGGATGTCTCT  R:GTGCTGGTCTTGCTGTCAGA | 60 |
| cyse08 | EF363806 | F:CCACAATGGCACTTTCTGAC  R:GGAAGAGGAACTGCCAATCA | 60 |
| cyse13 | EF363811 | F: CCCTAATCCAATCGGGAAAT  R: CCTCTTTCACTCCCATGTCG | 60 |
| cyse20 | EF363818 | F: CGTCAGGCAACCTTCAAACT  R: GGAGTACTTTGCCTGCAATTAG | 60 |
| cyse37 | EF363835 | F:TTTTTGTGCGTACTGGTGGA  R:TCTTATGAGAATCCCTTATC | 60 |
| cyse42 | EF363840 | F: CTAATGACGCAGCACAAGGA  R: ATCACACATGCACCATCCAC | 60 |
| cyse43 | EF363841 | F:GGATGGTGCATGTGTGATGT  R:CTGGGAGAAGAGAGCACTGG | 60 |
| cyse47 | EF363845 | F: CGGACGGAGAGTAAAAACAGA  R:TGGTATGAACACTGAACCTGAGA | 60 |
| cyse57 | EF363855 | F: AAAGGGCAGATCATCACAGC  R: ATTGGCAGAGGTAAGCCACA | 60 |
| cyse58 | EU906934 | F:GGTCACTGTAAGCACTGGAT  R: AGTGTGTGTCCAAGGAGTTC | 55 |
| cyse60 | EU906935 | F:AACAGGTCTGGGTTGAAGAT  R: AGCACTCTGCTCACTGATGT | 56 |
| cyse61 | EU906936 | F:TCCTCAGCTCTTCACAGATT  R: TCACGGTCTACTTCTCGACT | 55 |
| cyse66 | EU906940 | F: TTGGTGCTGTGTTCCAGTGT  R: GAGGATGTGAGGGGTTTTGA | 60 |
| cyse69 | EU906943 | F: GACAACAGTCATTCCCTTCG  R: GCACAGTTTGGGGAGAGAAA | 60 |
| cyse71 | EU906945 | F: TGTTCTCACCCAAATTGCAG  R:TTTGCACAGATTGCTTTTCG | 60 |
| cyse72 | EU906946 | F:TGGTTGGTGGTTGCTCTACA  R:CTGTCCCTCCTTCTCTGCTG | 60 |
| cyse79 | EU906953 | F:ATGTCCCCACCACAATAGTA  R:ATGAAAGACACAGGAGGATG | 55 |
| cyse80 | EU906954 | F:GGCAAAACACAAGGTAAGAC  R:TCCAGAAGTTTCAGACCATC | 55 |
| cyse86 | EU906960 | F: AAGGAATCTCACTTCAAGCA  R: CAAAAACCCACTGAAGAATC | 55 |
| cyse87 | EU906961 | F:TTATTTCAGGGTGTCAGGAC  R:GGCTGTCTATGAGGAAACAG | 55 |
| cyse88 | EU906961 | F:TCAGACCTTCAGGGAACACC  R:CAACCCACCCATAACTTGCT | 60 |
| cyse89 | EU906962 | F: TTGGTGTCCACGTCAGTGTT  R: AGCCCCTCTTTACTGCCTGT | 60 |
| cyse90 | EU906963 | F:CTGATGTGTCAGAACCTGGA  R: TGTTTTGTCTGTTTCGATGC | 57 |
| cyse95 | EU906968 | F: TGCTGCCTCATATACAAACA  R: TCTCGTCCCTAAAACAAAGA | 55 |
| cyse98 | EU906971 | F: GTTATTCGGTTGGGAGCTAA  R: GTGTGGAATCTGCCGTTTAT | 57 |
| cyse102 | EU906974 | F: TGCTGGGCTAATTTGACTGA  R: TCTCATATGCCTGCATCCAC | 60 |
| cyse104 | EU906976 | F: GCACGCATTAGCATAGCGTA  R: AGACTGGGTGTCAGGGACAG | 60 |
| cyse107 | EU906979 | F: CCACACAAAAATCTTTCGAC\  R: TCCATGATCCACCTAATGAT | 55 |
| cyse111 | EU906983 | F: GACCCCTGCTGTTATTCCAA  R: GGGGTGTGGAGTTTCCTGTA | 58 |
| cyse112 | EU906984 | F: TTTGAAGTGTGCGTTTACCT  R: CAGCAGATTCTCATCTCTGC | 56 |
| cyse119 | EU906991 | F: TGGCAAAGAAGCCTTTATGA  R: CGCTGGATGGGTTCTATATG | 58 |
| cyse140 | EU907011 | F: ACTGTACGGAACTCGCTGCT  R:GTGACCAGGAAGAGGTTGGA | 60 |
| cyse143 | EU907014 | F: TTGCACAAGGGATGTGACTC  R: TGGTGAGCCCTTCTTGATTT | 60 |
| cyse147 | EU907018 | F:ACCCAAAGCAAACACAAAGG  R:GATCGATGGGTGGATAGCAC | 60 |
| cyse148 | EU907019 | F:GCGACCGCTCAAATCTAGTC  R:CAAACTGCCTGTCAGCTCCT | 60 |
| cyse149 | EU907020 | F:CGTCTCTGCGCCTCTTAGTT  R:ACAGAGGGGCAGGTCACTTA | 60 |
| cyse150 | EU907021 | F: AAATTTGAGGCAATACGAGT  R: TCGAAAGAGGAGAACAACAC | 55 |
| cyse155 | EU907026 | F:ACAACAGATGCACACGCATT  R:ATCGCAAACACAAAGTGACG | 60 |
| cyse160 | EU907031 | F: GGACCTGCTGCTGTTATGTC  R: CTGCACAGGAGTGAACTGTG | 58 |
| cyse161 | EU907032 | F:GCTTTGACCTTTACTTGTCG  R:CTAATGCCTCAAGAAGATGG | 55 |
| cyse166 | EU907037 | F: CGTACACACGCTGAAATTG  R: GAGAGGGAGAAAAAGCAAAC | 56 |
| cyse174 | EU907044 | F:TGTTGTCCGACTCTGTGCTC  R:TGTCTGTCTGGCGAGATGAA | 60 |
| cyse183 | EU907053 | F:GAAGCTGTTGAGCTGGTGTT  R:CTGGGGAATCATTTTCAGTG | 58 |
| cyse184 | EU907054 | F: AGCGAAATGGAATGTCTCTA  R: CTGACAGCTCTGATTTCCAT | 55 |
| cyse190 | EU907060 | F: GGCCTAATGTGCTGTTTTAC  R: AGAGCTCCTGATTTTGTGAA | 55 |
| cyse193 | EU907062 | F: GACGGACATGTTCAACCAGA  R: CCTGAAGGGGTGTTACCTCA | 60 |
| cyse195 | EU907064 | F:TGAACTGCAAGACTGCTCCA  R:CATCAGTTGGTGGCCTGTAA | 60 |
| cyse199 | EU907066 | F: CTGCCAGGATGAAGTTAGAG  R: AAGGTGAGGAGATGATGATG | 51 |
| cyse200 | EU907066 | F:CATCATCATCTCCTCACCTT  R:AACATCAACTTACGGTCCAG | 55 |
| cyse204 | EU907070 | F: CGGAACAATGAATGGTAGAT  R: CTATTGAGCTGAGGTTCCTG | 55 |
| cyse207 | EU907073 | F:ACAGCGGAGGTGACGCTTAC  R: GCCAAGACATGCAGACCATA | 60 |
| cyse210 | EU907076 | F: TGCTGAGCCTGATACCTGTG  R: CTGACGTCCTGAAGTGTTCG | 60 |
| cyse213 | EU907079 | F: AAATGGAGAGGCGTGAAGAA  R: GGAGCCACCATTGTCAGTTT | 60 |
| cyse214 | EU907080 | F: CGTGACATGGTCCTCACATC  R: ACAGTGGGGAAACACAGGTC | 60 |
| cyse223 | EU907089 | F:TCTGGAGACTTGAAAACCAT  R:AAAGTTCTGCACGTTTCTGT | 55 |
| cyse225 | EU907091 | F: CAAAACACCACCGTATGTGC  R: TACATGAGGCGCTCACAAAG | 60 |
| cyse230 | EU907095 | F: CAATCAACCAATCACGATGC  R: CAGAGCGCAAACATGAGCTA | 60 |
| cyse238 | EU907103 | F:GCACGACAGCTAACCAATCA  R:CATCCACATGACGGAAACTG | 60 |
| cyse241 | EU907106 | F: TTTCTCTCGGTGAGCGAAGT  R: CCAGCTGGAGTGTGTGTTTG | 60 |
| cyse243 | EU907108 | F: TCATCAGTGTCAGACGTGTT  R: TGGATGGATGGATAAAGAAG | 60 |
| cyse245 | EU907110 | F: TGAAAGAACATGTGGCAAAGA  R: GGGGACCTTCCAAAGAAATG | 60 |
| cyse251 | HM060545 | F:GAGGGAAGGACGAGGAGAGT  R:TTGTTGCGGCTCACTTGTAA | 60 |
| cyse258 | HM060551 | F:CTGGTGTATGACTGAGGGATGA  R:ATCAGGCGACCGTAGTGATT | 60 |
| cyse264 | HM060556 | F: GTGGCGTAGACTCCTGTTCC  R:AAAAGAAAGCTGCCCAGTCA | 60 |
| cyse266 | HM060558 | F:GGTATCGGTTGATACTGGAA  R: CTTCATACGACTGTTGAACG | 55 |
| cyse267 | HM060559 | F:CTGCACTCGACACCTGTGAT  R:AGCCACTTAGCTCTGCTGGA | 60 |
| cyse268 | HM060560 | F:CGATGTTTTAGCATCAGTGG  R:CCATCGTTGTCCTTCCTTAG | 57 |
| cyse269 | HM060561 | F:GTGGGTGTGTCCTCCAAAGT  R:TCTGTCATCCAGCAGCAGTT | 60 |
| cyse270 | HM060562 | F:CGTAAATCTCTGGGGAACGA  R:AAAGTTATTCCCCGGCTCAT | 60 |
| cyse273 | HM060565 | F:GCTGGTTCTGTCTGGATGGT  R:TAAAGGATCAGTCCGCAGGT | 58 |
| cyse274 | HM060566 | F:ACCCTAACCCTAACCCTGTA  R:ATGGTCATCCTCACACACTC | 55 |
| cyse275 | HM060567 | F:ATCTCAATTTTCCTGCCACA  R:CTTCACCCAAGTTTTGGCTA | 58 |
| cyse277 | HM060569 | F: AGGTGCGACTTCTGCTTTTC  R:AAAGGAGCAGATGGCTTTGA | 60 |
| cyse278 | HM060570 | F:GGTGCGTCAGAAAGTCTCAT  R:TTTTCTCGCGCTCTTTGTAT | 58 |
| cyse281 | HM060573 | F:TTGTGTCCATCCTTGTTCCTT  R:TCCGAAACATTCTGAAAAAGC | 58 |
| cyse284 | HM060576 | F:ATGCTGGTTTTCCTGCATTC  R:TCAATGAGAACGCTTTGTGC | 58 |
| cyse288 | HM060580 | F:AAGCTTCACCGCTCTGTCAC  R:CCAAGGTTCCATCCATCATT | 58 |
| cyse289 | HM060581 | F:CAGTCAAGCAGGACCAGTCA  R:CATGAAAGCCAGTGAGAGCA | 58 |
| cyse291 | HM060583 | F:GGGACTTGACGTTGATTGAC  R:CGCAAGAAATGCTGAGAGTT | 58 |
| cyse292 | HM060584 | F:TTTATCTCAGCCAGCAGCAA  R:CCACGGACAACGCACTTTA | 58 |
| hncse1 | GU017678 | F: AGTACAGTAGAGCTGGAGATGC  R:AATGGCAGAAGCAATAACAC | 52 |
| hncse3 | GU017679 | F: AGCATCACTGTCCTCCTCGTAC  R: GCCATGACGACCCTCCCT | 54 |
| hncse11 | GU017683 | F: TTGCCCCACAAACACTCATT  R: CCGCTCTGGGTGTAATGGAA | 51 |
| hncse14 | GU017684 | F: ACAGTTGGCTCTGCTTATCTTC  R: CCCCTGTAACTCTTCTTTATTTG | 52 |
| hncse22 | GU017686 | F: AAACGCAGAGGAATCGGACA  R: GGCCCAGAAGAAATGACAAGAG | 54 |
| hncse26 | GU017687 | F: GTGTCTGTCATCGGTTGTTG  R: CACAAAGCAATGAATGAACG | 52 |
| hncse27 | GU017688 | F: GTTAGGGTTTGTTATCACTGGAA  R: GGCGACAACAACCCTTCAA | 50 |
| hncse29 | GU017689 | F:TACATCCATCAGCCTTACACAT  R:GGGGAACGGTGTTTTCTATC | 52 |
| hncse38 | GU017692 | F: TCCGTAAAAGCAGGGAAAAC  R:CTTCTCCACCTTAGTTCTTGATG | 52 |
| hncse43 | GU017696 | F: AAAGGACGCCCGAGGAGAG  R:GCACTCCCTCAAAGTCTATCACA | 53 |
| hncse45 | GU017698 | F: AGAAGCACATCTTACAACCC  R:GAATGCAGCAATCAATAAGG | 50 |
| hncse47 | GU017700 | F: AGTTGGCGATTTGAGTGGAT  R:CATATTGGAAGTGTATTGAGCAG | 51 |
| hncse48 | GU017701 | F: CACATTCAAATATGGAGGCG  R:CGGGACGGATTTACATTCTC | 52 |
| hncse63 | GU017703 | F: CCAGAGGGAAGTGAGGAAATC  R:TTGTGGTTGGTCGGGTTATG | 54 |
| hncse64 | GU017704 | F: CGGTATCAAACCCTACAAAC  R:TTTGATGAAGAAAGAACGGA | 51 |
| hncse67 | GU017706 | F: TGGGAGGCTGTGACTGTATG  R:CCCGTTCCTCCATTATCTGC | 56 |
| hncse68 | GU017707 | F: GGCAGAGCAGAGAACAAAAAG  R:GCCGCTCTGTTGCTATGGG | 54 |
| hncse72 | GU017709 | F: AATCAGCGGAGGTGAATGGT  R:GCTGCTTCTTTACGAACCTCAC | 53 |
| hncse74 | GU017710 | F: CGCGACTATGAGTCCTGAGT  R:AGGACAGTGAATCCAGGTCAT | 51 |
| hncse82 | GU017713 | F: CAATAATCAGCACCGACAGCG  R:ACAGGCAGGAAGGGAGCGT | 57 |
| hncse83 | GU017714 | F: CATCAACGATCCGTGACTTC  R:CTCTATGGAACCGCAAAACA | 50 |
| hncse85 | GU017716 | F: ACTCACTGCTGGCATGTTGT  R:AGACTGCCTCAAGACAAACTG | 51 |
| hncse86 | GU017717 | F: CACAGTTTACAGTGAGCCTTTG  R:TACTGAGACAGAACGGGCAT | 51 |
| hncse87 | GU017718 | F:CCACCAAATAGAGGGAAATA  R:AAATAAATCCAGGGCAGTCT | 49 |
| hncse88 | GU017719 | F:CGAGGGAAAACAGGGAGAAT  R:TGGATGTGCACTGTTGAAATTC | 52 |
| hncse90 | GU017721 | F: CGGCTCTGAACAACTCATCTA  R:TTGGAAACCTGGGTGAAGC | 52 |
| hncse91 | GU017722 | F: :ACCAGAGGAGGAGAAGGCACG  RAGCTCAAGGGCGCTGATGAC | 54 |
| hncse99 | GU017723 | F: AAGGGTGAGACTTTTACTGCC  R:GAGCAGCAGGTTGGATTTAG | 53 |
| hncse100 | GU017724 | F: CAGCGGGACAGACAACCAAA  R:CCACCTTTTTTGTTGTAGCCTG | 54 |
| hncse101 | GU017725 | F:ATAAAACAGAACGTGTCAGGGATC  R:TTTGGAAACCTGGGTGAAGC | 54 |
| hncse104 | GU017727 | F: TTATGGGAAACCAGAGCAGG  R: TCGGCTCTGAACAACTCATCTA | 52 |
| hncse106 | GU017729 | F: GAGTGACGCAGACCAACAGA  R: CTTGCTGACAGACACTTTTGG | 52 |
| hncse116 | GU017731 | F: CACTAAAACTCCCTGTCTCC  R:TGCTCCTGGCAAAAGAT | 49 |
| hncse120 | Unknown | F: TCCCCACATCAGAAAATGGT  R: CACGGAACAGATCAAACTCTCA | 55 |
| hncse127 | GU585771 | F: TGATGTTCTGTCCGTTCTGC  R: CGGGACACCAGAGATACAGT | 53 |
| hncse130 | GU585773 | F: TTCCTTATCAGGCTGTGGT  R: GCTAAATCAAAATGACCCTG | 52 |
| hncse131 | GU585774 | F: GCATCGGGTGAATAGAAGTA  R: TCAATCAACAGCAAGACCA | 52 |
| hncse135 | GU585776 | F: TTTAGAAACTGTGGGTGAGGA  R: GTTGAGCAGCAGAGTAGGGA | 51.5 |
| hncse148 | GU585784 | F: TCAGTTGCTCAGCGTCCAGA  R: AACACCAGCTGGTACAGACGG | 55 |
| hncse150 | GU585785 | F: CCTCCTACACCCACTTGACA  R: TCCTGCTGTAAACTCTGAACC | 55 |
| hncse156 | GU585786 | F: GCCAGCAACACTAGCACCAC  R: TGCGACCATCCATTTGTTGT | 58 |
| hncse160 | GU585789 | F: AACAGCGACCTGCCAAAACA  R: CCTGCTCGTCCTCGCTTACC | 57 |
| hncse163 | GU585791 | F: GTGCAGTATGTAATGTATACCGTG  R: ACTGACCACAACGCCACA | 52 |
| hncse167 | GU585792 | F: GCAGTGTTGGCAGAGGGAGA  R: ACAGCTCTGGCATCTCCTTCC | 56 |
| hncse169 | GU585793 | F: AACGGGTGTTCATTTCCTTC  R: CACGCTCTGTCTCCTCCTCT | 54 |
| hncse170 | GU585794 | F: GCTGGCGTGTGTCACTAACTTCA  R: ATCAGTGGAACGGGAGGACG | 57 |
| hncse174 | GU585796 | F: TTCAGGGTAAGGCAGAGGGC  R: TGTAGGCGTGTTTGCCGTTT | 55 |
| hncse176 | GU585798 | F: GAAAAATGTCCCCACAACTAAC  R: GTCTGAGCAGTGAGCGATG | 54 |
| hncse177 | GU585799 | F: CACGACAAGAAGCCGAAGG  R: TTGCTCCACAGTGAAACCAG | 54 |
| hncse178 | GU585800 | F: ATCATGGTGCTCAGCGCG  R: GCATAATAGTGTTTGCGTTGAGC | 54 |
| hncse179 | GU585801 | F: CACCACCTTCTTCACTTCCC  R: CCATCTTCGGCCACTCTGT | 57 |
| hncse181 | GU585803 | F: AGCATAAGGTCACGGGTTTG  R: TGTTGCAGTCTGTTCAAATTCTTC | 51 |
| hncse182 | GU585804 | F: TGTTAGCCTCATAAAGTCCTG  R: GGACAACGAAGAACACCAGA | 53 |
| hncse183 | GU585805 | F:AGGTGTCGTGATACTGAGTGGGTT  R: CTCCCTCCACGGTCCTTCAT | 53 |
| hncse187 | GU585808 | F: AGTGGCTTCACAACAAACAGGT  R: CGCTTTCTGATCTGAGTTACGAC | 53 |
| hncse189 | GU585809 | F: CTAGAAACTGTGGGTGAGGA  R: AACAGAGGCTGAAAGAGTGA | 50 |
| hncse198 | GU585812 | F:GAGCAGTGAACAATAGACGGGATG  R:AGCGAAATAAAGTCAGCAGCATCT | 54 |
| hncse201 | GU585813 | F: AGTCTCTGAAAGTGCCTCCGTA  R: CTCGTTGTTCGTTCGTTCGT | 52 |
| hncse203 | GU585814 | F: CCCCTCTTTTCAACCCCTAC  R: CCAGCCTGAGGGAAACATC | 54 |
| hncse206 | GU585816 | F: GCAGTGAAGAAGAGACGACAT  R: GTGGTGGGTTGATGTATTGC | 54 |
| hncse217 | GU585822 | F: CACAGCGTCCTTCAATCTCCA  R: GCCCACTTTCCATCGACGT | 55 |
| hncse220 | GU585824 | F:CGGTGGTGGGTTGATGTATTGC  R:CTGTGGCGCCGGTCCTCT | 57 |
| hncse221 | GU585825 | F: GGAACACACAGCAGCAGCAG  R: AGCATAGAGCCAATCCCAAATA | 53 |
| hncse229 | GU585828 | F: AAAACATCCGTCCATCCCA  R: GAGTTTCCTGAGGCGCATAGT | 55 |
| hncse231 | GU585829 | F: AAGGGACAAAATGATGAAGA  R: CGACTCTCTCTCTCTTTGTGT | 52 |
| hncse233 | GU585830 | F: CGATGACTGGTTATGAACTTGT  R: GACCGACTGTTGAAGGGAG | 53 |
| hncse236 | GU585831 | F: GATGTTGTCAATGGAAATGTG  R: CGTCCACAGAAGATACCCA | 51 |
| hncse241 | GU585832 | F:AGTCCCATCAAACCTCACATT  R:GCCCCTGAGTGTTTGGATA | 52 |
| hncse247 | GU585833 | F: GCACAAACCAGCAAATCCAT  R: CAGGCAGGCTGTGATAAGGTC | 52 |
| hncse252 | GU585835 | F: ATTGTTTTCCTTGCCTTTTA  R: ATACTGTTGTCTCCTGTGGC | 52 |
| hncse258 | GU585839 | F: GGAGAGATATGGTCGATAAAGAA  R: AGCTTCCAACCTGTTGACTG | 52 |
| hncse262 | GU585840 | F：CGCTTCTGTAACAGGACCTT  R：ACTGACACCAGACCTCCACA | 54 |
| hncse273 | GU585843 | F: CTGAGTAGCCTCCTCCAAGA  R: TCGTGCCTTATTTTGACTGT | 51 |
| hncyse3 | GQ426756 | F: TAAGCAGATTTGGTGTCCTC  R: CTGGGTAGGTGGTTTTGG | 50 |
| hncyse4 | GQ426757 | F: GGTGGCTGGTGAATAAACAA  R: CATGAACAGTATGGGAAAGG | 51 |
| hncyse5 | GQ426758 | F:TCCGCCCACAGTGACAAGT  R:AGCCCAGCTATCTGCCTGTC | 55 |
| hncyse9 | GQ426762 | F: CATCAACGGAATGCGGTAA  R: TGTAAGTCTCACTGCGGGTC | 51 |
| hncyse11 | GQ426763 | F: AGAATCCGGTTGAGACACG  R: AGAGCCTGTCATCAACGGA | 52 |
| hncyse13 | GQ426764 | F: GGACTGACTGAAGGAGGAAAC  R:CTTCCCTGATTGTGACGGT | 51 |
| hncyse28 | GQ426778 | F: ATGCCGCCACGGGCACTG  R:GGTGGAGGTAAATGAAGCAGGTCG | 55 |
| hncyse31 | GQ426779 | F: CTCACCCTGAAGTACCCCATC  R: TGTTGGCCTTGGGGTTGAG | 55 |
| hncyse33 | GQ426780 | F: AGCGGAAGCAGCAGAAGGAT  R: A CGAAGGGCGGAGAAGTCAG | 55 |
| hncyse37 | GQ426782 | F: CGCAGGCTGCATAAACGA  R: CCTCCCTGTGGCTAATACTGT | 52 |
| hncyse44 | GQ426788 | F: AAAGAGGCGTGTCATCGGTT  R:CCCAATCGCTCAAGAAATCC | 52 |
| hncyse47 | GQ426791 | F: ACTGTGAATGTGTGTGTGCG  R:CACATTCTTGAGTCTCGGTA | 55 |
| hncyse48 | GQ426792 | F: :GAGTCTCAGAGCCACATCAG  R CCTGTACAAGATGGACTTTATTAG | 52 |
| hncyse60 | GQ426803 | F: AAAGGAGACCCCTGGATGTG  R:AATGCCTGCGGTAATGGATA | 52 |
| hncyse65 | GQ426808 | F: CCAACGAGCAGGGAGAAGGT  R:GCAAAGCAGGAAAAGGTAGCA | 55 |
| hncyse73 | GQ426816 | F: TAGACCTTCCTTCGCAGCAT  R: AGAAGAGTTAGCTCAATCAACG | 53 |
| hncyse75 | GQ426818 | F: ATGATAAGGGACAAGGTGCT  R:AGATAATGGATGCTGGGGTT | 52 |
| hncyse85 | GQ426827 | F: TTCAATAATGGATGCCAAACTT  R: CCGCAGAGGAATCACTCAAAC | 53 |
| hncyse90 | GQ426832 | F: CTTGAAAACACGCCAACAGTC  R: AGGTCCAGAACCACAGAGGC | 55 |
| hncyse91 | GQ426833 | F: TACCACGCTGCCTTCAAATG  R:TTCACCACTGTTCTGCTCCAC | 54 |
| hncyse92 | GQ426834 | F: CCGAGGGAAGGACACATAGAG  R:CCTCCAGCTGTAAGGACTGACT | 53 |
| hncyse96 | GQ426836 | F: ATCCGTGTGTGTTAAGAAGAGT  R:AGGGCAGAGGAATAAAGACA | 51 |
| hncyse97 | GQ426837 | F: AAATCGCAGACGAGTTTGAA  R:TGGCTTTTATCAAGTGTTAGGC | 52 |
| hncyse99 | GQ426839 | F: GCTCTGTGATCGCTCAGTTGT  R:GGACGAAGCAGAAGAAAAGGA | 52 |
| hncyse102 | GQ426841 | F: GAACCGTTGGCACCGTTTGT  R:AGACGTGGGACCAGGGGAAG | 56 |
| hncyse107 | GQ426846 | F: TCGCTCGTTTGGTATCTGTTG  R:CAGAAGATTGGCTGAAGGGA | 52 |
| hncyse108 | GQ426847 | F: ATCACATTAATGTGTGTGTCTGTG  R:CTTCTCATCACCTGCCCTTT | 50 |
| hncyse110 | GQ426849 | F: GACGAAGGTTTTTGAATGGTTA  R:GTGAGGCACAGGCTTTTGGA | 53 |
| hncyse113 | GQ426852 | F: AATCACTCCAGCTTCAACCC  R:ATGACTGTTCGACTCTGCTTCT | 54 |
| hncyse115 | GQ426854 | F: TCCGACCCACAGTTGTATCA  R:TAAACAGATCTGCTGCAGTGAGT | 53 |
| hncyse119 | GQ426858 | F: ACCAGGAGACTCAACCCACC  R:AGACAAATGTGGTAGGAGGGAG | 54 |
| hncyse124 | GQ426863 | F: TCATCAGCCCAGTTCAAAGT  R:CTACCTCTGCTTCAGCTGTG | 52 |
| hncyse125 | GQ426864 | F: GTAATTTTACAGCAGGTGCC  R:CAGTTCTCCACGAGTCTATTGTT | 51 |
| hncyse129 | GQ426867 | F: TCTTTACCGAGAATCTCCCTCC  R:AAAGGGCCTACCGAAAGAAT | 53 |
| hncyse130 | GQ426868 | F: AGAGAAATGAGAGTCAGCTAGAG  R:TGGTTACACGCTGTCACAAA | 52 |
| hncyse133 | GQ426871 | F: ATGTCAAACCAGAGCCAACC  R: GCAGTCAAGGTCCACAGTTT | 49 |
| hncyse136 | GQ426873 | F: TCCATTCAGTGTTGGGACAG  R:TGATGTCAGCCCACAAAACA | 60 |
| hncyse139 | GQ426874 | F: TGGTTCCTTCGTTCCCTTTC  R:CTGGGGAGCAGTATGGTTTT | 53 |
| hncyse141 | GQ426876 | F: TTGTCCTTGGTGCTGCTCTG  R:GCCTCACTTTGACCTGTTTGC | 54 |
| hncyse147 | GQ426882 | F: CAGGAGGTTTGGGCTCTTGA  R: GTTCCCCAAACGGAGTGAGA | 53 |
| hncyse148 | GQ426883 | F: GAGCGGAGCTGTGAGTTTGA  R: CTGAGTTCAACAGCAGGAGG | 53 |
| hncyse150 | GQ426884 | F:AAAATGGTGCTGCCAAATAC  R: ATGGTAGACACCCGCTCACT | 53 |
| hsts-1 | EF363858 | F: AGGACGACTTCTTCTTCAAAC  R: CTCGGGTTCTGGTTCTGC | 51 |
| hsts-3 | EF363860 | F: GTCGCAGCACAGATAGGG  R: GAGGCAACAAGTCGGTCA | 52 |
| hsts-4 | EF363861 | F: AATGCGCCACAGAGGATC  R: TGAGGAGGAGGGAGGAAA | 52 |
| hsts-11 | EF363868 | F: GAATGAGCGGTGGAATAAAT  R: TTGCCAGAATCAGAACGACA | 53 |
| hsts-A | EF439656 | F: TAGCCACAAAAGTAGAACGAAT  R: CTAATCCAACAAACACCAGTAT | 51 |
| hsts-B | EF439657 | F: ATTTATCAACGTCCACAGCA  R: CAGTCAGTCAGTCCGTCAGT | 50 |
| hsts-C | EF439658 | F: GTGTCTCATCACCTTTTCCT  R: CACCTGTCATCTTTTCGTTA | 49 |
| hsts-e | EF439660 | F: CATACGTGACCTGCTACC  R: GAAGAAAGAGTCGGAAAA | 46 |
| hsts-f | EF439661 | F: GCTTTGATGTACCGCCCACC  R: ATCGACCAATCACAGCTCAGGAT | 55 |
| HSTS-g | EF439662 | F: GTGTATGTAAACAGCCTCTG  R: TATAATTTGCGTCTGGAGTG | 48 |
| hsts-h | EF439663 | F: ACTTGGCTTCAAATACACTCTT  R: GTACCGATCATATCCTGCTCTT | 50 |
| hsts-J | EF439665 | F: CAGCATCAACCTGGAGAACA  R: AGACAGAAGACTGGCAAGAAG | 52 |
| hsts-k | EF439655 | F: CTTGGCACAGGACAACACTT  R: ATGAATGAATGCTTGAACCC | 51 |
| Veva04 | EU599440 | F: TCTACACGGCTGGTGTCTTG  R: ATCCACAAACACATGGCTCA | 56 |
| Veva24 | EU599460 | F:CACACACAACACCACAACCA  R: AAGCGAGGGATGAACAGAGA | 55 |
| vmo8 | AB110621 | F: TCCAGGTATGCCGTTTGC  R: CGTTCCCAGTGTGAGACATTT | 53 |
| gene072 | FJ418072 | F: TGAGTCCTCGAACCTTCCT  R: ATGGCACAGTGGTGTCCTAT | 51 |
| gene177 | EF421177 | F: TGAAGCCACCAGTGGATGAT  R: TGGGTCGTGAAGGTATGTGA | 57 |
| gene665 | EU007655 | F: GTGGAAATGGGATGGATGAC  R:TGGAGGGAATGTGGACAAAG | 55 |
| gene116 | EF683116 | F: AGTTGGTTTGAGAAGAAGGT  R:TGTACTGGGCAGTAACATTA | 51 |
| sox9-1 | GQ402461 | F: CAGGCAGGTAATGTTGGGGT  R: AAGGAGCCGTAGGTGATGTG | 57 |
| ghrh-ssr | FJ608666 | F:TTGTTTGCCCGTGAGACT  R: CGCTTTTCTGAGGGATGT | 54 |
